# High-resolution mapping of artificial reefs in southern California: Linking sonar-derived habitat metrics to reef construction material and design

**DOI:** 10.1371/journal.pone.0357087

**Published:** 2026-08-26

**Authors:** Jeremy T. Claisse, Matthew H. Kim, Chelsea M. Williams, Natalie P. Shubin, Casey B. Pua, Daniel J. Pondella

**Affiliations:** 1 Department of Biological Sciences, California State Polytechnic University Pomona, Pomona, California, United States of America; 2 Vantuna Research Group, Occidental College, Los Angeles, California, United States of America; Central Marine Fisheries Research Institute, INDIA

## Abstract

Artificial reefs can be designed to support ecosystem functioning by optimizing habitat variables. Unfortunately, fine-scale quantitative descriptions of their habitat structure remain limited, especially in California. We collected high-resolution sonar bathymetry and backscatter (0.3 m cells) across 13 artificial reef complexes and shipwrecks along the southern California coast. There were 154 delineated reef modules using an unsupervised habitat-classification workflow that integrated bathymetry-derived slope and vector ruggedness with backscatter. Most modules were primarily composed of quarry rock (n = 84) or concrete pilings/light poles (n = 45), with additional concrete rubble modules (n = 14), barges (n = 4), shipwrecks (n = 3), and a missile tower (n = 1). This approach generally resolved boundaries, but irregular and discontinuous modules built from primarily scattered concrete materials (e.g., pier pilings, light poles, dock floats) lacked clear edges and often included interspersed soft bottom, rendering their mapped footprints more subjective as opposed to contiguous materials (e.g., quarry-rock piles, barges, shipwrecks). For each module we calculated footprint area, volume, mean and maximum vertical relief, mean slope, mean rugosity, and standard deviation heterogeneity metrics for relief, slope, and rugosity. In multivariate analyses modules generally grouped by construction material and design (artificial reef complex), indicating that these metrics capture ecologically relevant structural differences. Quarry rock modules generally exhibited higher mean relief, slope, and rugosity over more compact footprints, whereas scattered concrete modules typically had lower mean complexity but greater rugosity heterogeneity, driven in part by extreme low and high values created by interspersed patches of flat soft bottom amongst concrete materials. The resulting maps improve positional accuracy relative to historical maps and our approach provides a workflow for quantifying reef habitat structure, enabling analyses informing the design of artificial reef projects.

## Introduction

As the use of artificial reefs for habitat restoration and mitigation is expanding, there is an increasing design opportunity for ecosystem enhancement [1–3]. To achieve this, we must effectively monitor and characterize the physical and ecological attributes of existing structures [1,4,5]. Systematic efforts have been made to estimate the footprint (i.e., area of seafloor) covered by artificial reef habitats in the United States [6–8], which has increased approximately 20-fold from 1970 to 2020 [8]. Beyond simply locating and delineating features, sonar-derived bathymetric data can be used to derive quantitative habitat metrics (e.g., relief, slope, rugosity) and compared to patterns of species abundance, community composition and ecological performance across a range of marine taxa [9–11]. These habitat-biota relationships can then be applied to design artificial reefs or other forms of built marine infrastructure [e.g., energy generation, ports, aquaculture; 12] to enhance ecological performance [3].

Artificial reefs worldwide have been constructed with a variety of construction materials and designs [5,13–16]. Ramm, Florisson (15) identified 1,074 unique artificial reefs across 71 countries. Most intentionally constructed (n = 921) artificial reefs were built from concrete, followed by steel, and then rock, with over half of all reefs being built from “materials of opportunity” (i.e., opportunistically sourced, repurposed materials). Quarry rock boulders are the most widely used natural material due to their availability, stability and ecological suitability [2], and can also be considered more environmentally acceptable [17]. Other materials of opportunity, such as tires, have proven unstable and environmentally detrimental [18]. Assessing how sonar-derived habitat metrics vary among material types may be similarly informative, given that artificial reef construction material can influence the resulting reef-associated biota, affect fish assemblage structure and related ecological metrics [5,16,19,20].

The artificial reef complexes examined in the present study represent a compilation of available high resolution (30 cm) sonar data from southern California reefs mapped during multiple state and federally funded projects that each had distinct objectives, geographic priorities, and logistical constraints. Consequently, the study reefs are concentrated primarily off the coast of Los Angeles and Orange counties, with the addition of International Reef near the California–Mexico border. While representing only a subset of artificial reefs in the region, the included reefs span a wide range of construction histories, material types, and module designs. The California Department of Fish and Game (now the California Department of Fish and Wildlife, CDFW) initiated experimental artificial reef projects in the late 1950s to enhance sportfishing opportunities and promote giant kelp growth [21]. Early experimental reefs, mostly in Santa Monica Bay off the coast of Los Angeles, incorporated a diverse range of materials, including street cars, automobile bodies, quarry rock, and concrete shelters, arranged in a variety of designs to evaluate material performance [22]. In the 1980s, additional quarry rock reefs with more replicated module designs were constructed, resulting in larger artificial reef complexes such as Santa Monica Bay and Marina del Rey 2. Later in the decade, construction of the Bolsa Chica artificial reef complex began using primarily a variety of concrete materials of opportunity (e.g., pier pilings, telephone poles, rubble) and steel/concrete barges, followed by substantial augmentation with additional material throughout the 1990s [23]. Additionally, construction of the International Reef complex near the California–Mexico border began in the 1990s with a missile tower and quarry rock modules, and was subsequently augmented in the early 2000s with additional quarry rock and concrete poles and pier pilings [23]. These existing CDFW historical fishing reefs, combined with additional shipwrecks and a recent habitat restoration reef [Palos Verdes Reef; 24,25] collectively encompass a wide range of construction materials and designs, from discrete piles of quarry rock, degrading ship hulls or steel and concrete barges to scattered fields of concrete material. This substantial variation provides a valuable opportunity to investigate how sonar-derived habitat metrics vary with reef material and design characteristics.

**Fig 1:**
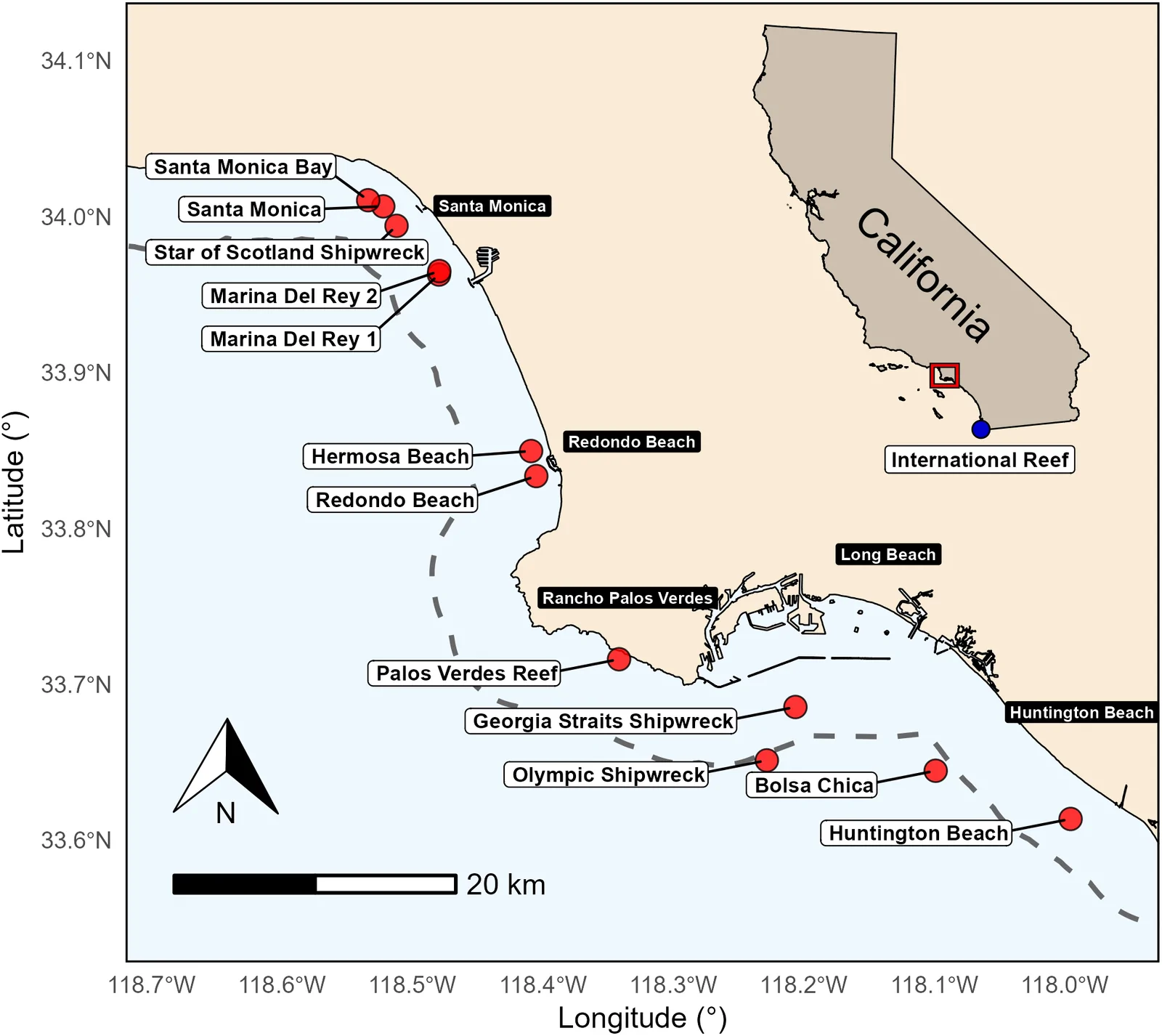
Locations of the 13 artificial reef complexes and shipwreck reefs in southern California that were surveyed with sonar to map their artificial reef modules and quantify their habitat characteristics. Note the location of the International Reef Artificial Reef Complex near the United States—Mexico border is not included in the main map but by the blue circle on the California overview map. The boundary between state and federal waters (gray dashed line) is generally located 3 nautical miles offshore.

This study characterizes the current physical extent and quantifies the habitat features of several artificial reefs along the southern California coast, making them available for subsequent studies to assess these metrics against complementary biological survey data. We collected high-resolution (30 cm) bathymetry and acoustic backscatter across 13 artificial reef complexes and shipwrecks (Fig 1), using historical maps and descriptions [primarily 23] to guide module locations, naming, and initial construction material determination. We then applied a habitat-classification workflow to delineate the spatial extent of individual artificial reef modules. From the bathymetry, we calculated quantitative habitat metrics to describe the physical structure and complexity of the artificial reef modules. We evaluated relationships among these metrics to identify redundancy, complementarity, and their distinct contributions to describing structural complexity and heterogeneity. Finally, we compared modules by construction material and design (i.e., artificial reef complex) to group those with similar characteristics and to begin to assess the effectiveness of these sonar-derived metrics to capture ecologically relevant variation in habitat structure. Together, these analyses provide an up-to-date assessment of several existing artificial reef habitats in southern California and builds a foundation to inform the design, monitoring, and management of future artificial reef projects.

## Materials and methods

### Sonar data collection

From 2022 to 2024 high-resolution (30 cm) bathymetry and acoustic backscatter sonar data were collected for the areas encompassing each artificial reef complex or shipwreck using small (6.5 m – 7.5 m) vessels with a pole-mounted EdgeTech 6205. The 6205, a Multi-Phase Echo Sounder (MPES), produces real-time, high-resolution bathymetric data while providing co-registered simultaneous dual frequency (540 kHz [sidescan and bathymetry] and 1600 kHz [side scan]) imagery. Geophysical surveys were conducted under California State Lands Commission General Offshore Geophysical Survey Permit #A4502. Each survey consisted of parallel transects 60 m apart with a swath width of 120 m to ensure 200% coverage. In SonarWiz 7, bathymetric (depth information measured in meters on a negative scale) and acoustic backscatter point cloud data were manually cleaned, post-processed, and output to 30 cm rasters for analysis.

### Artificial reef habitat classification and module delineation

**Fig 2:**
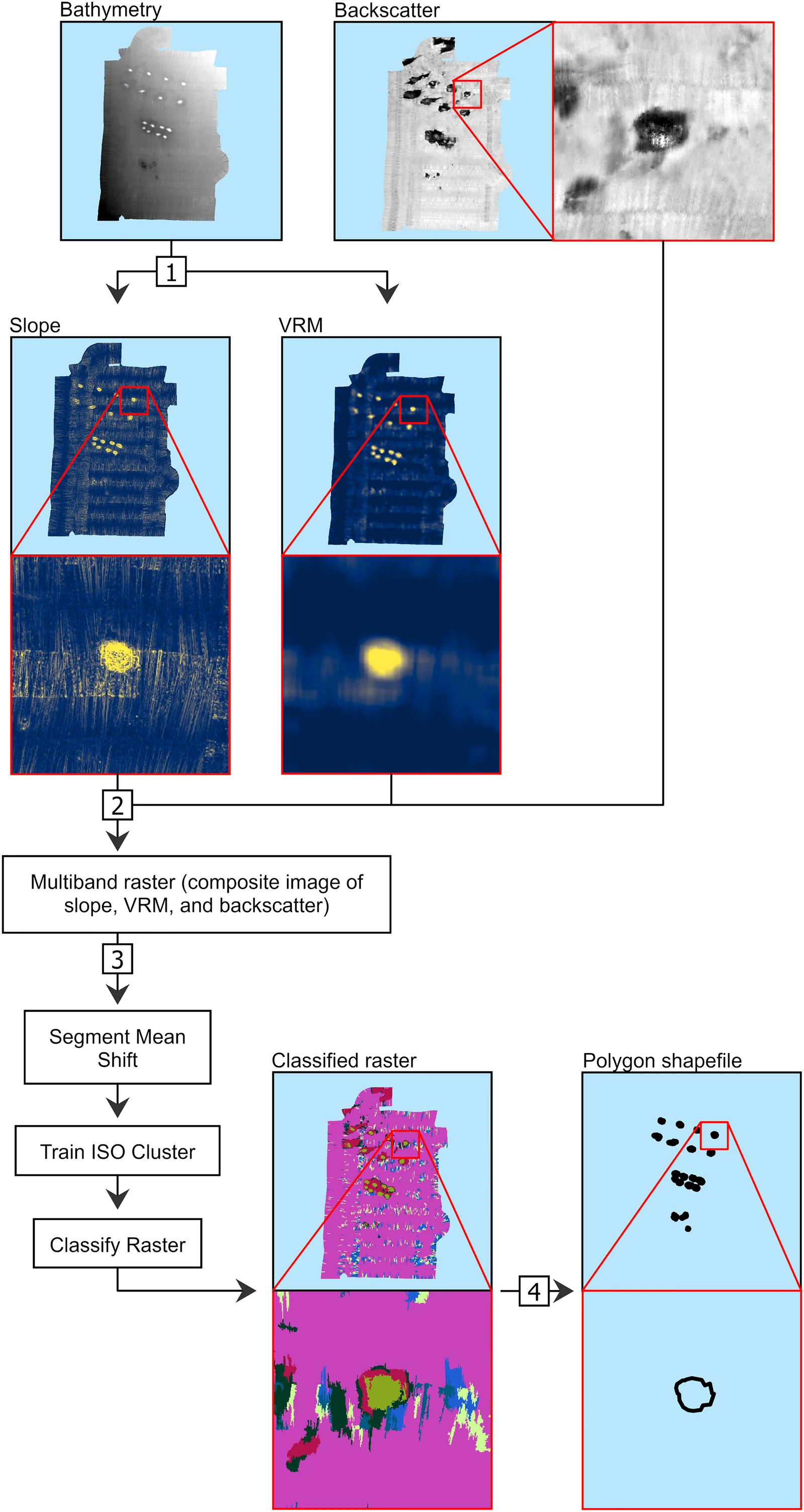
Protocol for determining the extent of artificial reef modules through habitat classification of sonar derived bathymetry and backscatter rasters. (1) Bathymetry is read into R to calculate fine-scale slope (3 × 3 cells) and broader scale VRM (31 × 31 cells) rasters using functions from the R ‘MultiscaleDTM’ package; (2) slope, VRM, and acoustic backscatter rasters are mosaicked into a single composite raster using functions from the R ‘terra’ package; (3) the multiband raster is loaded into ArcGIS Pro as the start of several steps to produce a classified raster; (4) the classified raster is simplified in R, then manually edited in ArcGIS Pro where the final version is converted into a polygon shapefile to calculate habitat metrics for individual artificial reef modules.

We adapted existing seafloor habitat classification processes using bathymetric and backscatter data [26,27] to determine the seafloor area footprint of the artificial reef modules. Seafloor habitat classification was conducted using a combination of R functions and ESRI ArcGIS Pro tools to identify and delineate seascape features and generate polygon outlines for each module (Fig 2). Inputs for the classification were created from gridded bathymetry and acoustic backscatter data. Acoustic backscatter, generally interpreted as hardness (0–255; higher values indicate reef substrate), helped distinguish hard reef from surrounding soft sediment [26,28,29]. Slope and vector ruggedness measure (VRM) rasters were also derived from the bathymetry raster [following 26, 27] using the *SlpAsp* and *VRM* functions in the ‘MultiscaleDTM’ R package [30]. We evaluated multiple neighborhood sizes for each of these metrics during development of the habitat classification workflow, before selecting the final spatial scales used for classification. Ultimately, we used slope (degrees) calculated at its smallest possible scale with a 3 × 3 cell window around the focal cell (3 cells * 0.3 m cell length = 0.9 m scale) (Table 1). This fine-scale slope raster effectively identified small vertical-relief features associated with artificial reef material, but it also amplified fine-scale artifacts in the bathymetry, likely caused by vessel motion during sonar data collection under surface-swell conditions (Fig 2). We therefore paired the fine-scale slope raster with a broader-scale VRM raster calculated with a 31 × 31 cell window, corresponding to a 9.3 m scale (31 cells * 0.3 m cell length). During habitat classification this broader scale VRM captured larger landscape-scale variation in terrain orientation and effectively muted the fine-scale noise and artifacts that were apparent in the fine-scale slope rasters (Fig 2). In R, using functions from the ‘terra’ package [31], the backscatter, fine-scale slope, and VRM rasters were then merged into a single multiband raster, which served as the input for the habitat classification process.

**Table 1:** Specifications for complexity and size-related habitat metrics.

	Description	Units	Analysis Window (cells)	Spatial Resolution	R Package	Function
**Habitat Classification Metric**						
VRM	Vector ruggedness measure: the dispersion of unit vectors normal to the terrain surface (Ilich et al. 2023). The large spatial resolution we chose (31x31 cells) captures larger landscape scale variation in terrain orientation.	unitless	31x31	9.3 m	MultiscaleDTM	*VRM*
Fine-scale slope (0.9 m)	Maximum rate of change in depth values (Ilich et al. 2023) at a small (3x3 cells) 0.9 m scale.	Degrees	3x3	0.9 m	MultiscaleDTM	*SlpAsp*
**Habitat Characterization Metric**						
Relief	Vertical relief. Note the highest and lowest 1.5% of values within module were extent excluded from mean and max relief calculations.	m		0.3 m cells within polygon shapefile		
Module-scale slope (2.7 m)	Maximum rate of change in depth values (Ilich et al. 2023) at a medium (9x9 cells) 2.7 m scale.	Degrees	9x9	2.7 m	MultiscaleDTM	*SlpAsp*
Rugosity (2.7 m)	Adjusted standard deviation: terrain roughness after accounting for slope (standard deviation of residual depth values from a slope plane fit across the focal window) [30] a medium (9x9 cells) 2.7 m scale.	m	9x9	2.7 m	MultiscaleDTM	*AdjSD*
Footprint	2D area of module extent.	m^2^		0.3 m cells within polygon shapefile	terra	*expanse*
Volume	Sum of volumes of each cell (cell area * height above estimated surrounding seafloor depth) in raster within a module.	m^3^		0.3 m cells within polygon shapefile		

Metrics were calculated from sonar bathymetry rasters or polygon shapefiles that were used in habitat classification process to estimate artificial reef module extent or characterization of artificial reef module habitat.

The classification process adopted an object-based approach, which extracts features based on both statistical and spectral similarities between groups of “objects” and categorizes them [32,33]. The process began with segmentation in ArcGIS Pro using the Segment Mean Shift tool (Table 2). This tool uses the Mean Shift approach by grouping neighboring cells that have similar spectral characteristics and replacing each cell within the neighborhood with the calculated mean value [27,34,35]. We then performed an unsupervised classification to the segmented raster via the Train ISO Cluster Classifier tool, chosen because of its simple, machine-driven approach with few manual adjustments. An ESRI classifier definition file was created and read into the Classify Raster tool as the final step in the classification process. The maximum number of classes/clusters in the classified image was pre-defined to accurately classify as much artificial reef habitat as possible (Table 2), but the resulting number of classes varied for each artificial reef complex.

**Table 2:** Specifications for the 3 tools used in the habitat classification process conducted in ArcGIS Pro ModelBuilder to determine the extent of artificial reef modules from the sonar derived bathymetry and backscatter rasters.

Tool	Parameter	Value
Segment Mean Shift	Spectral Detail	15.5
	Spatial Detail	15
	Min Segment Size	200
	Max Segment Size	−1
Train ISO Cluster Classifier	Max Number of Classes	10
	Maximum Iterations	20
	Maximum Cluster Merges	5
	Min Number of Samples	0.5
	Skip Factor	5
Classify Raster	*Default settings used*	

The Segment Mean Shift tool groups neighboring pixels together based on spectral and statistical properties. The Train ISO Cluster Classifier tool uses an unsupervised IsoCluster algorithm to create an ESRI classifier definition file (.ecd). The Classify Raster tool uses the  .ecd file to classify the input raster.

Outlines of spatially distinct modules were created using the classified rasters for each artificial reef complex. In ArcGIS Pro, we selected the classes that appeared to best represent hard substrate and high relief features indicative of reef habitat. The selected classes were converted into a polygon feature class and adjacent polygons were merged before being exported as a shapefile. Polygons were then simplified using the *ms_simplify* function in the R ‘rmapshaper’ package [36] with the *keep* argument set to 0.15 to limit jagged edges and excessive vertices as a result of the classification process based on high resolution (30 cm) sonar data. In ArcGIS Pro, we made additional manual edits to the simplified polygons primarily removing areas resulting from data collection artifacts (i.e., erroneous values due to conditions experienced in the field) in the raw bathymetry grids that were captured and classified within the polygon outlines. Adjacent polygons were also merged when gaps were < 5 m in diameter. For polygons encompassing areas of reef construction materials scattered across soft sediments (often single concrete pier pilings) interior holes were removed. The bathymetry, backscatter, slope, and VRM rasters were also used for visual reference during the manual editing process. Where possible, artificial reef modules were delineated and named to maintain historical spatial designations and names [following 23]. In some cases, typically for modules that contain relatively large areas of scattered concrete material, the polygons (which span multiple historically defined modules) were divided along paths through soft sediment to maintain the historically named module spatial designations.

### Habitat structural characteristic metrics

**Fig 3:**
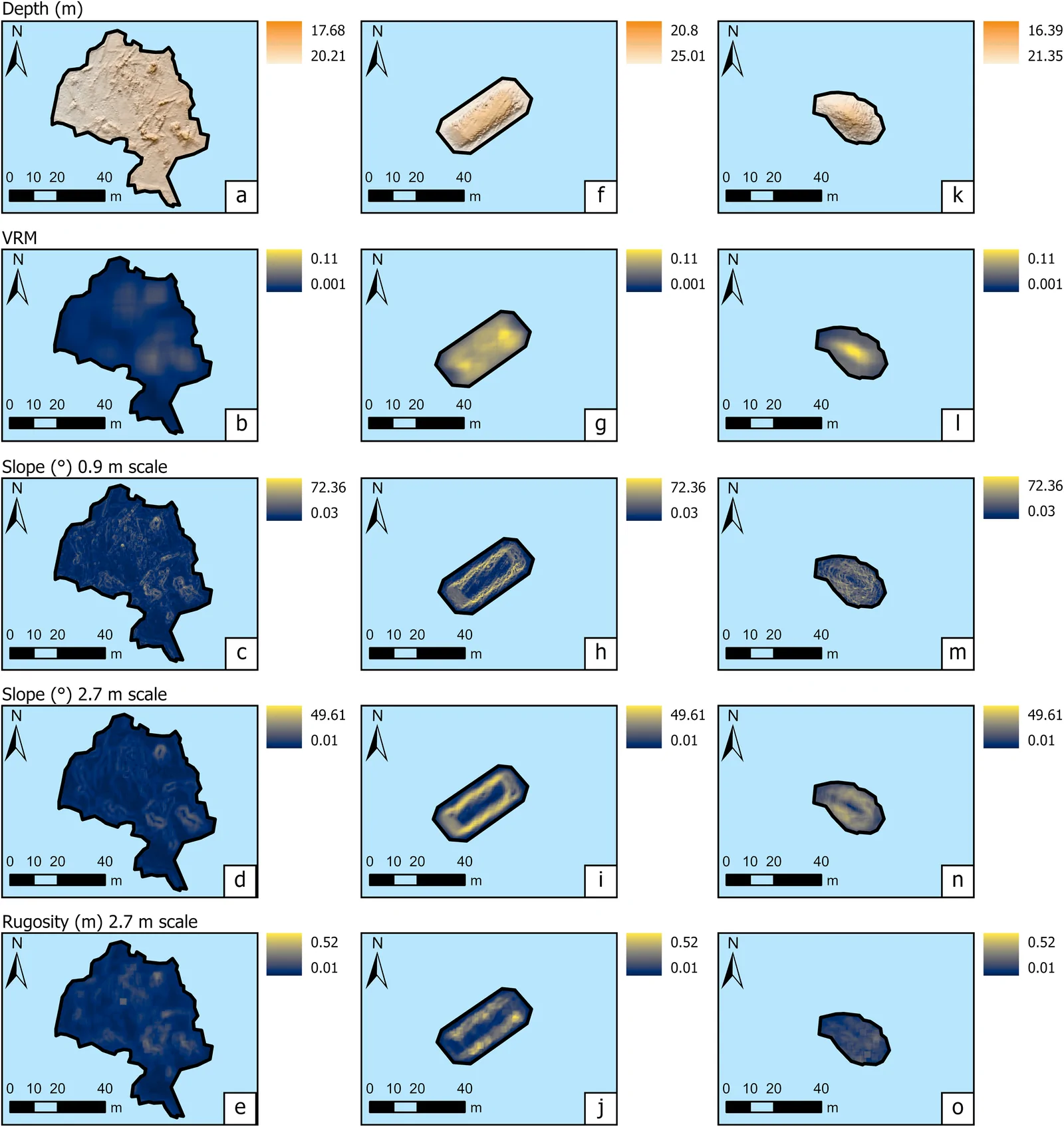
Bathymetry and derived habitat metrics for three example artificial reef modules built from different material types. Artificial reef modules include Hermosa Beach module A (a – e) primarily composed of scattered concrete pier pilings, Redondo Beach module A (f – j) composed of a steel and concrete barge, and Marina del Rey 2 module E (k – o) composed of quarry rock. Depth (a,f,k) displays a hill shade image of the raw bathymetry (30 cm cell size) raster from which the other derived habitat metrics are calculated. VRM (b, g, l) and the fine-scale (3 x 3 cell focal window, 0.9 m) slope (c, h, m) were used, along with backscatter (not pictured) as inputs for the habitat classification process to map the extent of artificial reef modules. Larger 2.7 m scale (9 x 9 cell focal window) slope (d, i, n) and rugosity (e, j, o) were calculated as metrics to compare habitat characteristics among modules.

The finalized module polygon shapefiles and the associated bathymetry rasters, were used to calculate habitat metrics for the individual modules (Fig 3, Table 1). Size metrics (e.g., seafloor area footprint, reef volume) were calculated for each module. Footprint area was calculated as the two-dimensional area enclosed by each module polygon. Volume was calculated by summing the cell-level volumes within each module polygon, with each cell volume estimated as cell area (0.3 m * 0.3 m) multiplied by the vertical height of reef material above the surrounding seafloor (see vertical relief calculation description below for more detail). For modules built from quarry rock, these areas and volumes are largely representative of the extent of contiguous reef material. However, for most modules built from scattered concrete material (e.g., pier pilings) these area and volume estimates are more subjective. Often they were inclusive of relatively large patchy areas of soft sediment among scattered reef material, and/or sometimes a larger area of what the habitat classification process defined as a single polygon was divided to maintain historical module spatial designations and names [23]. Therefore, for analyses related to artificial reef module size, only quarry rock, barge, shipwreck, and some concrete rubble reef modules should be considered contiguous material, while the scattered concrete pier piling or pole modules are often part of, or directly adjacent to, much larger extents of more distributed reef material.

Vertical relief metrics were calculated from the bathymetry raster after excluding the highest and lowest 1.5% of depth values within each reef module polygon. A percentage-based cutoff was selected because module sizes varied widely, allowing the number of excluded cells to scale with module area. The cutoff was determined through trial and error and visual examination of distributions (e.g., S1 Fig), effectively removing short tails of extreme values (sometimes 0.5 to 1 m above or below the main structure) from the distributions of the depth values. These values represented sonar artifacts, small seafloor depressions, or more rare vertical protrusions of the structure. This approach provided more representative estimates of maximum and mean relief, decreasing bias from artifacts or atypical structural elements.

Slope and rugosity metrics were calculated on a cell-by-cell basis in R, using functions from the ‘MultiscaleDTM’ R package [30] across the entire bathymetry raster for each artificial reef complex (see Table 1 for metric and R function specifications). The larger module scale slope was calculated at a 2.7 m scale with the *SlpAsp* function using a 9 × 9 cell focal window (9 cells * 0.3 m cell length = 2.7 m), corresponding to the maximum rate of change in depth across the focal window. We selected this scale to represent the broader angle of the reef surface along the sides of artificial reef modules, making it more appropriate for comparing module-scale habitat structure and approximating structural variation likely to influence reef-associated fishes. Rugosity was calculated at the same scale using the *AdjSD* function, which quantifies rugosity as the standard deviation of residual depth values after fitting a slope plane across the focal window. This rugosity metric therefore quantified finer-scale roughness around the broader slope plane and provided a complementary measure of habitat complexity. This adjusted standard deviation metric, developed in Ilich, Misiuk [30] is expressed in units of meters, making it more conceptually interpretable, and was less correlated with the slope than other rugosity metrics. Slope and rugosity values for each cell within the artificial reef module polygon were then extracted from the derived rasters for subsequent calculations.

For each artificial reef module, we calculated (1) a mean and (2) a standard deviation (sd) of relief, slope, and rugosity based on the extracted bathymetric raster cell values from within each module polygon. Standard deviation provides a measure of heterogeneity of the cell values for each metric within a module; in this context, lower values indicate more uniform habitat structure (e.g., most cells within a module having similar metric values), while higher values reflect greater variability (e.g., a module containing a greater range, and/or more relatively low and high values of the habitat metric).

### Data analysis

Analyses were used to identify patterns in habitat structure among artificial reef modules and evaluate relationships among metrics, modules, reef design and/or construction materials. Seven habitat metrics for each module were included: maximum and mean vertical relief, mean slope, mean rugosity, and the standard deviation quantifying within-module heterogeneity for relief, slope, and rugosity (Table 1). Analyses were conducted using R [37] in RStudio [38], using functions from ‘tidyverse’ packages [39] whenever possible. Pairwise scatterplots, density plots, and Pearson correlation coefficients between habitat metrics were created using the *ggpairs* function from the ‘GGally’ package [40] to evaluate relationships between and identify redundancy or complementarity among metrics. All metrics were standardized (centered and scaled) to equally weight variables with different units or scales for the heatmap, cluster analyses, and principal components analysis (PCA). To identify distinct groups of modules that share structural characteristics related to their construction material and design we conducted hierarchical cluster analyses based on Euclidean distances and Wards.D2 linkage method that were visualized using a heatmap of the standardized metric values for all artificial reef modules using functions from the ‘ggalign’ package [41]. To assess an appropriate number of module clusters, we used the silhouette method implemented with the *fviz_nbclust* function from the ‘factoextra’ package [42]. Additionally, a PCA was conducted using the *rda* function from the ‘vegan’ package [43] to summarize and visualize major patterns of variation in habitat metrics across artificial reef modules by construction material.

## Results

**Fig 4:**
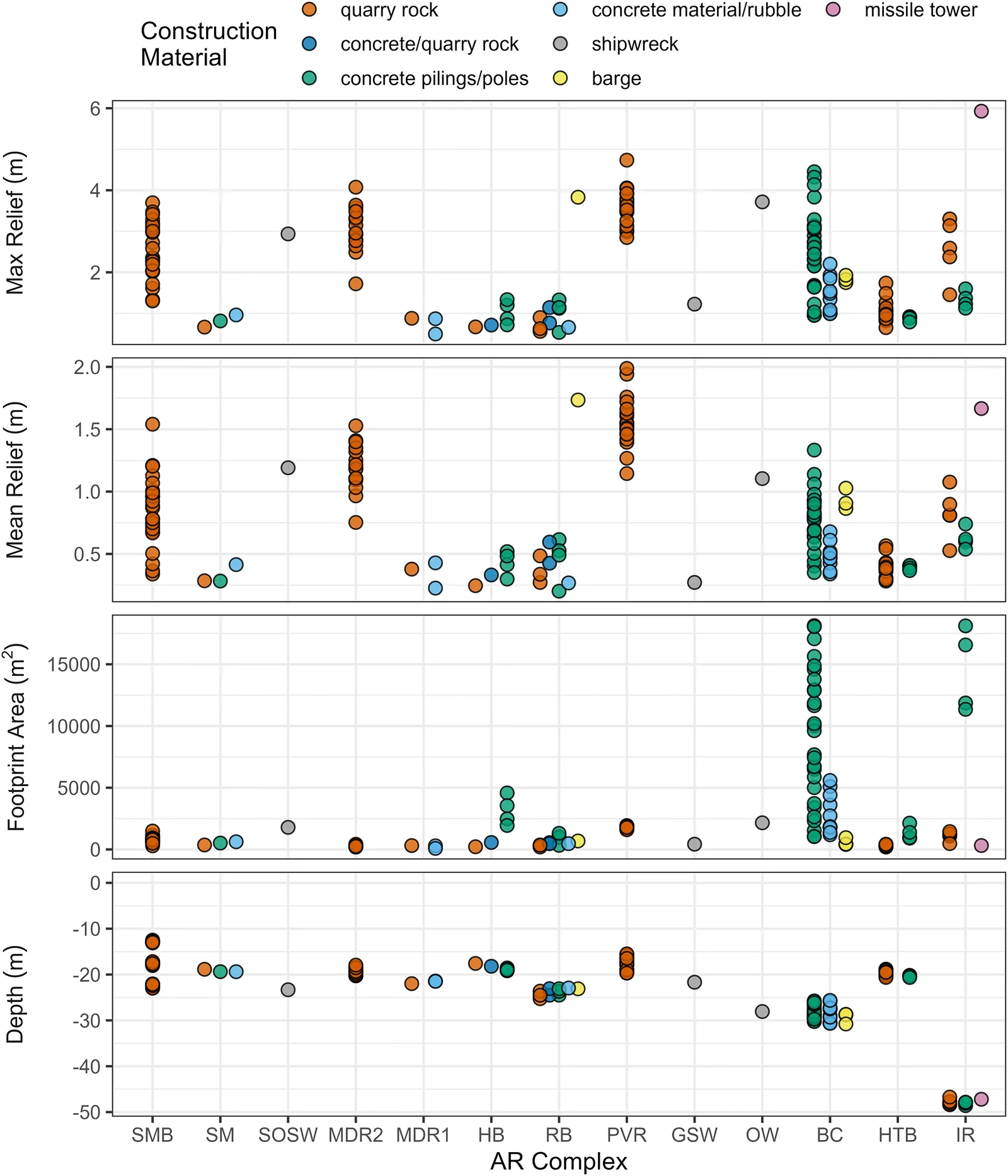
Depth and size characteristics of artificial reef modules by complex. Each point represents an individual module and colors indicate primary construction material. Panels display maximum vertical relief (m), mean vertical relief (m), footprint area (m²), and mean module depth (m). Artificial reef complexes are ordered by latitude and abbreviated as follows: SMB (Santa Monica Bay), SM (Santa Monica), SOSW (Star of Scotland Shipwreck), MDR2 (Marina Del Rey 2), MDR1 (Marina Del Rey 1), HB (Hermosa Beach), RB (Redondo Beach), PVR (Palos Verdes Reef), GSW (Georgia Straits Shipwreck), OW (Olympic Shipwreck), BC (Bolsa Chica), HTB (Huntington Beach), and IR (International Reef).

**Fig 5:**
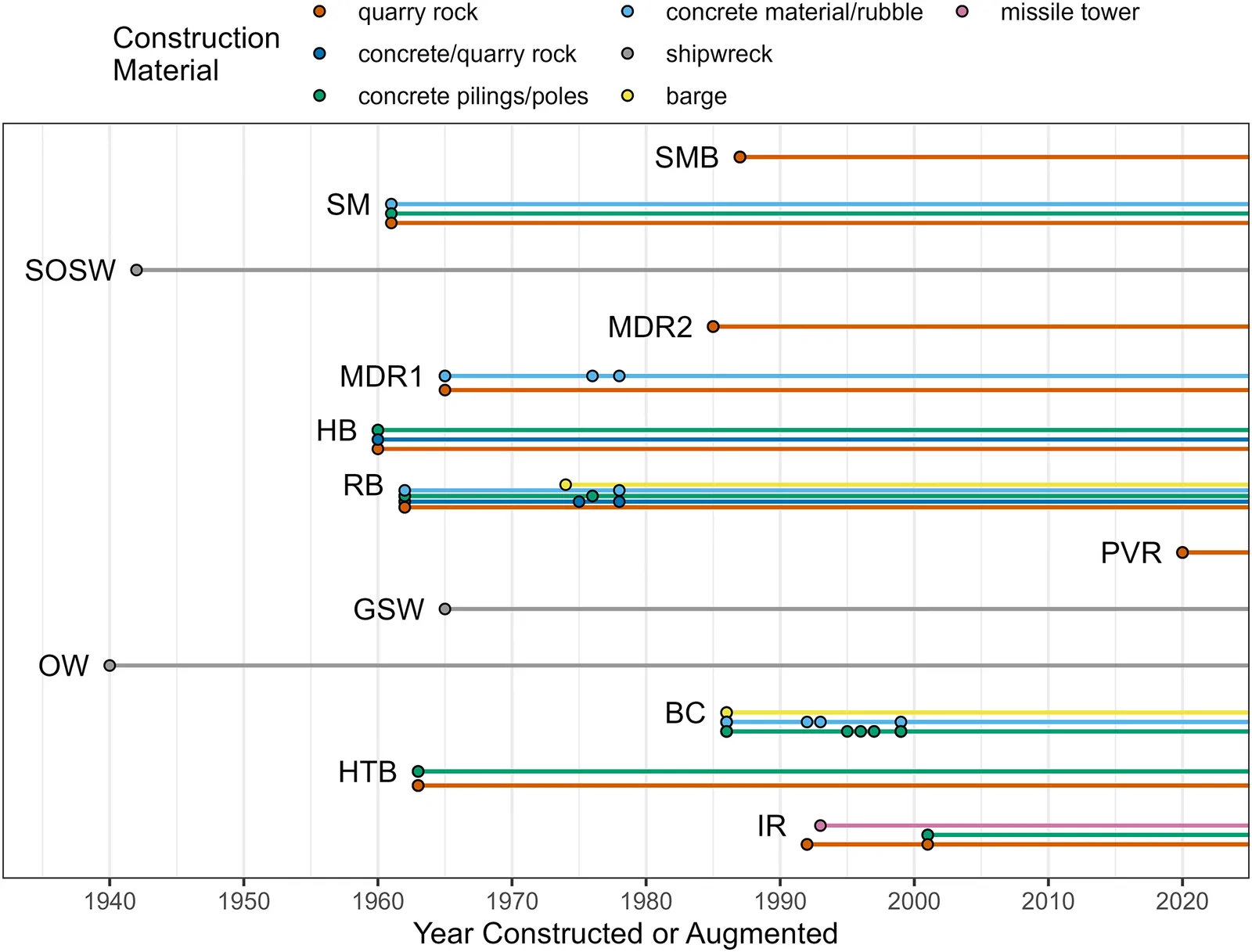
Timeline of artificial reef module construction and augmentation by complex [based on 23 and additional sources]. Labeled groups of horizontal lines represent an artificial reef complex, and points indicate the year of original construction or augmentation for individual modules, colored by primary construction material. Aartificial reef complexes are ordered by latitude and abbreviated as follows: SMB (Santa Monica Bay), SM (Santa Monica), SOSW (Star of Scotland Shipwreck), MDR2 (Marina Del Rey 2), MDR1 (Marina Del Rey 1), HB (Hermosa Beach), RB (Redondo Beach), PVR (Palos Verdes Reef), GSW (Georgia Straits Shipwreck), OW (Olympic Shipwreck), BC (Bolsa Chica), HTB (Huntington Beach), and IR (International Reef).

Across 13 artificial reef complexes and shipwrecks surveyed along the southern California coast for this study, 154 artificial reef modules were identified and named (Fig 1, Table 3, S1 Appendix) following historical sources where possible [e.g., 23]. Bolsa Chica, Olympic shipwreck and Georgia Straits shipwreck are in Federal waters, while all others are in State waters (i.e., less than three miles offshore). The number of modules within a complex varied widely, from the three shipwrecks each designated as a single artificial reef module to Bolsa Chica, which is comprised of 41 identified modules (Table 3, S1 Appendix). Across artificial reef complexes, modules composed of scattered concrete materials, including multiple adjacent Bolsa Chica modules, multiple were initially classified as a single polygon of contiguous artificial reef habitat, but then manually split along paths of soft-bottom habitat following historical naming and spatial designations [23]. Across all complexes surveyed, most modules were constructed primarily from quarry rock (n = 84) or concrete pier pilings and light poles (n = 45), with additional modules built from other types of concrete material or rubble (n = 14), concrete materials and quarry rock (n = 3), steel and concrete barges (n = 4), shipwrecks (n = 3), and a missile tower (n = 1) which is part of International Reef (Table 3, S1 Appendix). The depth range of the surveyed modules spanned from 12 m in Santa Monica Bay to 49 m at International Reef, with most modules being situated between 15 and 30 m (Fig 4, Table 3). The earliest purpose-built artificial reef complex surveyed was Hermosa Beach, constructed in 1960, with additional complexes built and augmented from the 1960s through the 1990s, and the Palos Verdes Reef constructed in 2020 representing the most recently built artificial reef complex (Fig 5, Table 3). The shipwrecks surveyed in this study sank in 1940 (Olympic), 1942 (Star of Scotland) and 1965 (Georgia Straits).

**Table 3:** Summary of artificial reef module characteristics by complex (ordered by latitude) and primary construction material category.

Artificial Reef Complex	Module Count	Constructed Year(s)	Augmented Year(s)	Depth (m)	Footprint (m²)	Mean Relief (m)	Max Relief (m)
**Santa Monica Bay (sonar survey: Apr 2023–Jul 2024)**							
quarry rock	24	1987		12–23	293–1489	0.3–1.5	1.3–3.7
**Santa Monica (sonar survey: Apr 2023–Jul 2024)**							
quarry rock	1	1961		19	363	0.3	0.7
concrete pilings/poles	1	1961		19	515	0.3	0.8
concrete material/rubble	1	1961		19	622	0.4	1
**Star of Scotland Shipwreck (sonar survey: Apr 2023)**							
shipwreck	1	1942		23	1791	1.2	2.9
**Marina Del Rey 2 (sonar survey: Jul 2023)**							
quarry rock	16	1985		18–20	196–420	0.8–1.5	1.7–4.1
**Marina Del Rey 1 (sonar survey: Jul 2023)**							
quarry rock	1	1965		22	314	0.4	0.9
concrete material/rubble	2	1965	1976, 1978	21–21	85–295	0.2–0.4	0.5–0.9
**Hermosa Beach (sonar survey: Oct 2023)**							
quarry rock	1	1960		18	217	0.2	0.7
concrete/quarry rock	1	1960		18	564	0.3	0.7
concrete pilings/poles	4	1960		19–19	1942–4578	0.3–0.5	0.7–1.3
**Redondo Beach (sonar survey: Oct 2023)**							
quarry rock	3	1962		24–25	198–362	0.3–0.5	0.6–0.9
concrete/quarry rock	2	1962	1975, 1978	23–25	465–554	0.4–0.6	0.8–1.1
concrete pilings/poles	4	1962	1976	23–24	333–1306	0.2–0.6	0.5–1.3
concrete material/rubble	1	1962	1978	23	472	0.3	0.7
barge	1	1974		23	685	1.7	3.8
**Palos Verdes Reef (sonar survey: Dec 2023)**							
quarry rock	18	2020		15–20	1596–1929	1.1–2	2.8–4.7
**Georgia Straits Shipwreck (sonar survey: Jan 2024)**							
shipwreck	1	1965		22	426	0.3	1.2
**Olympic Shipwreck (sonar survey: Jan 2024)**							
shipwreck	1	1940		28	2158	1.1	3.7
**Bolsa Chica (sonar survey: Jan 2022–Mar 2022)**							
concrete pilings/poles	28	1986	1995, 1996, 1997, 1999	26–30	1045–18130	0.4–1.3	0.9–4.5
concrete material/rubble	10	1986	1992, 1993, 1999	26–31	1187–5587	0.3–0.7	1–2.2
barge	3	1986		29–31	406–955	0.9–1	1.7–1.9
**Huntington Beach (sonar survey: Feb 2024)**							
quarry rock	15	1963		19–21	197–420	0.3–0.6	0.7–1.7
concrete pilings/poles	4	1963		20–21	917–2132	0.4–0.4	0.8–0.9
**International Reef (sonar survey: Jan 2022)**							
quarry rock	5	1992, 2001		47–48	474–1448	0.5–1.1	1.5–3.3
concrete pilings/poles	4	2001		48–49	11349–18110	0.5–0.7	1.1–1.6
missile tower	1	1993		47	317	1.7	5.9

For each artificial reef complex, the period during which sonar surveys were conducted is indicated in parentheses. Columns include the number of modules, year(s) of initial module construction, and year(s) of augmentation. For mean module depth (m), footprint area (m²), and mean and maximum vertical relief (m), values represent the range observed across modules within each material type at a given complex.

Module footprint area (m²) varied widely among module primary construction material types. The modules with the largest and most variable areas were constructed primarily from concrete pier pilings and concrete light poles (333–18,130 m², Fig 4, Table 3). The areas of quarry rock modules were more consistently moderate (196–1,929 m²), as well as modules formed by pre-existing structures such as shipwrecks, barges, and the missile tower (317–2,158 m²) (Fig 4, Table 3, S1 Appendix). The smallest was module S in Marina Del Rey 1, built from concrete rubble (85 m²; S1 Appendix), whereas the largest area was module 6a in International Reef (18,130 m²; S1 Appendix), constructed from concrete light poles; notably, this module is part of an exceptionally large area of scattered concrete light poles (S1 Appendix) that we subdivided along the larger gaps of soft bottom between reef material to result in four modules (6a–d). These were at similar areas to the larger concrete pier piling modules in Bolsa Chica (Fig 4, S1 Appendix). Modules built from concrete pilings or poles typically appeared to be scattered haphazardly across the seafloor. They included substantial patches of soft sediment among the reef material, and it was common to have individual pieces of concrete lying flat and isolated on the soft-bottom seafloor (S1 Appendix). Since, we attempted to follow historical module boundaries as described by Lewis and McKee (23), these modules are often directly adjacent to one another with reefing material sometimes separated by less than 10 m. In contrast, modules constructed from quarry rock, shipwrecks, barges, and some from concrete rubble, represent more contiguous reef material with discrete boundaries (S1 Appendix), and their footprint areas are more reflective of continuous reef extent.

Among the modules surveyed, maximum vertical relief varied from 0.5 to 5.9 m, and mean vertical relief from 0.2 to 1.7 m, with notable patterns associated with construction materials and artificial reef complex design (Fig 4). The missile tower at International Reef had the highest maximum relief (5.9 m). The Olympic shipwreck (max relief: 3.7 m) and Star of Scotland shipwreck (max relief: 2.9 m) also featured relatively high relief (Fig 4, Table 3). For quarry rock modules within a complex, Palos Verdes Reef exhibited the highest mean relief (1.1–2.0 m), and generally high maximum relief (2.8–4.7 m), while Santa Monica Bay modules were the most variable (mean relief: 0.3–1.5 m; maximum relief: 1.3–3.7 m) (Fig 4, Table 3). For concrete pier piling and light pole modules, those in Bolsa Chica displayed the highest max and mean relief, and the most variation, ranging from module 11, that is largely a single layer of concrete light poles laying horizontally on the seafloor (max relief: 0.9 m, mean relief: 0.4 m), to modules containing areas of high-relief structure of haphazardly stacked piles of concrete pier pilings, the highest being module 35 (max relief: 4.5 m, mean relief: 1.3 m) (Fig 4, Table 3, S1 Appendix). Generally, the modules primarily constructed of scattered concrete materials typically exhibited the greatest differences between maximum and mean vertical relief with the means reduced by the inclusion of relatively large patches of soft-bottom seafloor within the modules. Modules from older complexes built from a variety of materials including Santa Monica, Marina Del Rey 1, Hermosa Beach, Redondo Beach, and Huntington Beach (Fig 5) consistently had lower values of maximum and mean vertical relief (Fig 4, Table 3, S1 Appendix).

### Habitat structure characteristic patterns

**Fig 6:**
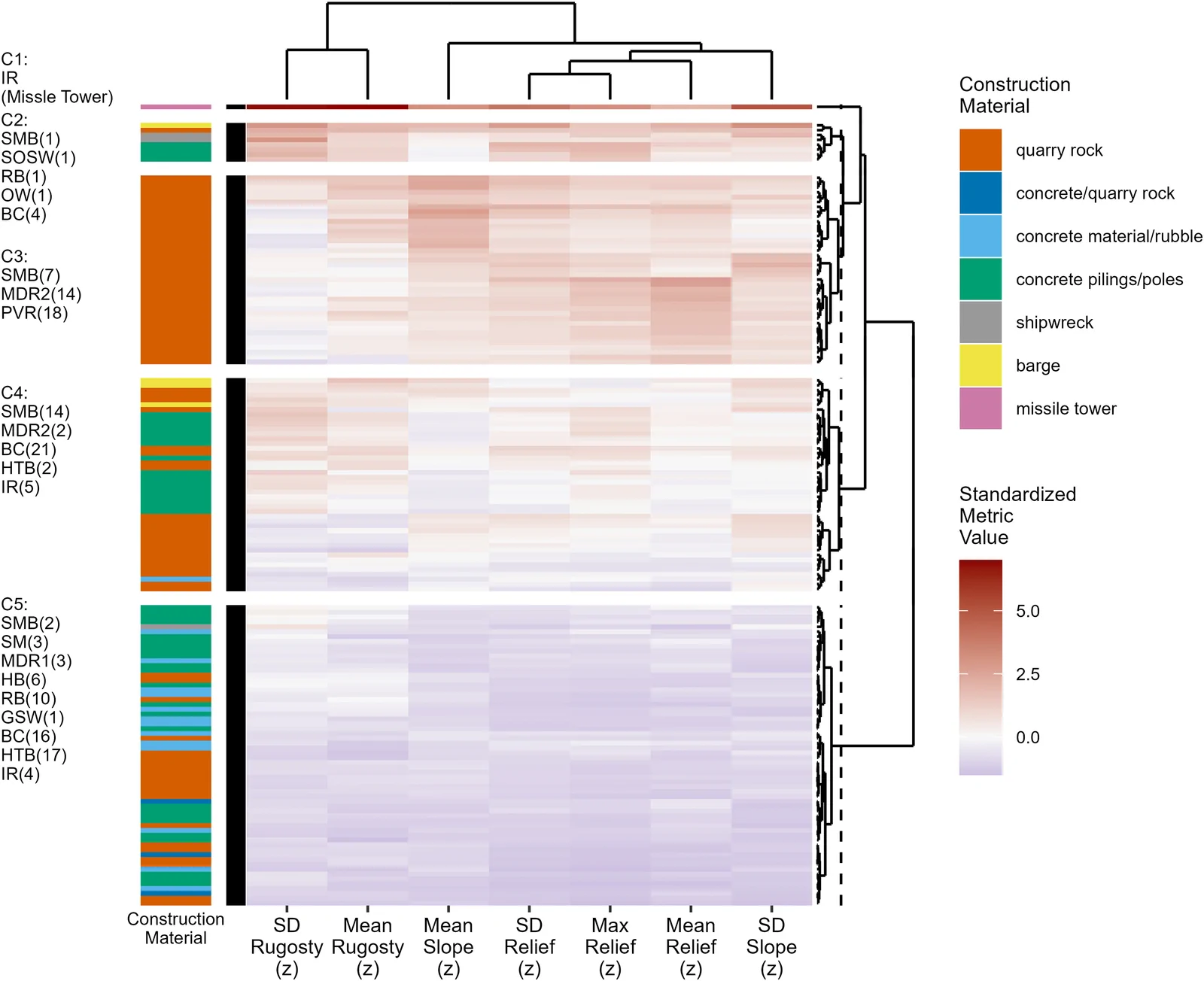
Heatmap of artificial reef modules displaying standardized (z-score) habitat metrics. Each row represents an individual artificial reef module, and each column corresponds to a metric: maximum and mean relief, mean slope, mean rugosity, and the standard deviation (sd) for each metric (quantifying within-module heterogeneity). Both rows and columns were ordered using hierarchical clustering based on Euclidean distance and the ward.D2 linkage method. Five distinct clusters of artificial reef modules (C1–C5) were identified and labeled with the corresponding artificial reef complex and the number of modules per complex in parentheses. The color bar on the left indicates modules by primary construction material type. The plot was generated using functions from the ‘ggalign’ R package [41]. Artificial reef complexes are abbreviated as follows: SMB (Santa Monica Bay), SM (Santa Monica), SOSW (Star of Scotland Shipwreck), MDR2 (Marina Del Rey 2), MDR1 (Marina Del Rey 1), HB (Hermosa Beach), RB (Redondo Beach), PVR (Palos Verdes Reef), GSW (Georgia Straits Shipwreck), OW (Olympic Shipwreck), BC (Bolsa Chica), HTB (Huntington Beach), and IR (International Reef).

**Fig 7:**
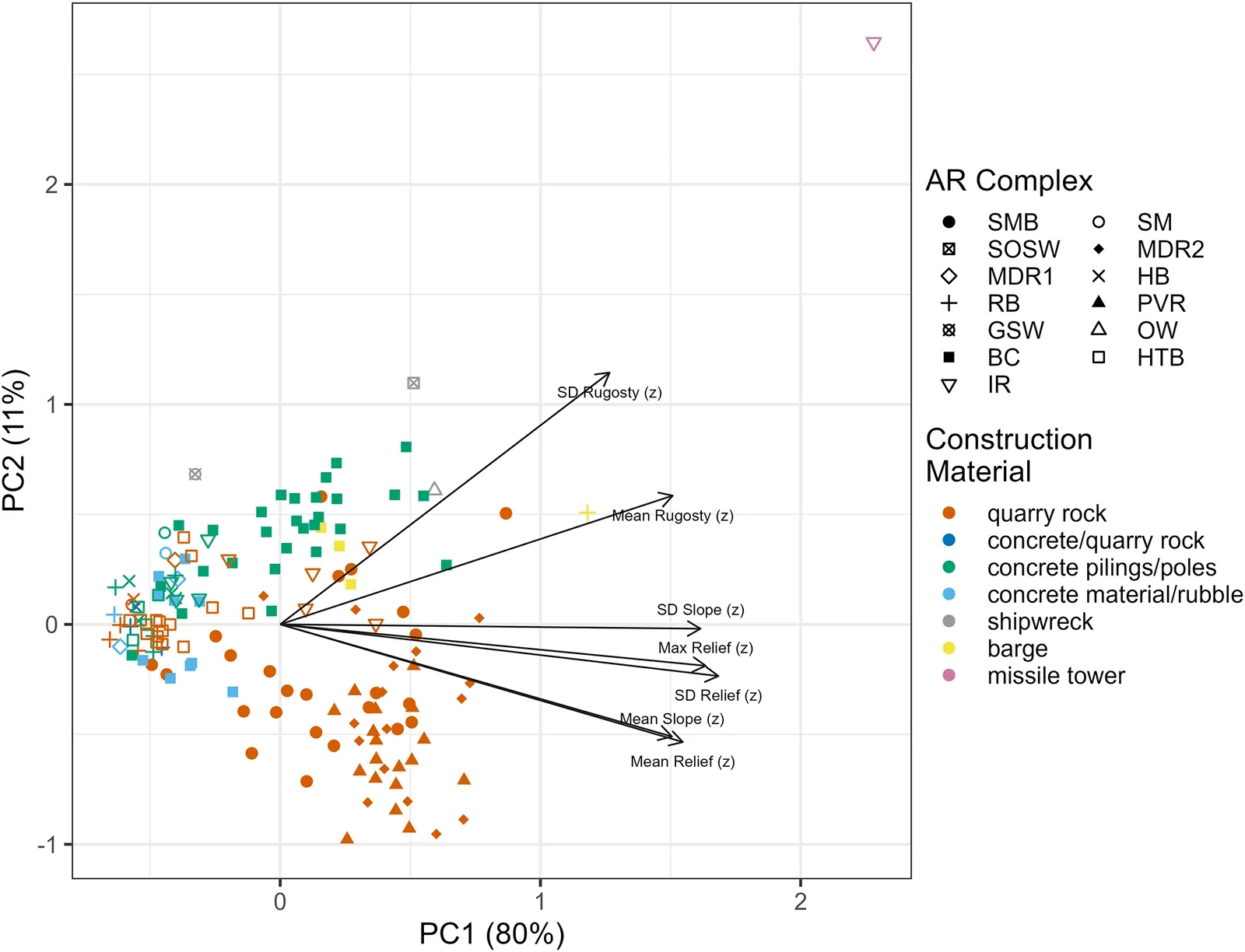
Principal components analysis (PCA) of artificial reef modules based on habitat metrics. Each point represents an individual artificial reef module, colored by primary construction material and shaped by artificial reef complex. Metrics included in the analysis were maximum and mean vertical relief, mean slope, and mean rugosity, along with heterogeneity in each of those metrics as quantified by their standard deviation (sd). All variables were centered and scaled (z-scores) prior to analysis. Arrows represent variable loadings, pointing in the direction of increasing values, with arrow length indicating the relative strength of the variable's correlation with the ordination axes. PC1 explains 80% of the total variance. PC2 explains 11% of the variance. Artificial reef complexes are abbreviated as follows: SMB (Santa Monica Bay), SM (Santa Monica), SOSW (Star of Scotland Shipwreck), MDR2 (Marina Del Rey 2), MDR1 (Marina Del Rey 1), HB (Hermosa Beach), RB (Redondo Beach), PVR (Palos Verdes Reef), GSW (Georgia Straits Shipwreck), OW (Olympic Shipwreck), BC (Bolsa Chica), HTB (Huntington Beach), and IR (International Reef).

**Fig 8:**
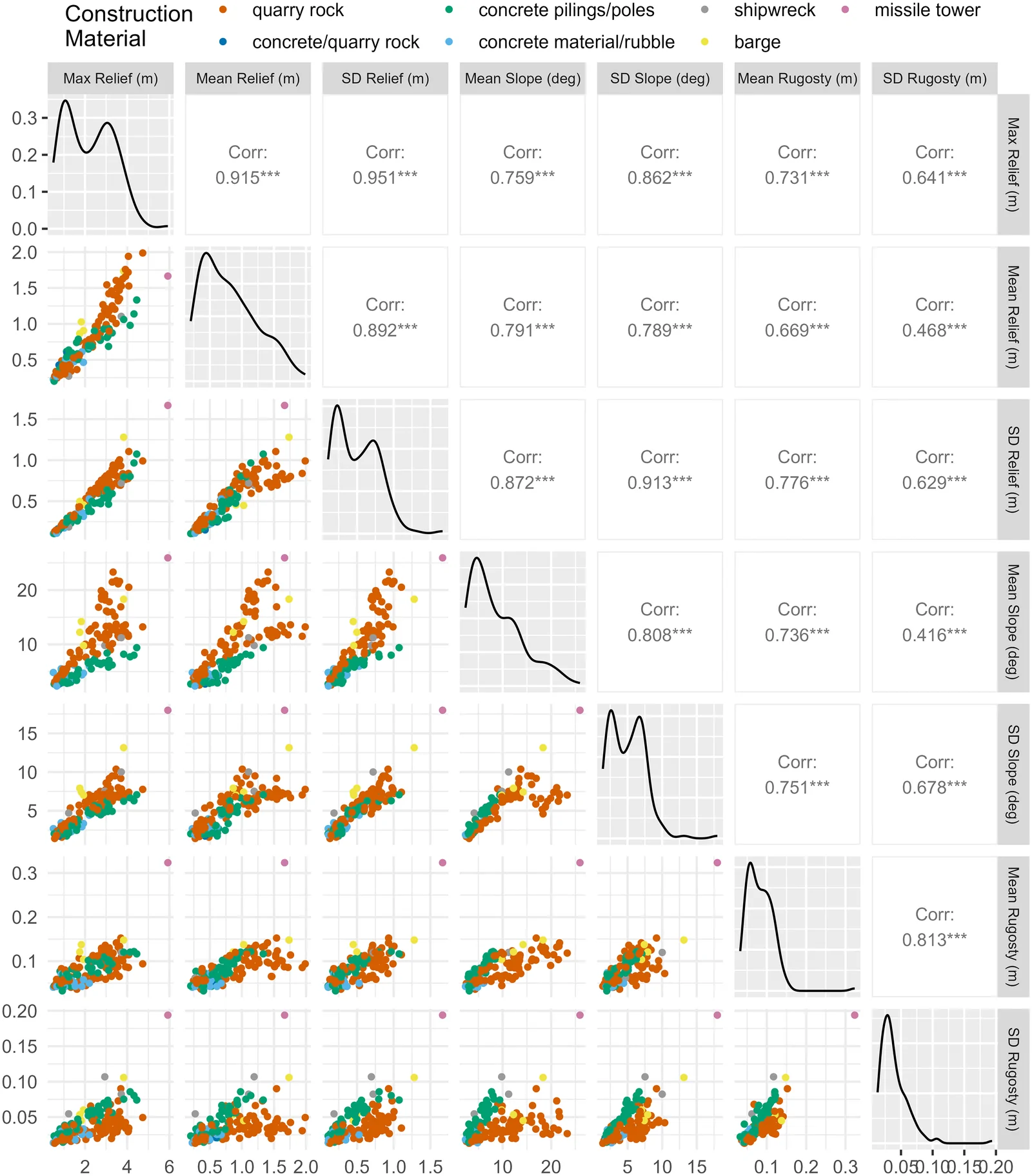
Pairwise scatterplots, density plots, and Pearson correlation coefficients among artificial reef habitat metrics. Metrics include maximum and mean vertical relief (m), mean slope (degrees), and mean rugosity (m), along with heterogeneity in each metric as quantified by their standard deviation (sd). Each point represents an individual artificial reef module, colored by primary construction material in the scatterplots. Pearson correlation coefficients (Corr.) are displayed in the upper triangle with significance levels (*p < 0.05, **p < 0.01, ***p < 0.001). Density plots along the diagonal summarize the distribution of each metric across all modules.

**Fig 9:**
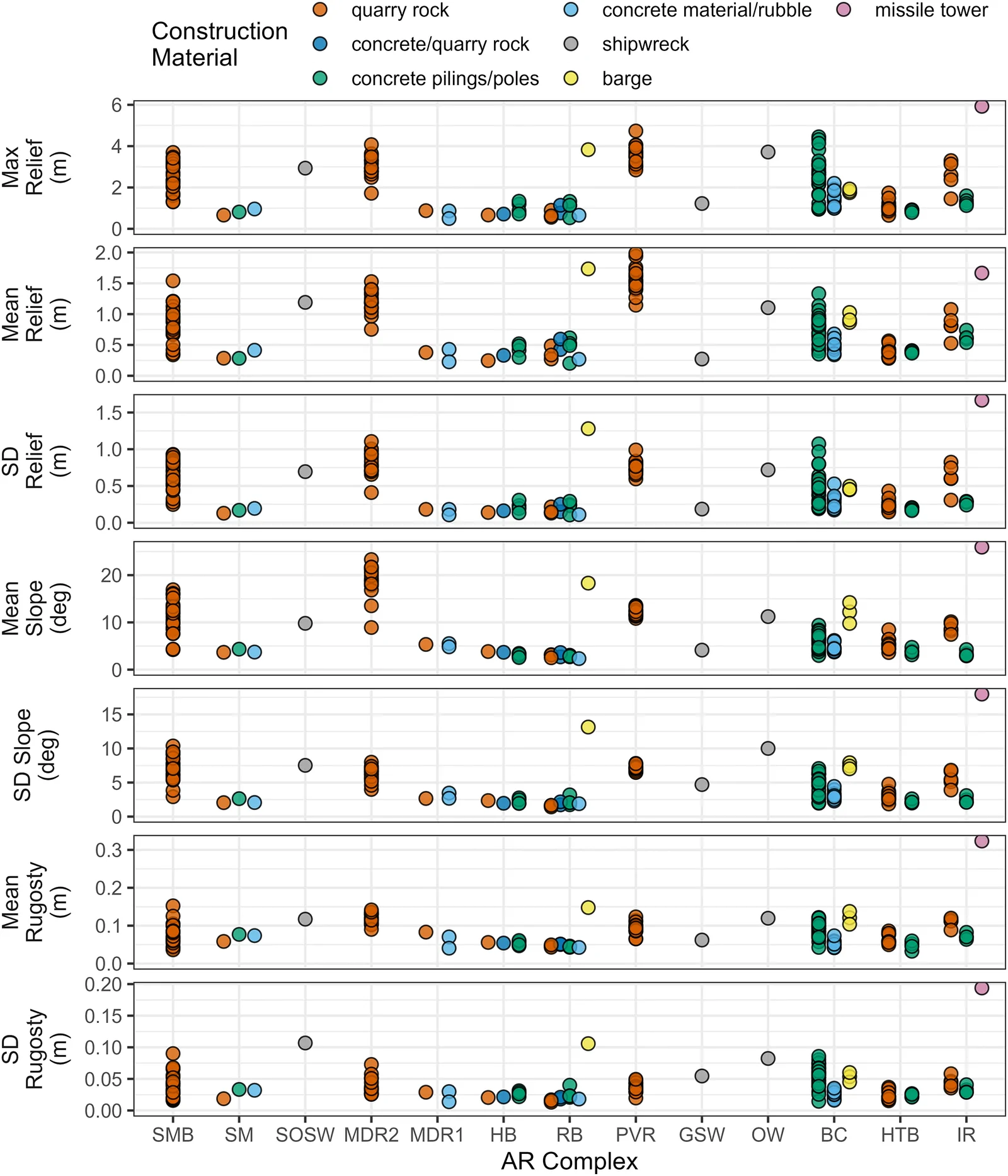
Habitat metrics of artificial reef modules by complex, with each point representing an individual module and colors indicating primary construction material. Panels display maximum vertical relief (m), mean vertical relief (m), standard deviation (sd) of relief, mean slope (degrees), sd of slope, mean rugosity (m), and sd of rugosity. Within module heterogeneity in each metric is quantified by their standard deviation. Artificial reef complexes are ordered by latitude and abbreviated as follows: SMB (Santa Monica Bay), SM (Santa Monica), SOSW (Star of Scotland Shipwreck), MDR2 (Marina Del Rey 2), MDR1 (Marina Del Rey 1), HB (Hermosa Beach), RB (Redondo Beach), PVR (Palos Verdes Reef), GSW (Georgia Straits Shipwreck), OW (Olympic Shipwreck), BC (Bolsa Chica), HTB (Huntington Beach), and IR (International Reef).

Relationships among habitat metrics revealed both redundancy and complementarity in how they quantify gradients of structural relief and complexity across artificial reef modules constructed from different materials and designs (Figs 6–8). Hierarchical clustering of the habitat metrics identified five distinct groups of modules (C1–C5) that broadly corresponded to differences in construction material and module habitat characteristics (Fig 6). The missile tower module at International Reef formed its own branch (C1) due to its exceptionally high values across most habitat metrics (Fig 9, S1 Appendix). Cluster C2 included high maximum relief and sd rugosity modules of various materials such as the Star of Scotland and Olympic shipwrecks, the Redondo Beach barge, and several of the concrete piling modules from the Bolsa Chica complex. Cluster C3 contained high relief and slope quarry rock modules, including all Palos Verdes Reef modules, most Marina del Rey 2 modules, and several of the highest-relief modules from Santa Monica Bay. Cluster C4 represented modules with moderate structural complexity and included a mixture of quarry rock, concrete piling/pole, and barge modules. Finally, cluster C5 encompassed the modules with the lowest structural complexity, characterized by relatively low mean and sd values across all metrics. These included low-relief modules constructed from concrete rubble, scattered pier pilings/poles, quarry rock, and the highly degraded hull of the Georgia Straits Shipwreck (Figs 6 and 9, S1 Appendix).

Differences among construction materials were even more evident in the PCA, where modules generally grouped by construction material and artificial reef complex (Fig 7). Across modules built from similar materials, separation occurred primarily along the first principal component (PC1), which explained 80% of the variance and represented an overall gradient from low to high relief and complexity. Modules positioned at the high end of PC1 with higher relief and steeper slopes included many quarry rock modules from Marina del Rey 2 and Palos Verdes Reef as well as the missile tower, shipwrecks, barges, and the highest relief concrete pier piling/poles modules from Bolsa Chica (Figs 7 and 9, S1 Appendix). The second principal component (PC2), explaining 11% of the variance, further separated modules by construction material, with modules composed of concrete pier pilings/poles occurring at higher PC2 values and quarry rock modules clustering toward lower PC2 values. Concrete piling/pole modules and shipwrecks tended to exhibit higher heterogeneity (sd) in rugosity, whereas quarry rock modules from Marina del Rey 2, Palos Verdes Reef, and some Santa Monica Bay modules generally had lower rugosity sd values but higher mean slope and relief (Figs 6 and 7 and 9). Modules constructed from concrete pier pilings or similar scattered materials often contained patches of relatively flat soft-bottom habitat interspersed among the hard substrate material. These features reduced mean relief and slope, but were associated with increased mean rugosity and rugosity heterogeneity (sd) likely due to the presence of extreme low values associated with flat areas and more extreme high values resulting from the abrupt transitions between hard structure and surrounding soft sediment (Figs 6 and 7 and 9, S1 Appendix). Across all modules, habitat metrics were moderately to highly correlated with one another (Pearson r = 0.41–0.95). The strongest correlations occurred among the vertical relief metrics while the rugosity metrics had the weakest correlations with the other variables (Fig 8).

## Discussion

Using high-resolution (0.3 m) sonar mapping across 13 artificial reef complexes and shipwrecks along the southern California coast, there were clear, quantifiable differences in the size and physical habitat structure among reef modules built from different construction materials and designs. Modules composed primarily of quarry rock from most complexes generally exhibited greater vertical relief, slope, and rugosity, indicating higher structural complexity, over relatively smaller, more contiguous footprints. In contrast, modules constructed from scattered concrete materials showed higher variability in habitat structure and typically exhibited lower relief and complexity but spanned larger areas that often-included substantial patches of soft-bottom habitat interspersed among the scattered reef material. Modules grouped by material and design (i.e., complex) in the multivariate analyses, indicating that this suite of bathymetry-derived habitat metrics captured complementary aspects of habitat structure and effectively quantified differences among modules. This study also provides an updated geospatial and structural characterization of several of southern California’s artificial reef complexes, improving upon the coarse positional accuracy of the existing comprehensive maps [e.g., 23] that relied on LORAN-C navigation. LORAN-C positioning typically had errors of 0.1–0.25 nautical miles [up to several hundred meters; 44], consistent with our comparisons showing that coordinates reported by Lewis and McKee [23] were often displaced by tens to hundreds of meters when plotted over the new artificial reef maps created in the present study (S1 Appendix). By combining sonar-based habitat classification, manual refinement, and quantitative terrain metrics, this work improves our ability to evaluate reef physical condition and habitat variability and provides a foundation for linking reef habitat structure to ecological function in future analyses [e.g., 11].

Sonar bathymetry data have been used extensively for decades for mapping seafloor habitat classification across a range of environments [45–47]. Building on these established methods, our workflow for mapping the extent of artificial reef modules combined multiple bathymetry-derived metrics and acoustic backscatter to classify raster cells into habitat categories and delineate reef structures from the surrounding soft sediments. The approach was adapted from established seafloor habitat classification methods [26,27,29] and incorporated complementary metrics that either enhanced the detection of reef features or reduced the influence of sonar data artifacts. Fine-scale slope helped identify small vertical-relief features associated with artificial reef material, while broader-scale VRM captured larger seascape variation and muted smaller-scale noise likely caused by surface swell that was amplified in the slope rasters [27,48]. Acoustic backscatter was useful for differentiating hard and soft substrate types and, when combined with the bathymetry-derived metrics, could indicate horizontal artificial reef construction materials just at the soft-sediment surface. We also observed instances of apparent scour around the edges of some reef structures, visible in the bathymetry metrics as localized depressions in the surrounding sediments [8,49], which sometimes corresponded to elevated backscatter values, apparently caused by coarser sediment or shell hash left after fine-grained material was removed [49]. While this integrated approach was generally effective in defining artificial reef boundaries, certain module types presented additional challenges due to their irregular or discontinuous configurations of reef material.

These challenges were most pronounced for modules constructed from scattered concrete materials, which were inherently more difficult to delineate than discrete piles of quarry rock, barge, or shipwreck modules. Modules composed of haphazardly distributed materials such as concrete pier pilings, light poles, or dock floats often lacked clear structural boundaries. This was particularly evident at the Hermosa Beach, Redondo Beach, Bolsa Chica, and International Reef complexes, where individual pieces of concrete material were frequently isolated within areas of soft sediment (S1 Appendix). Additionally, there were cases where multiple adjacent modules of scattered concrete material were initially classified as a single contiguous reef polygon but were later subdivided along soft-bottom paths to maintain historical naming and spatial designations [23]. Such modules often contained extensive patches of classified soft-bottom habitat interspersed among reef-classified cells and therefore required more manual editing to produce contiguous module polygons. There were similar challenges observed in other artificial reef mapping efforts using high-resolution sonar data [6–8]. In our case, the 0.3 m resolution of the sonar data permitted fine-scale refinement of polygon boundaries, particularly for these non–quarry rock modules through manual delineation in ArcGIS Pro. Because the mapped footprints of scattered concrete modules were more subjective and often encompassed substantial patches of interspersed soft-bottom habitat, their footprint areas should be interpreted cautiously. When using module size as an explanatory variable, it is advisable to limit analyses to modules with more contiguous designs, and in the present study those primarily were constructed from quarry rock, barges, or shipwrecks (S1 Appendix).

The habitat complexity metrics we calculated for each module, including maximum and mean vertical relief, mean slope, mean *AdjSD* rugosity, and the heterogeneity metrics based on the standard deviation of relief, slope and rugosity, build on existing sonar-based terrain metrics that have been broadly linked to marine biota [9–11,50]. Pygas, Ferrari [11] synthesized 51 studies that used remotely sensed marine seafloor habitat complexity metrics, and most were derived from bathymetry rasters at coarser resolutions (often ≥1 m). In contrast, we applied these metrics to artificial reefs using 0.3 m bathymetry to generate module-scale averages and variability measures, providing a finer scale depiction of structural features. We adopted the *AdjSD* rugosity metric developed by Ilich, Misiuk [30], which has better practical interpretability than some other rugosity metrics since it is expressed in the original measurement scale (meters) and is less correlated with slope than traditional bathymetry derived rugosity metrics, improving interpretability when also used with slope. Although we did not directly calibrate this with in situ measures [e.g., chain-and-tape rugosity; 50–52], the *AdjSD* approach offers a practical remote-sensing proxy for surface roughness, though even at 0.3 m resolution it will not capture the fine-scale detail observable with diver-based cm-scale methods. Finally, by computing standard deviation heterogeneity metrics for relief, slope, and rugosity, we moved beyond central-tendency (mean) summaries and the more traditional treatments of transect level habitat heterogeneity [e.g., 10] to quantify the distributional spread of cell values within each module. This approach may provide insight into how different species or life stages use small-scale habitat features, if for example more heterogeneous reef modules have higher species or life stage diversity as a result of fine-scale habitat preferences [3,53].

Patterns among the habitat metrics reflected how module geometry, design, and material type influenced the structural variability they captured, emphasizing their importance for ecological interpretation and metric selection in future modeling. Across modules, some of the derived habitat metrics were highly correlated, particularly the vertical relief metrics with each other, and maximum relief and heterogeneity (sd) of slope. This covariance reflects both the inherent relationships among these metrics (e.g., greater maximum vertical relief is necessary to generate greater variability in overall relief or slope) and the limited number of module designs across the reef complexes we surveyed where construction material and reef design are interrelated rather than randomized. This limited the ability to fully isolate the effects of individual metrics, a consideration for future ecological modeling. Despite these dependencies, the metrics provided interpretable measures of within-module variability aligned with known differences in reef design described previously [e.g., 23–25] and visible in the bathymetric maps (S1 Appendix). This combination of metrics separated modules across a gradient of relief and complexity and, among modules with relatively high relief and complexity, also distinguished material types such as concrete pier pilings/poles and quarry rock. However, the lowest relief and complexity modules of all material types were grouped together. This included very low average relief quarry rock modules (<1 m) and those built from scattered concrete materials with extensive patches of soft-bottom habitat (i.e., very low relief, slope, rugosity values) interspersed among hard substrates. For these scattered-concrete modules, we suggest it is probably appropriate to analyze them separately from modules composed of more contiguous materials (e.g., quarry rock, barges, shipwrecks) and to apply a distinct suite of habitat complexity metrics for their more fragmented structure. Mechanistically, piled quarry rock generate interstitial void spaces that increase with rock size [54] and create sheltering voids that are important to reef-associated taxa [4,55,56]. In contrast, scattered concrete materials often lie flat and are interspersed with soft bottom, producing lower mean relief and rugosity but elevated heterogeneity values driven by hard-soft substrate transitions, while offering fewer small-scale refuges for the associated reef community.

This dataset and workflow update Lewis and McKee [23], providing a current spatial baseline for the distribution and habitat characteristics of several southern California artificial reef complexes to guide future reef design and monitoring. They also provide a workflow for quantifying reef habitat structure that can be adapted to other natural and artificial reef mapping efforts using similar high resolution sonar data, providing the basis for future ecological analyses that link habitat features to biological responses. While remote-sensing studies have mapped artificial reefs at much larger scales [6–8], rarely has fine-scale sonar-derived habitat metrics been explicitly compared across artificial reef construction material types [e.g., 57]. Developing a comprehensive understanding of the ecological function of existing artificial reef habitats, including how quantified habitat metrics vary by construction material and design, is critical for informing future reef construction for habitat restoration [24,25,e.g., 54], compensatory mitigation [58, e.g., 59], sea-level rise adaptation [60], or other applications [1].

## Supporting information

S1 Fig Distributions of bathymetry values within Huntington Beach artificial reef modules showing the upper and lower 1.5% cutoffs (blue vertical lines) used before calculating relief metrics. The highest and lowest 1.5% of depth values within each module polygon were excluded, reducing the influence of extreme values resulting from sonar data collection artifacts, small seafloor depressions, or rare vertical protrusions while retaining the main distribution of values used to calculate module relief metrics.
(PDF)

S1 Appendix Artificial reef complex maps and module characteristics. Maps and tables for the 13 artificial reef complexes and shipwreck reefs surveyed in southern California. The appendix includes overview and site-location maps, module-level relief maps derived from sonar bathymetry, and tables of module characteristics.
(PDF)
