# Supplementary material for: High-resolution mapping of artificial reefs in southern California: Linking sonar-derived habitat metrics to reef construction material and design
Source: PLoS One. 2026 Aug 26;21(8):e0357087. doi: 10.1371/journal.pone.0357087 (PMC13514049; doi:10.1371/journal.pone.0357087)
Supplement: Supplementary file 1 [file pone.0357087.s001.pdf]

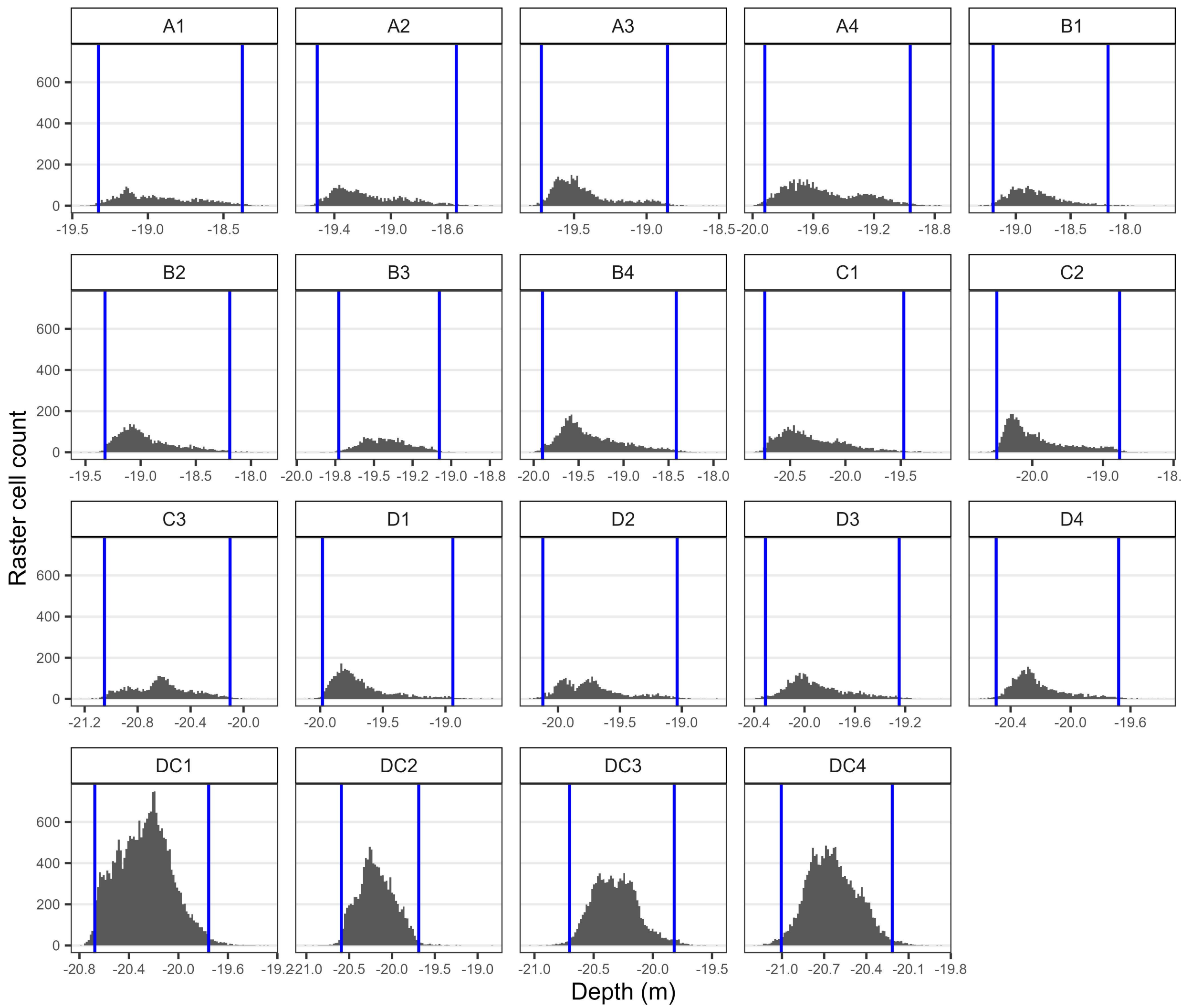

S1 Fig. Distributions of bathymetry values within Huntington Beach artificial reef modules showing the upper and lower 1.5% cutoffs (blue vertical lines) used before calculating relief metrics. The highest and lowest 1.5% of depth values within each module polygon were excluded, reducing the influence of extreme values resulting from sonar data collection artifacts, small seafloor depressions, or rare vertical protrusions while retaining the main distribution of values used to calculate module relief metrics.
