# Supplementary material for: High-resolution mapping of artificial reefs in southern California: Linking sonar-derived habitat metrics to reef construction material and design
Source: PLoS One. 2026 Aug 26;21(8):e0357087. doi: 10.1371/journal.pone.0357087 (PMC13514049; doi:10.1371/journal.pone.0357087)
Supplement: Supplementary file 2 [file pone.0357087.s002.pdf]

S1 Appendix: Artificial reef complex maps and module characteristics

Table of contents

1 Overview 2

2 Santa Monica Bay Artificial Reef 3

3 Santa Monica Artificial Reef 6

4 Star of Scotland Shipwreck 9

5 Marina Del Rey 2 Artificial Reef 12

6 Marina Del Rey 1 Artificial Reef 15

7 Hermosa Beach Artificial Reef 18

8 Redondo Beach Artificial Reef 21

9 Palos Verdes Reef 24

10 Georgia Straits Shipwreck 27

11 Olympic Shipwreck 30

12 Bolsa Chica Artificial Reef 33

13 Huntington Beach Artificial Reef 37

14 International Reef 40

1 Overview

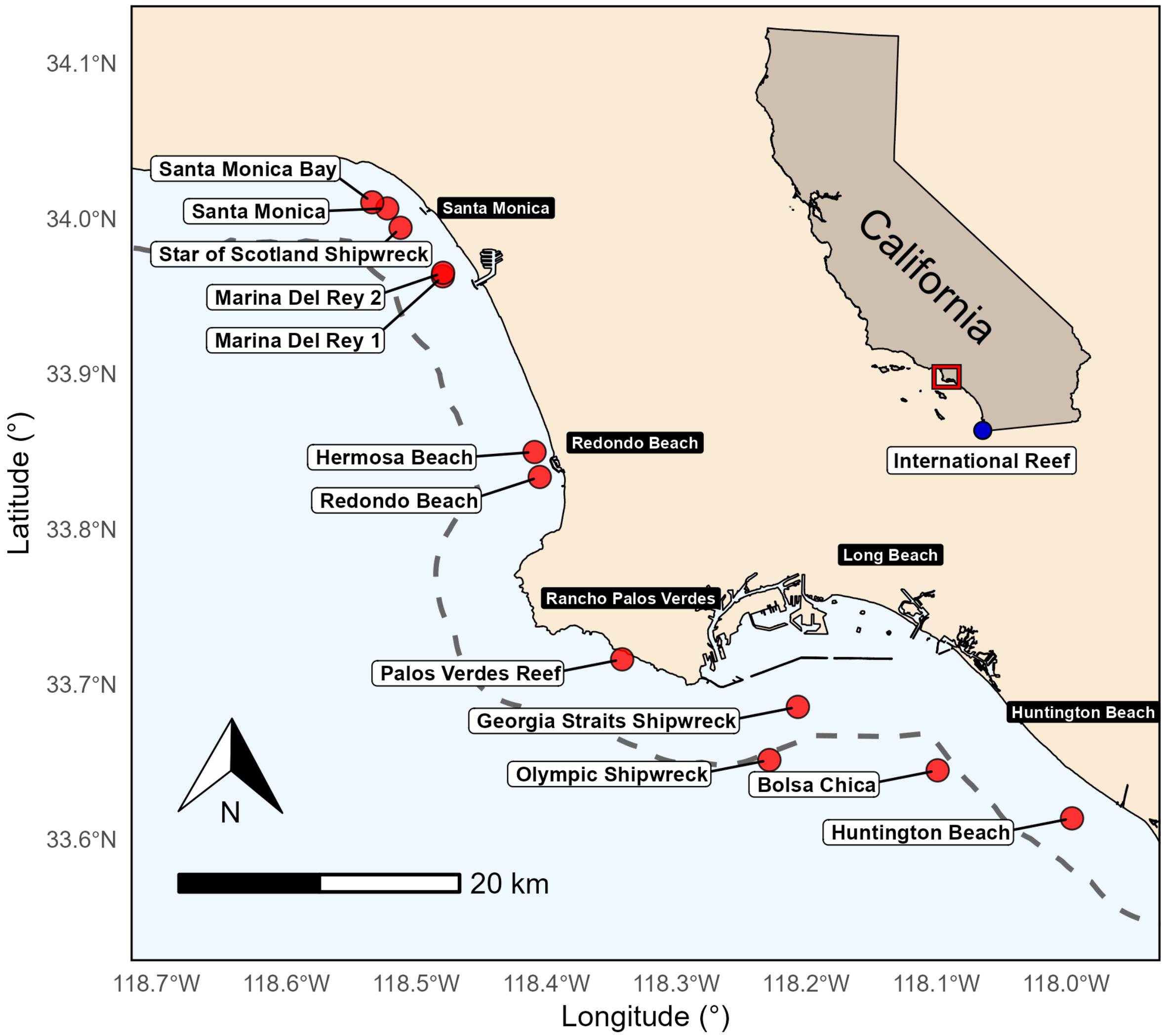

Figure A1: Artificial reef complexes and shipwreck artificial reefs in southern California that were surveyed with sonar to map the AR modules and quantify their habitat characteristics. The boundary between state and federal waters (dashed line) is generally located 3 nautical miles offshore. Note the location of the International Reef Artificial Reef Complex near United States—Mexico border is not included in the main map but is indicated by the blue circle on the California overview map.

2 Santa Monica Bay Artificial Reef

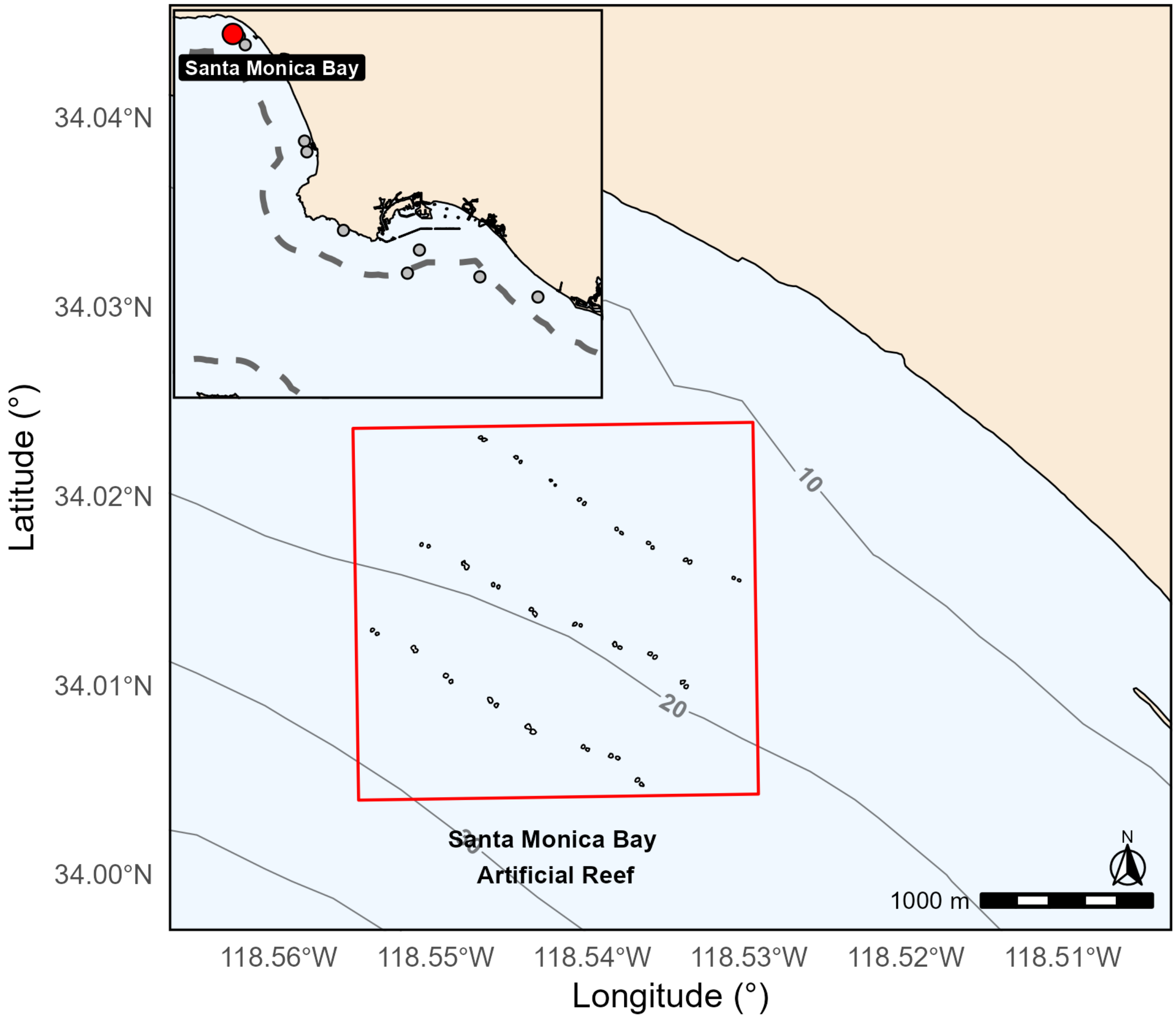

Figure A2: Location of Santa Monica Bay Artificial Reef Complex. The red bounding box indicates the area included in the AR module detail map (Figure A3). The boundary between state and federal waters (dashed line, not visible in main plot for some AR complexes) is generally located 3 nautical miles offshore. Bathymetry contours are shown in meters and were derived from the NOAA ETOPO 2022 15 Arc-Second Global Relief Model using the marmap R package.

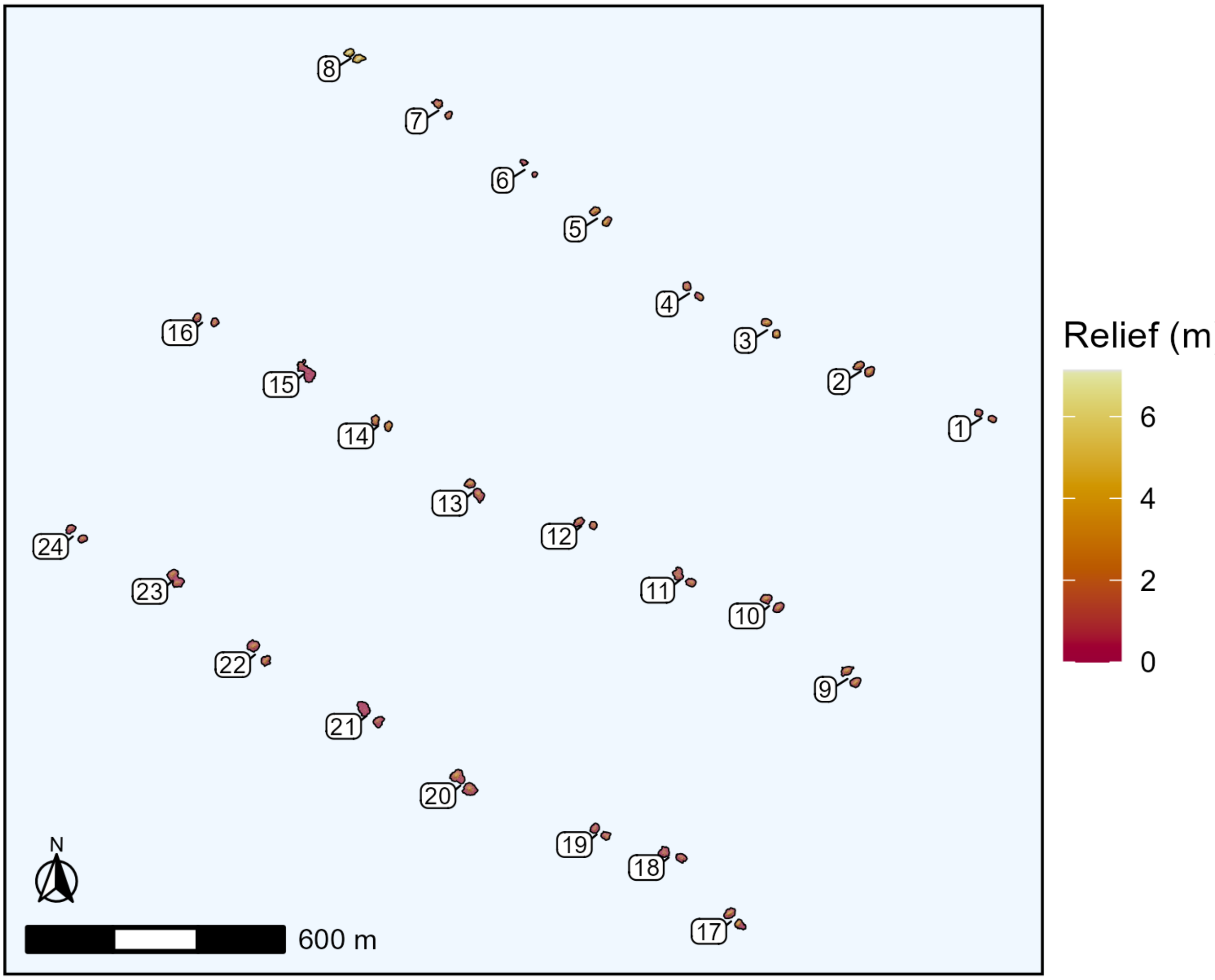

Figure A3: Map of the 24 module(s) in Santa Monica Bay Artificial Reef Complex. Sonar derived bathymetry was used to generate estimates of vertical relief in m (color).

Table A1: Characteristics of the 24 module(s) in Santa Monica Bay Artificial Reef Complex. Module naming attempted to preserve historical module spatial designations and names (Lewis and McKee 2001). The reported primary construction material was based on historical descriptions in the literature and that we confirmed from the sonar data or (where possible) visually during SCUBA or ROV surveys. Latitude and Longitude are center points of each module. Sonar derived bathymetry was used to generate estimates of average depth (m) and maximum vertical relief (m), while the 2d module footprint area was determined through a habitat classification process described in the methods. Note that for modules built from quarry rock, their areas are largely representative of the extent of contiguous material, while for most modules built from concrete material (e.g., pier pilings) these area estimates are more subjective, often inclusive of patches of soft sediment among scattered reef material. Frequently adjcent modules were originally classified as contigous reef habitat before being divided to maintain historical spatial designations and names (Lewis and McKee 2001).

Module	Material	Year Constructed (Augmented)	Latitude	Longitude	Depth (m)	Relief (m)	Foot-print (m^2)	Vol-ume (m^3)
1	quarry rock	1987	34.01560	-118.53081	13.0	2.2	493	385
10	quarry rock	1987	34.01157	-118.53614	17.2	3.2	861	792
11	quarry rock	1987	34.01209	-118.53842	17.8	2.0	869	653
12	quarry rock	1987	34.01320	-118.54095	17.5	2.0	624	416
13	quarry rock	1987	34.01386	-118.54375	17.8	3.0	933	894
14	quarry rock	1987	34.01526	-118.54615	17.2	3.0	651	789
15	quarry rock	1987	34.01632	-118.54808	18.0	1.3	1,079	365
16	quarry rock	1987	34.01739	-118.55067	17.3	2.3	560	438
17	quarry rock	1987	34.00488	-118.53699	22.2	3.5	875	879
18	quarry rock	1987	34.00621	-118.53861	22.0	1.7	849	428
19	quarry rock	1987	34.00667	-118.54044	22.4	2.0	672	470
2	quarry rock	1987	34.01656	-118.53389	12.9	3.4	775	931
20	quarry rock	1987	34.00767	-118.54393	23.0	3.2	1,489	1,321
21	quarry rock	1987	34.00910	-118.54635	23.0	1.6	1,155	420
22	quarry rock	1987	34.01037	-118.54920	22.5	2.7	944	819
23	quarry rock	1987	34.01190	-118.55133	22.1	2.4	953	686
24	quarry rock	1987	34.01282	-118.55387	22.1	2.4	629	426
3	quarry rock	1987	34.01740	-118.53629	12.8	3.3	605	647
4	quarry rock	1987	34.01815	-118.53829	13.1	2.6	578	556
5	quarry rock	1987	34.01971	-118.54066	12.7	3.1	672	757
6	quarry rock	1987	34.02072	-118.54252	13.0	1.3	293	123
7	quarry rock	1987	34.02194	-118.54473	13.0	2.3	549	408
8	quarry rock	1987	34.02302	-118.54698	12.5	3.7	717	1,102
9	quarry rock	1987	34.01004	-118.53412	17.6	3.0	849	840

3 Santa Monica Artificial Reef

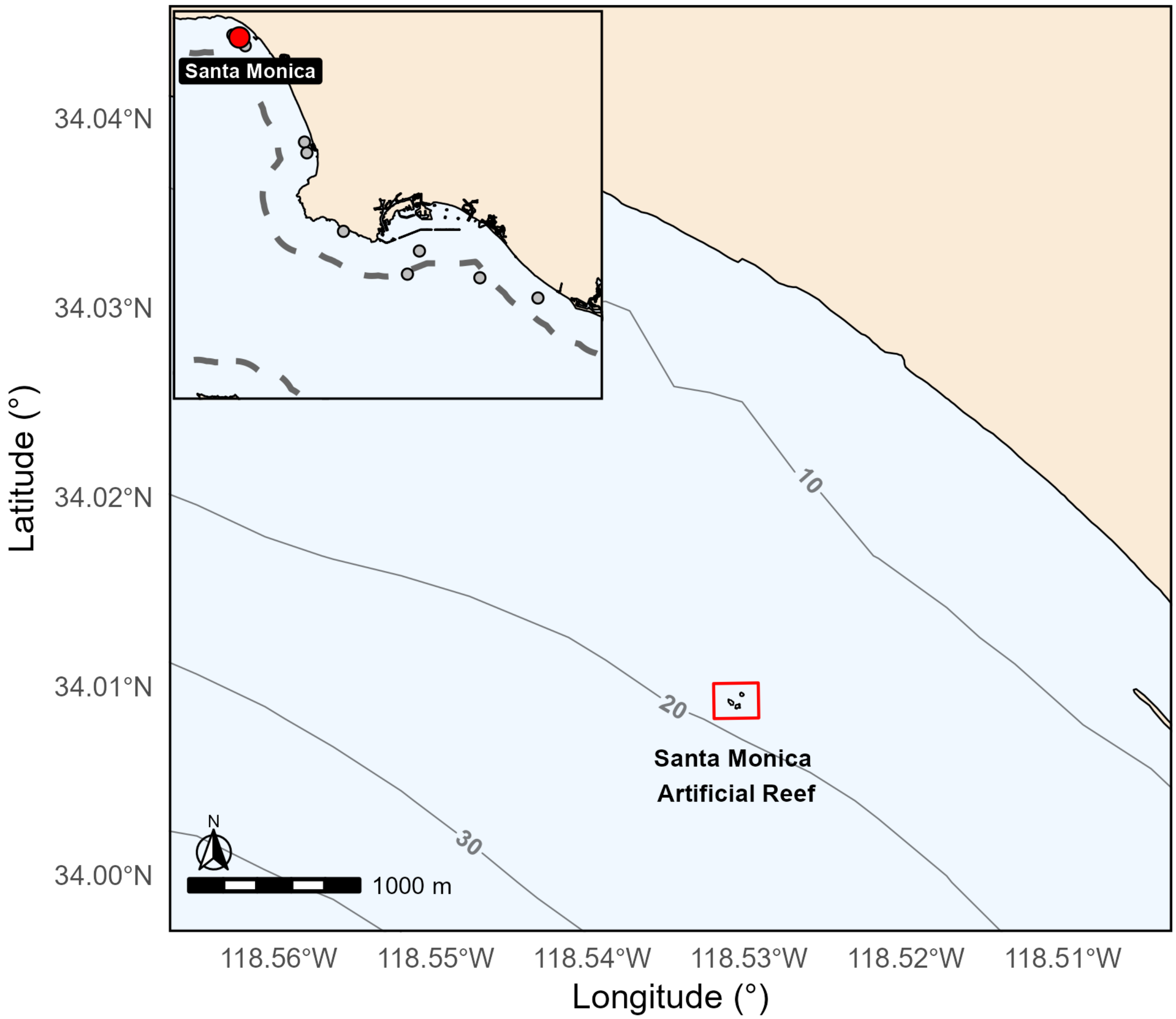

Figure A4: Location of Santa Monica Artificial Reef Complex. The red bounding box indicates the area included in the AR module detail map (Figure A5). The boundary between state and federal waters (dashed line, not visible in main plot for some AR complexes) is generally located 3 nautical miles offshore. Bathymetry contours are shown in meters and were derived from the NOAA ETOPO 2022 15 Arc-Second Global Relief Model using the marmap R package.

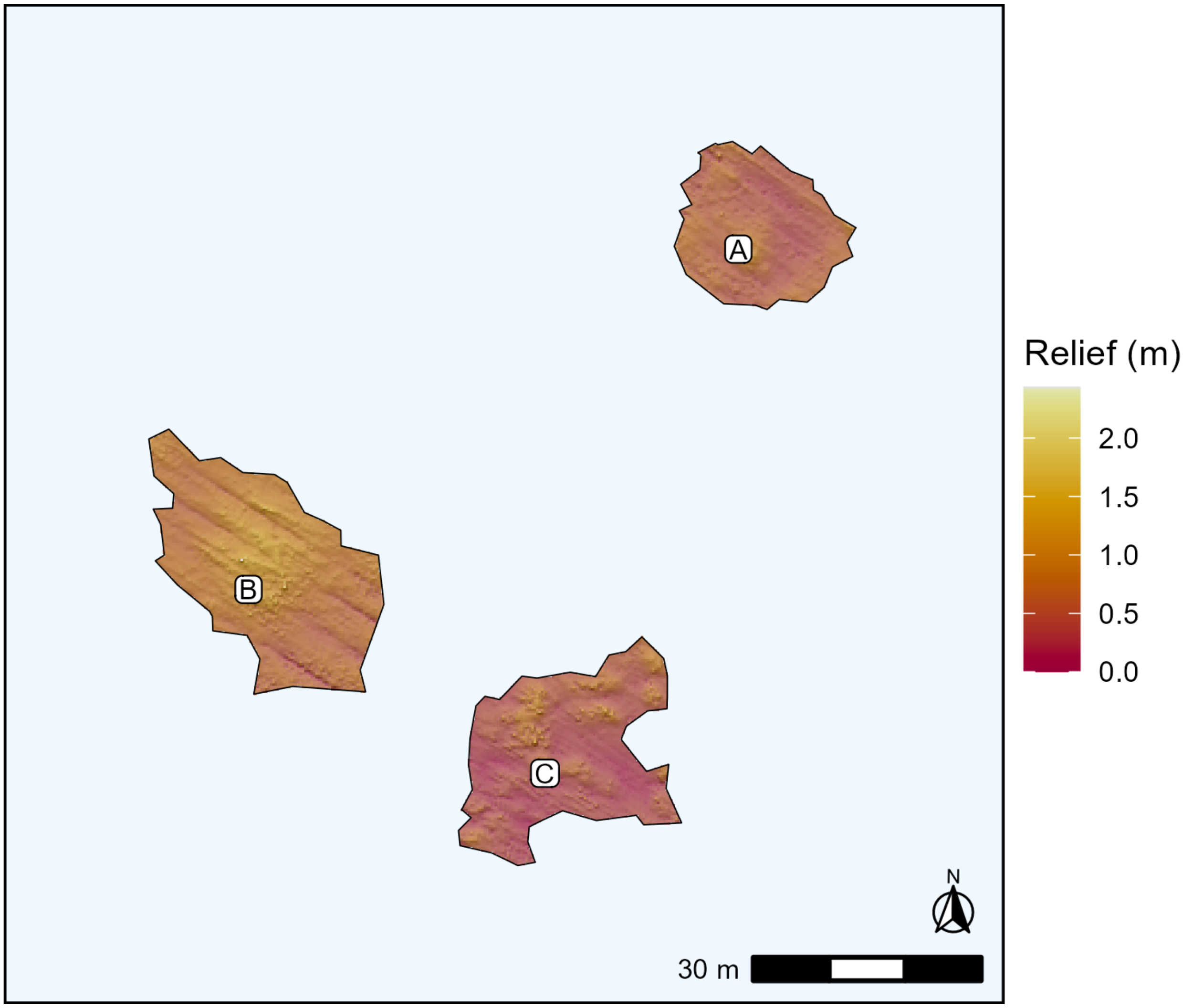

Figure A5: Map of the 3 module(s) in Santa Monica Artificial Reef Complex. Sonar derived bathymetry was used to generate estimates of vertical relief in m (color).

Table A2: Characteristics of the 3 module(s) in Santa Monica Artificial Reef Complex. Module naming attempted to preserve historical module spatial designations and names (Lewis and McKee 2001). The reported primary construction material was based on historical descriptions in the literature and that we confirmed from the sonar data or (where possible) visually during SCUBA or ROV surveys. Latitude and Longitude are center points of each module. Sonar derived bathymetry was used to generate estimates of average depth (m) and maximum vertical relief (m), while the 2d module footprint area was determined through a habitat classification process described in the methods. Note that for modules built from quarry rock, their areas are largely representative of the extent of contiguous material, while for most modules built from concrete material (e.g., pier pilings) these area estimates are more subjective, often inclusive of patches of soft sediment among scattered reef material. Frequently adjcent modules were originally classified as contigous reef habitat before being divided to maintain historical spatial designations and names (Lewis and McKee 2001).

Module	Material	Year Constructed (Augmented)	Latitude	Longitude	Depth (m)	Relief (m)	Footprint (m^2)	Volume (m^3)
A	quarry rock	1961	34.00956	-118.53046	18.9	0.7	363	103
B	concrete shelters; quarry rock	1961	34.00914	-118.53115	19.4	1.0	622	258
C	streetcar; concrete pilings	1961	34.00893	-118.53072	19.4	0.8	515	146

4 Star of Scotland Shipwreck

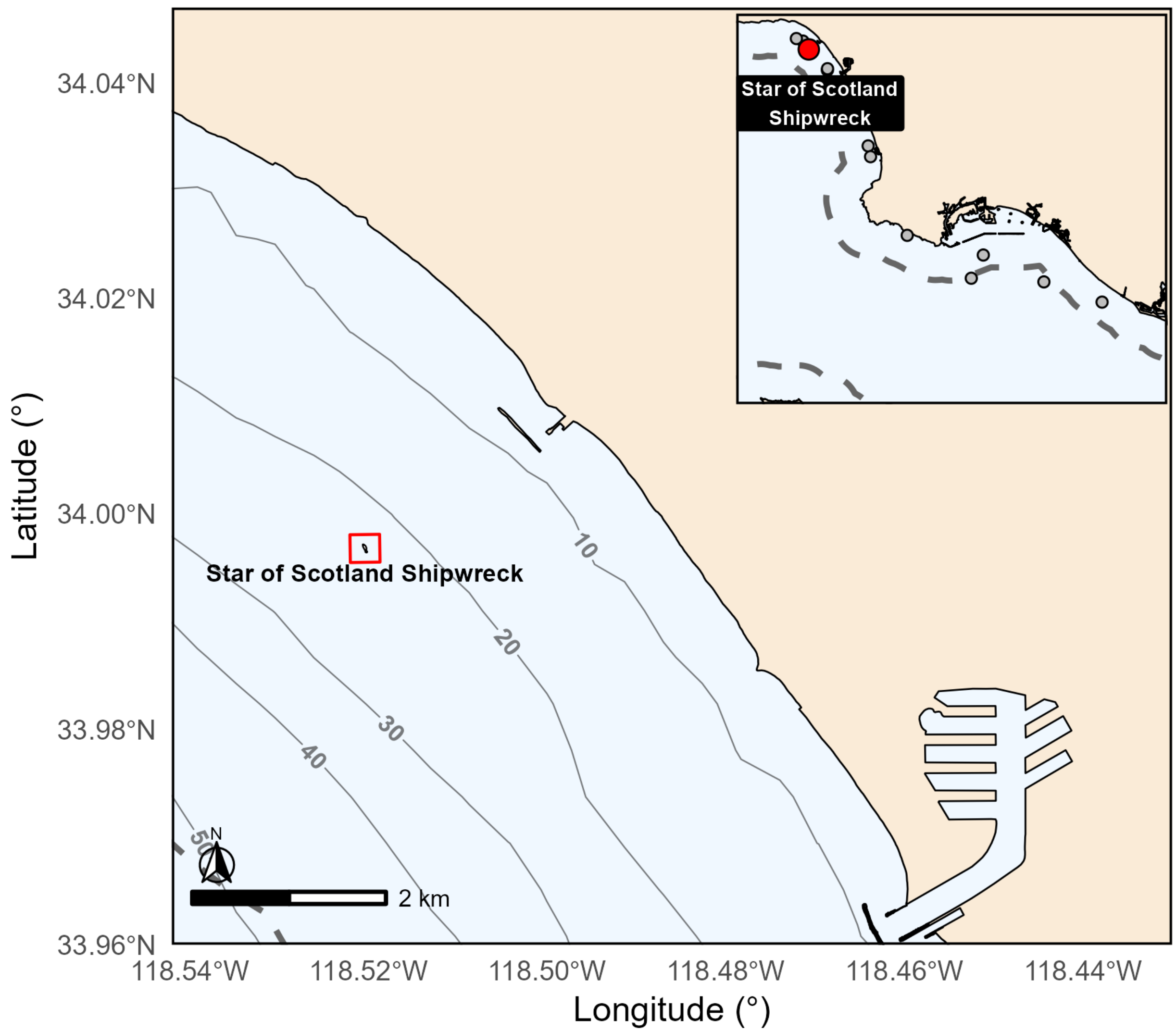

Figure A6: Location of Star of Scotland Shipwreck. The red bounding box indicates the area included in the AR module detail map (Figure A7). The boundary between state and federal waters (dashed line, not visible in main plot for some AR complexes) is generally located 3 nautical miles offshore. Bathymetry contours are shown in meters and were derived from the NOAA ETOPO 2022 15 Arc-Second Global Relief Model using the marmap R package.

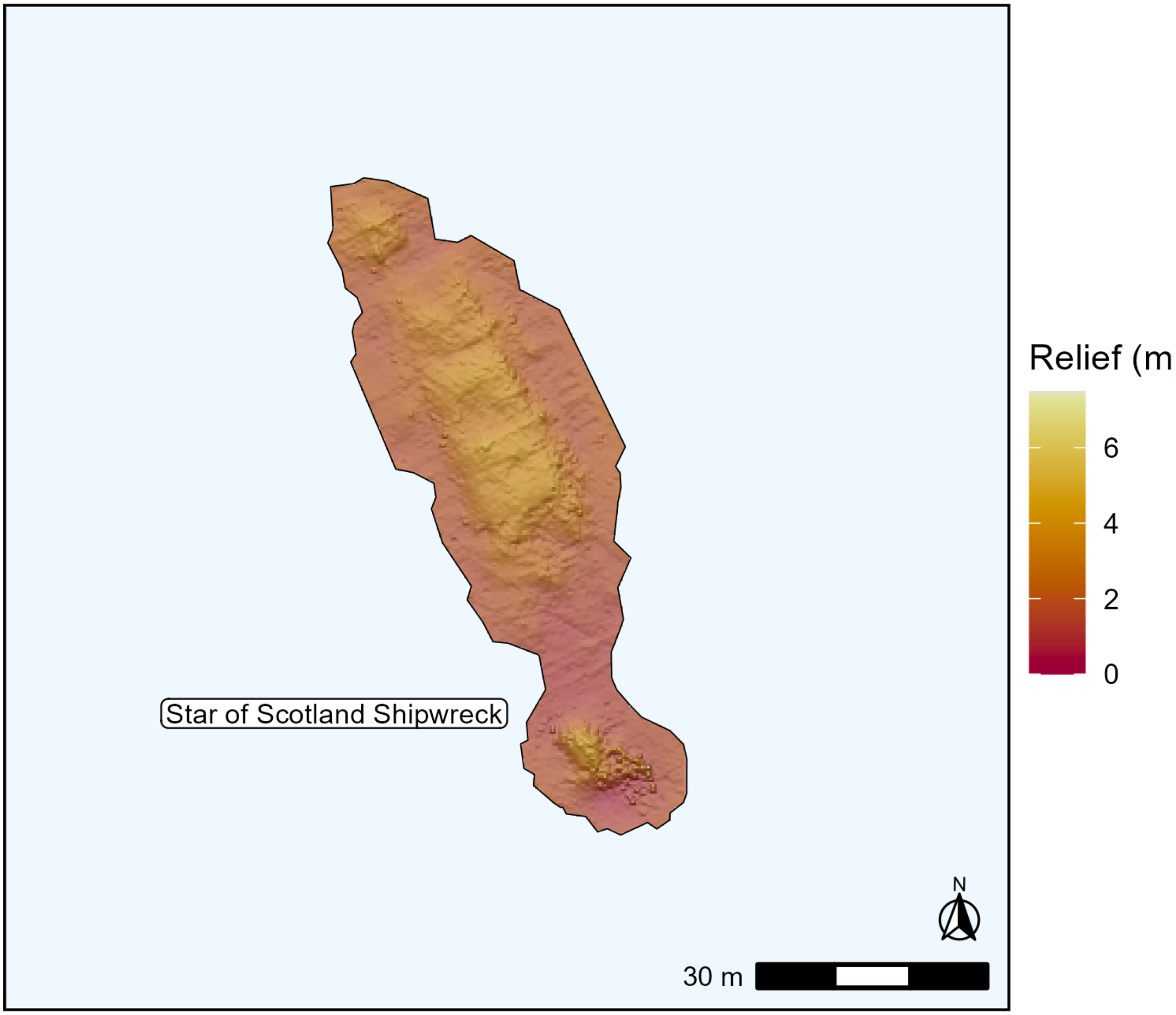

Figure A7: Map of the 1 module(s) in Star of Scotland Shipwreck. Sonar derived bathymetry was used to generate estimates of vertical relief in m (color).

Table A3: Characteristics of the 1 module(s) in Star of Scotland Shipwreck. Module naming attempted to preserve historical module spatial designations and names (Lewis and McKee 2001). The reported primary construction material was based on historical descriptions in the literature and that we confirmed from the sonar data or (where possible) visually during SCUBA or ROV surveys. Latitude and Longitude are center points of each module. Sonar derived bathymetry was used to generate estimates of average depth (m) and maximum vertical relief (m), while the 2d module footprint area was determined through a habitat classification process described in the methods. Note that for modules built from quarry rock, their areas are largely representative of the extent of contiguous material, while for most modules built from concrete material (e.g., pier pilings) these area estimates are more subjective, often inclusive of patches of soft sediment among scattered reef material. Frequently adjcent modules were originally classified as contigous reef habitat before being divided to maintain historical spatial designations and names (Lewis and McKee 2001).

Module	Material	Year Constructed (Augmented)	Latitude	Longitude	Depth (m)	Relief (m)	Foot-print (m^2)	Vol-ume (m^3)
Star of Scotland	shipwreck	1942	33.99682	-118.52037	23.3	2.9	1,791	2,133

5 Marina Del Rey 2 Artificial Reef

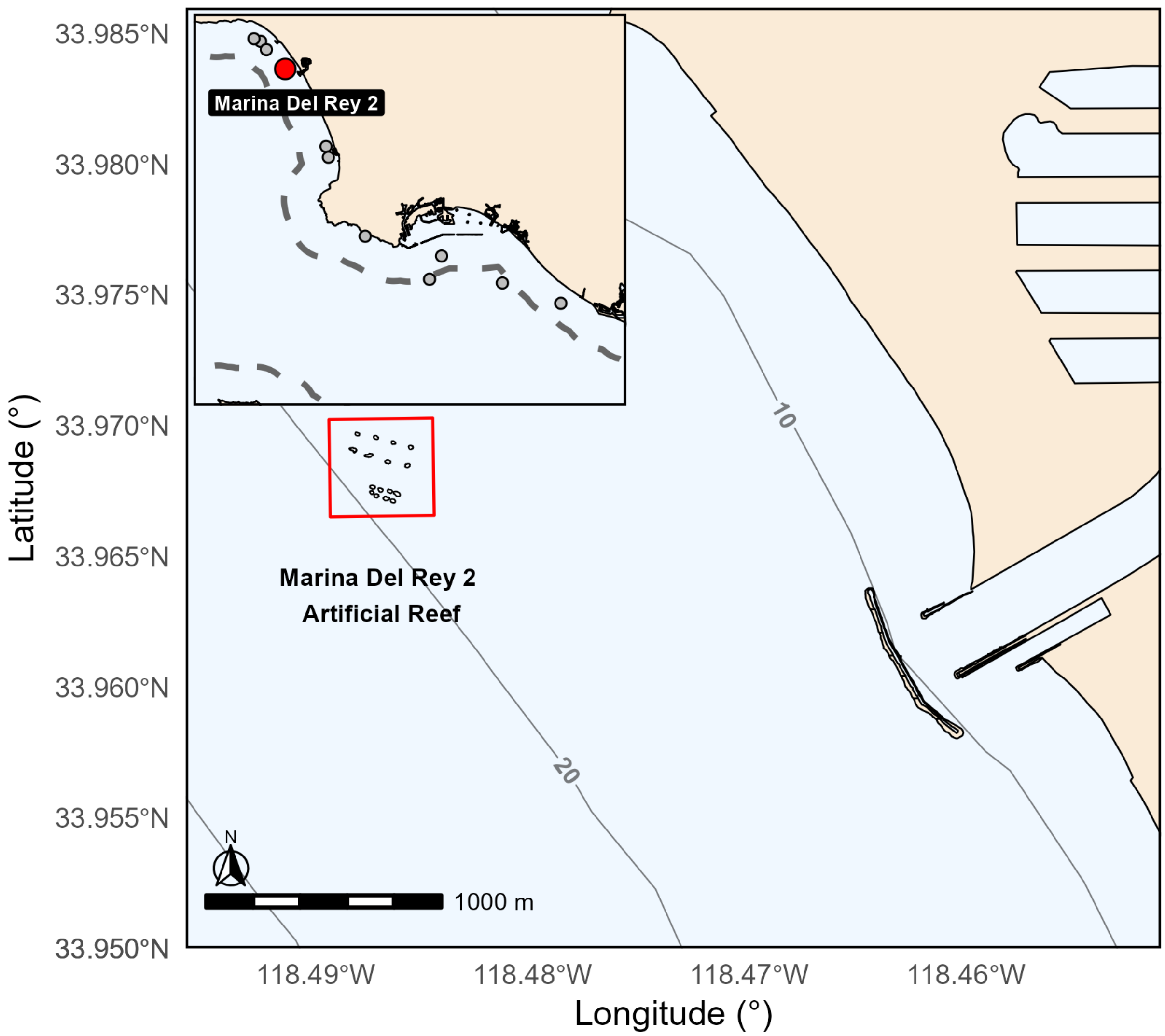

Figure A8: Location of Marina Del Rey 2 Artificial Reef Complex. The red bounding box indicates the area included in the AR module detail map (Figure A9). The boundary between state and federal waters (dashed line, not visible in main plot for some AR complexes) is generally located 3 nautical miles offshore. Bathymetry contours are shown in meters and were derived from the NOAA ETOPO 2022 15 Arc-Second Global Relief Model using the marmap R package.

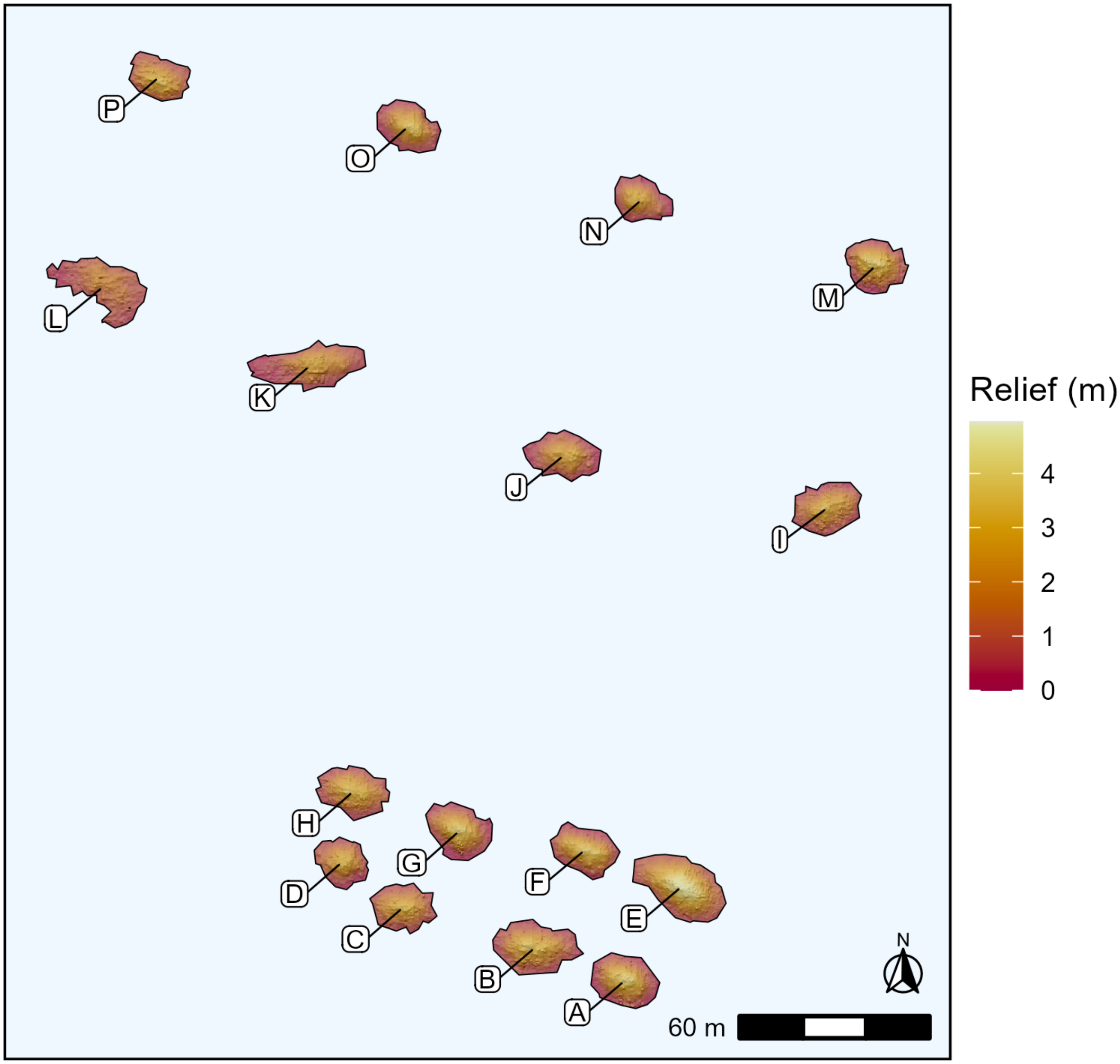

Figure A9: Map of the 16 module(s) in Marina Del Rey 2 Artificial Reef Complex. Sonar derived bathymetry was used to generate estimates of vertical relief in m (color).

Table A4: Characteristics of the 16 module(s) in Marina Del Rey 2 Artificial Reef Complex. Module naming attempted to preserve historical module spatial designations and names (Lewis and McKee 2001). The reported primary construction material was based on historical descriptions in the literature and that we confirmed from the sonar data or (where possible) visually during SCUBA or ROV surveys. Latitude and Longitude are center points of each module. Sonar derived bathymetry was used to generate estimates of average depth (m) and maximum vertical relief (m), while the 2d module footprint area was determined through a habitat classification process described in the methods. Note that for modules built from quarry rock, their areas are largely representative of the extent of contiguous material, while for most modules built from concrete material (e.g., pier pilings) these area estimates are more subjective, often inclusive of patches of soft sediment among scattered reef material. Frequently adjcent modules were originally classified as contigous reef habitat before being divided to maintain historical spatial designations and names (Lewis and McKee 2001).

Module	Material	Year Constructed (Augmented)	Latitude	Longitude	Depth (m)	Relief (m)	Footprint (m^2)	Volume (m^3)
A	quarry rock	1985	33.96712	-118.48644	19.6	3.6	277	374
B	quarry rock	1985	33.96721	-118.48675	20.0	3.1	339	399
C	quarry rock	1985	33.96732	-118.48720	20.2	2.8	232	253
D	quarry rock	1985	33.96745	-118.48741	20.3	2.9	196	233
E	quarry rock	1985	33.96739	-118.48625	19.3	4.1	420	642
F	quarry rock	1985	33.96749	-118.48658	19.4	3.3	249	351
G	quarry rock	1985	33.96754	-118.48701	19.9	3.6	272	359
H	quarry rock	1985	33.96765	-118.48738	20.1	2.9	271	323
I	quarry rock	1985	33.96848	-118.48577	18.5	3.0	275	335
J	quarry rock	1985	33.96862	-118.48667	19.2	2.6	265	274
K	quarry rock	1985	33.96886	-118.48755	19.7	2.5	359	345
L	quarry rock	1985	33.96908	-118.48826	20.3	1.7	363	273
M	quarry rock	1985	33.96917	-118.48561	17.9	3.5	250	350
N	quarry rock	1985	33.96935	-118.48642	18.4	2.8	198	219
O	quarry rock	1985	33.96955	-118.48722	18.7	3.3	241	302
P	quarry rock	1985	33.96968	-118.48808	19.3	2.8	210	232

6 Marina Del Rey 1 Artificial Reef

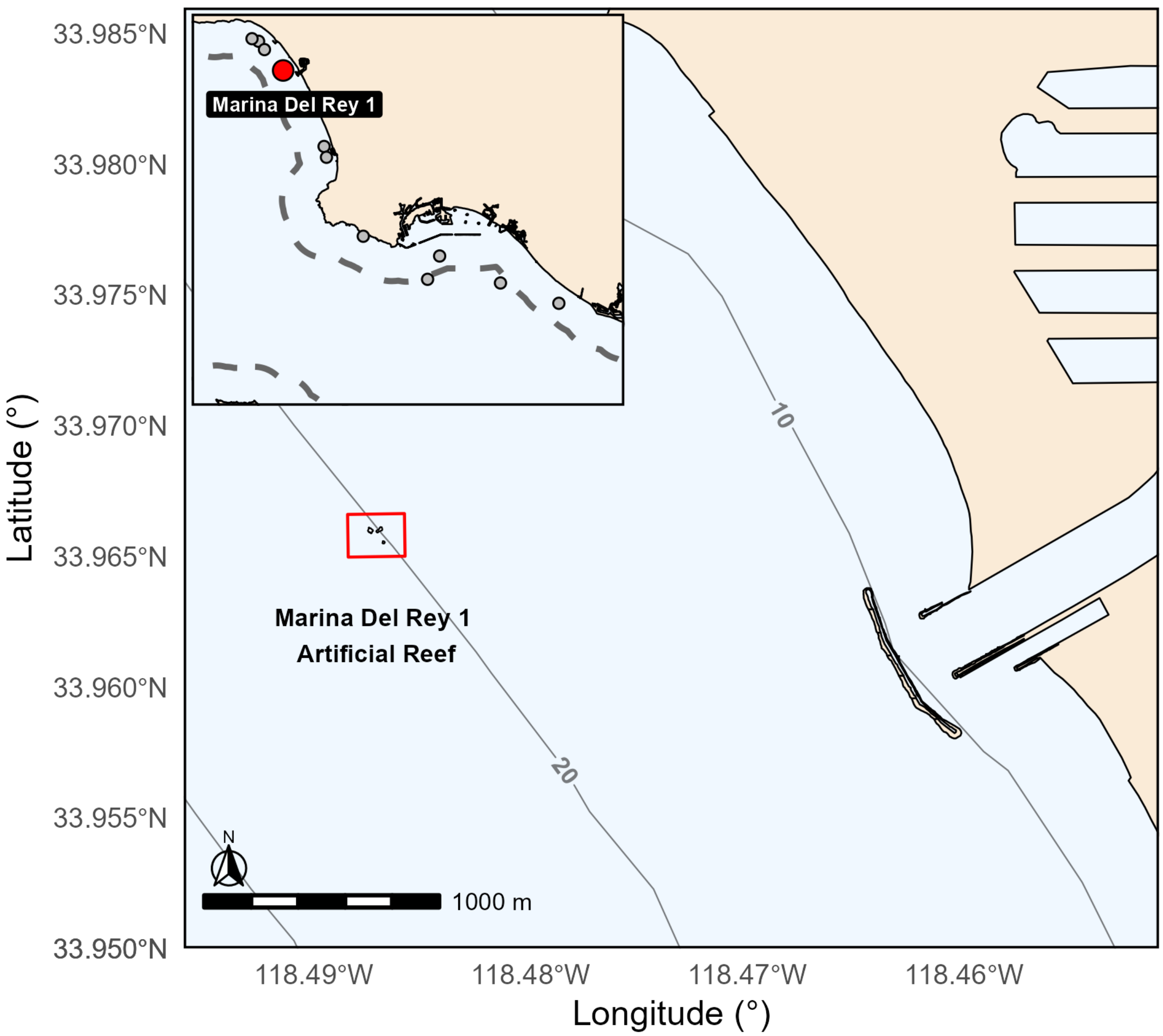

Figure A10: Location of Marina Del Rey 1 Artificial Reef Complex. The red bounding box indicates the area included in the AR module detail map (Figure A11). The boundary between state and federal waters (dashed line, not visible in main plot for some AR complexes) is generally located 3 nautical miles offshore. Bathymetry contours are shown in meters and were derived from the NOAA ETOPO 2022 15 Arc-Second Global Relief Model using the marmap R package.

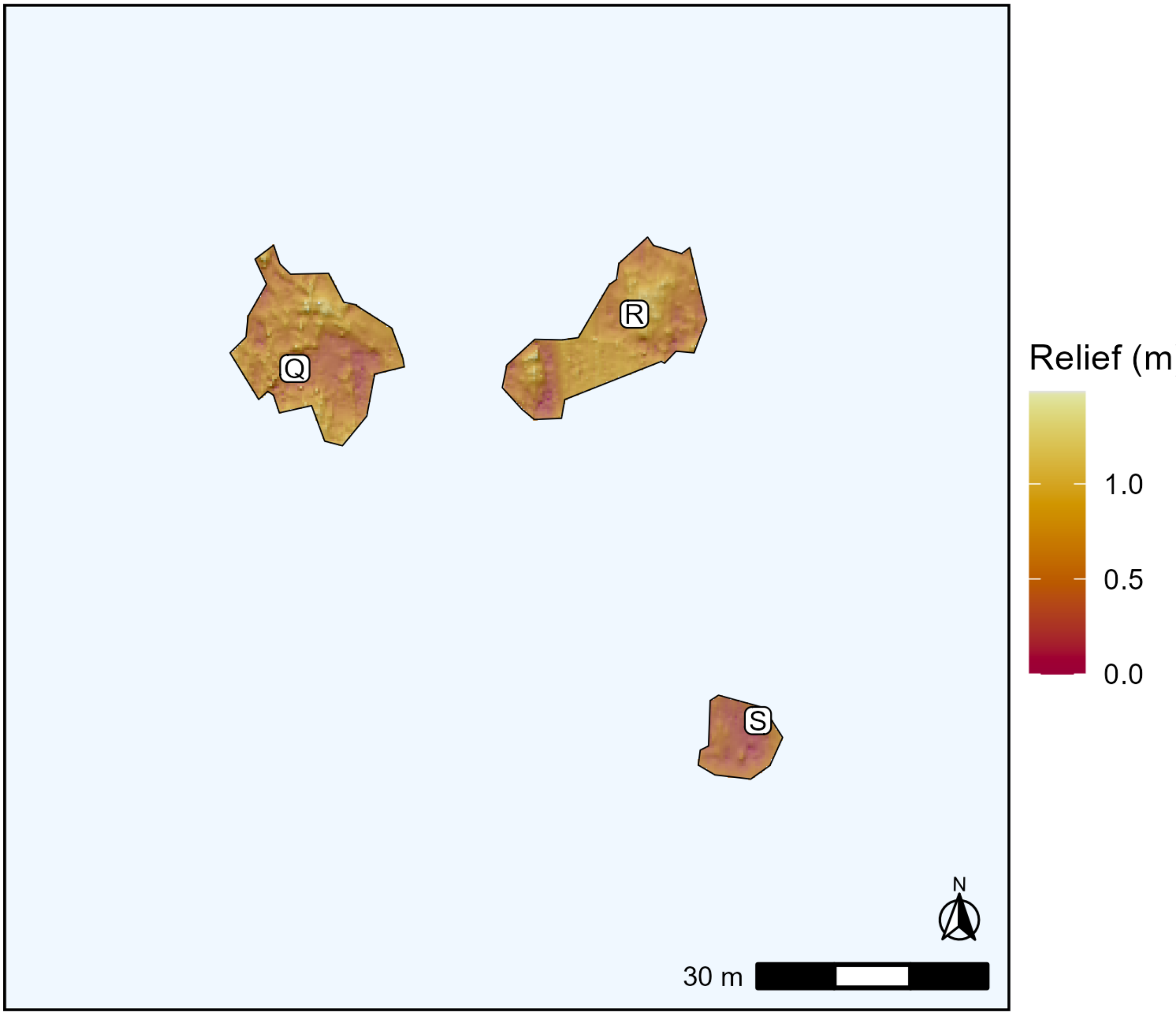

Figure A11: Map of the 3 module(s) in Marina Del Rey 1 Artificial Reef Complex. Sonar derived bathymetry was used to generate estimates of vertical relief in m (color).

Table A5: Characteristics of the 3 module(s) in Marina Del Rey 1 Artificial Reef Complex. Module naming attempted to preserve historical module spatial designations and names (Lewis and McKee 2001). The reported primary construction material was based on historical descriptions in the literature and that we confirmed from the sonar data or (where possible) visually during SCUBA or ROV surveys. Latitude and Longitude are center points of each module. Sonar derived bathymetry was used to generate estimates of average depth (m) and maximum vertical relief (m), while the 2d module footprint area was determined through a habitat classification process described in the methods. Note that for modules built from quarry rock, their areas are largely representative of the extent of contiguous material, while for most modules built from concrete material (e.g., pier pilings) these area estimates are more subjective, often inclusive of patches of soft sediment among scattered reef material. Frequently adjcent modules were originally classified as contigous reef habitat before being divided to maintain historical spatial designations and names (Lewis and McKee 2001).

Module	Material	Year Constructed (Augmented)	Latitude	Longitude	Depth (m)	Relief (m)	Footprint (m^2)	Volume (m^3)
Q	quarry rock	1965	33.96599	-118.48739	22.0	0.9	314	119
R	concrete dock floats	1965 (1976)	33.96601	-118.48696	21.4	0.9	295	126
S	concrete rubble	1965 (1978)	33.96554	-118.48678	21.5	0.5	85	19

7 Hermosa Beach Artificial Reef

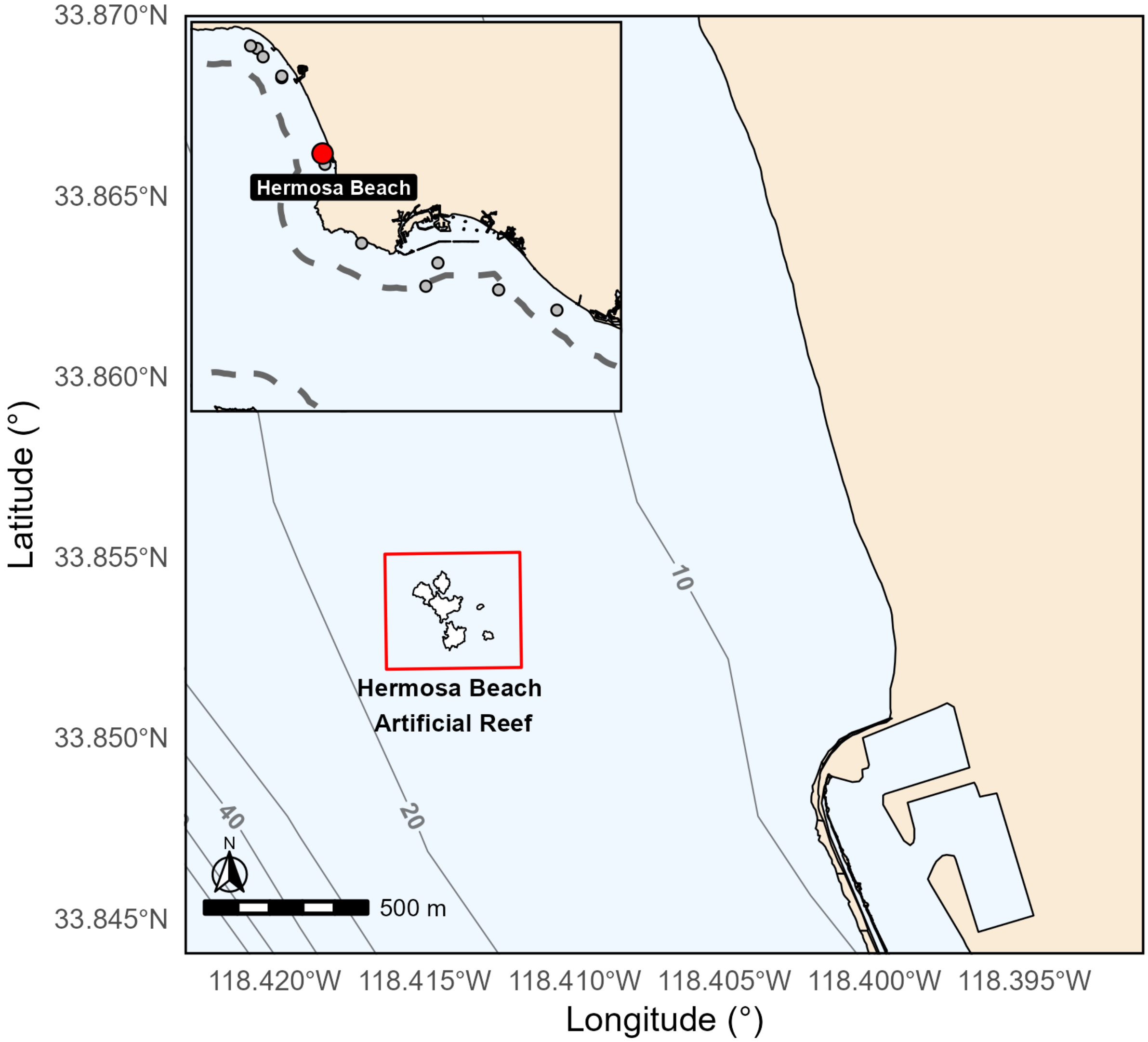

Figure A12: Location of Hermosa Beach Artificial Reef Complex. The red bounding box indicates the area included in the AR module detail map (Figure A13). The boundary between state and federal waters (dashed line, not visible in main plot for some AR complexes) is generally located 3 nautical miles offshore. Bathymetry contours are shown in meters and were derived from the NOAA ETOPO 2022 15 Arc-Second Global Relief Model using the marmap R package.

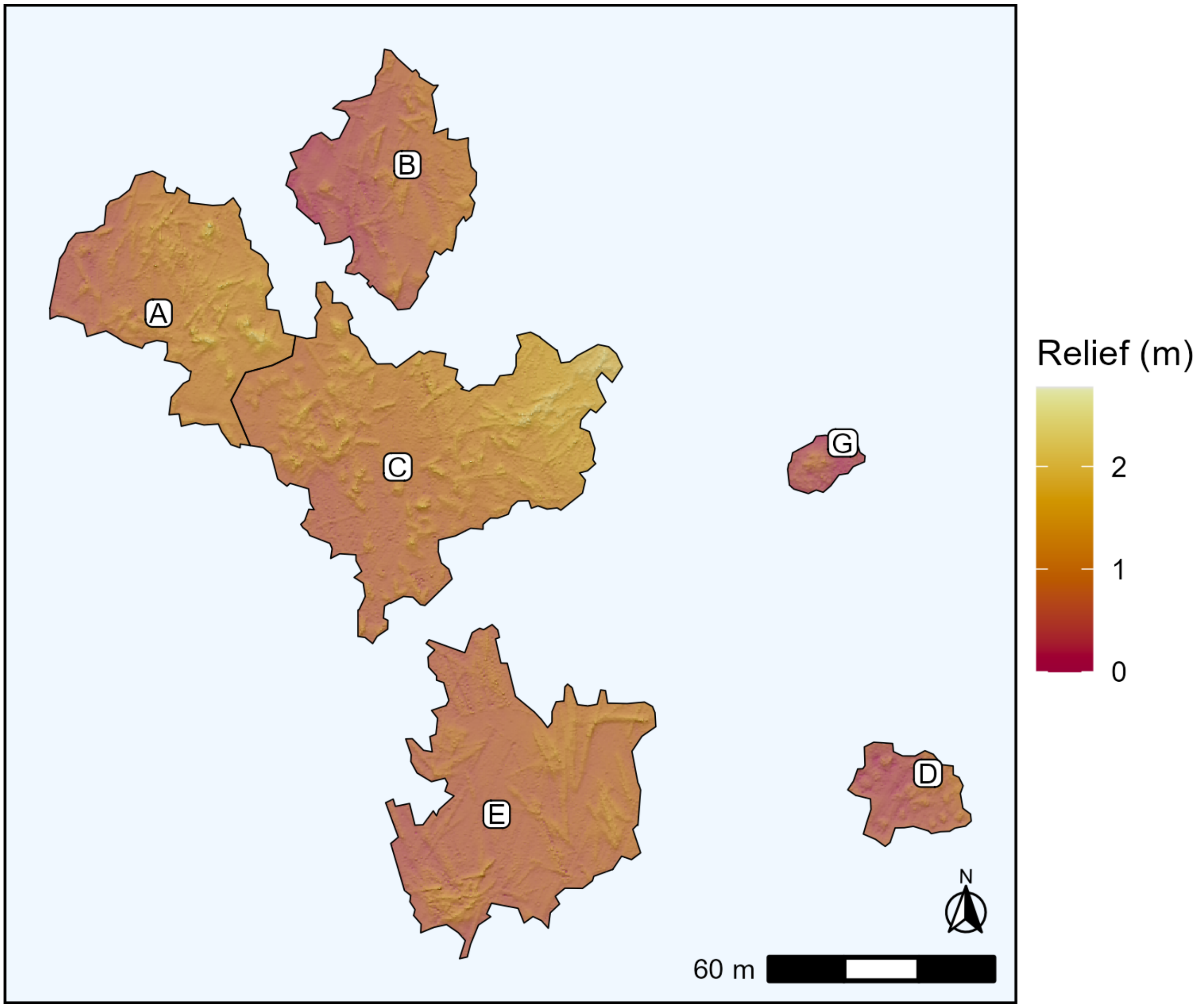

Figure A13: Map of the 6 module(s) in Hermosa Beach Artificial Reef Complex. Sonar derived bathymetry was used to generate estimates of vertical relief in m (color).

Table A6: Characteristics of the 6 module(s) in Hermosa Beach Artificial Reef Complex. Module naming attempted to preserve historical module spatial designations and names (Lewis and McKee 2001). The reported primary construction material was based on historical descriptions in the literature and that we confirmed from the sonar data or (where possible) visually during SCUBA or ROV surveys. Latitude and Longitude are center points of each module. Sonar derived bathymetry was used to generate estimates of average depth (m) and maximum vertical relief (m), while the 2d module footprint area was determined through a habitat classification process described in the methods. Note that for modules built from quarry rock, their areas are largely representative of the extent of contiguous material, while for most modules built from concrete material (e.g., pier pilings) these area estimates are more subjective, often inclusive of patches of soft sediment among scattered reef material. Frequently adjcent modules were originally classified as contigous reef habitat before being divided to maintain historical spatial designations and names (Lewis and McKee 2001).

Module	Material	Year Constructed (Augmented)	Latitude	Longitude	Depth (m)	Relief (m)	Footprint (m^2)	Volume (m^3)
A	concrete pilings; quarry rock; concrete shelters	1960	33.85401	-118.41501	19.2	1.2	2,474	1,284
B	concrete pilings	1960	33.85428	-118.41441	18.6	0.9	1,942	803
C	concrete pilings; quarry rock; concrete shelters	1960	33.85364	-118.41432	18.8	1.3	4,578	2,213
D	quarry rock; concrete shelters	1960	33.85283	-118.41288	18.2	0.7	564	187
E	concrete pilings	1960	33.85281	-118.41402	19.0	0.7	3,549	1,060
G	quarry rock	1960	33.85362	-118.41314	17.6	0.7	217	53

8 Redondo Beach Artificial Reef

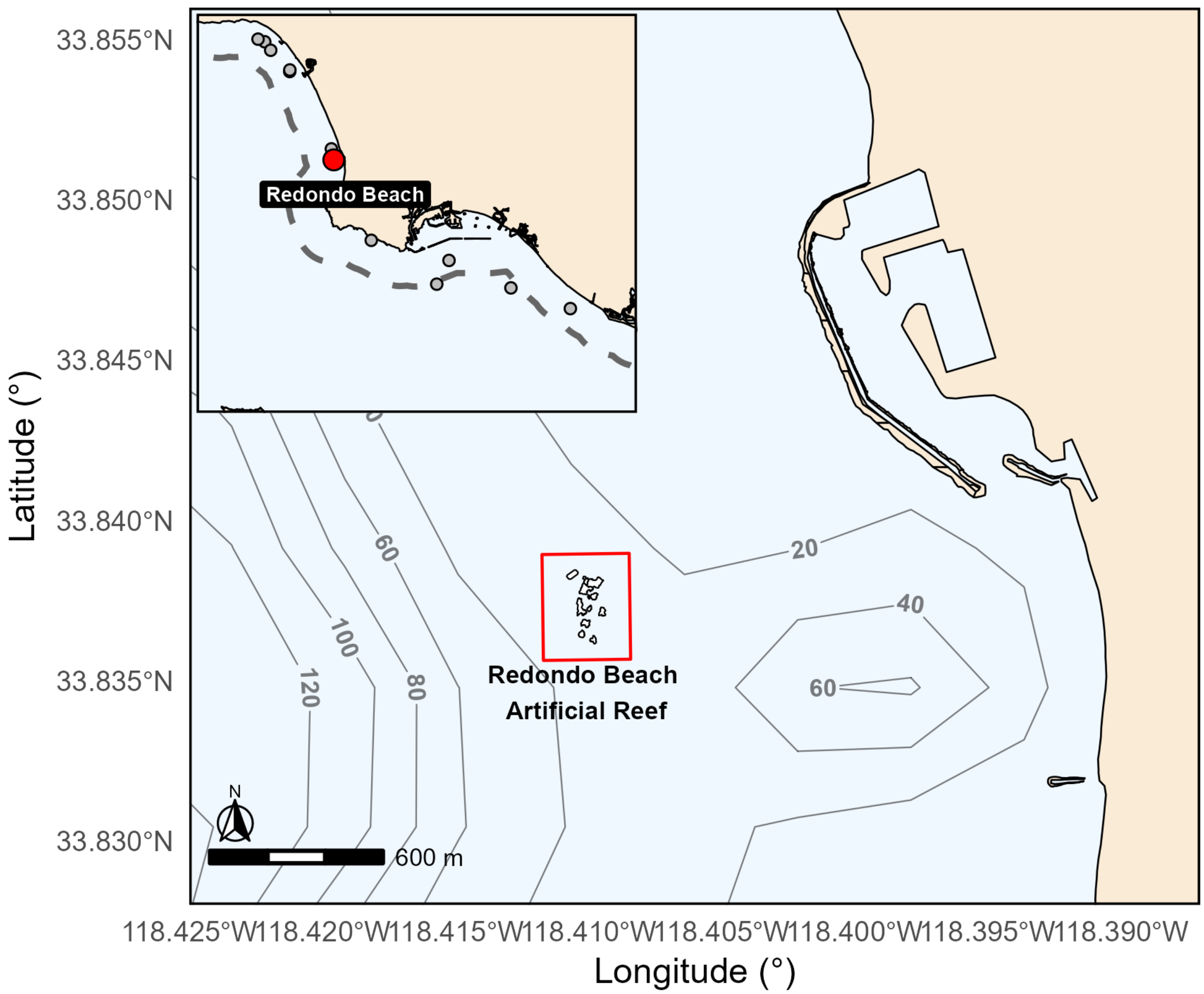

Figure A14: Location of Redondo Beach Artificial Reef Complex. The red bounding box indicates the area included in the AR module detail map (Figure A15). The boundary between state and federal waters (dashed line, not visible in main plot for some AR complexes) is generally located 3 nautical miles offshore. Bathymetry contours are shown in meters and were derived from the NOAA ETOPO 2022 15 Arc-Second Global Relief Model using the marmap R package.

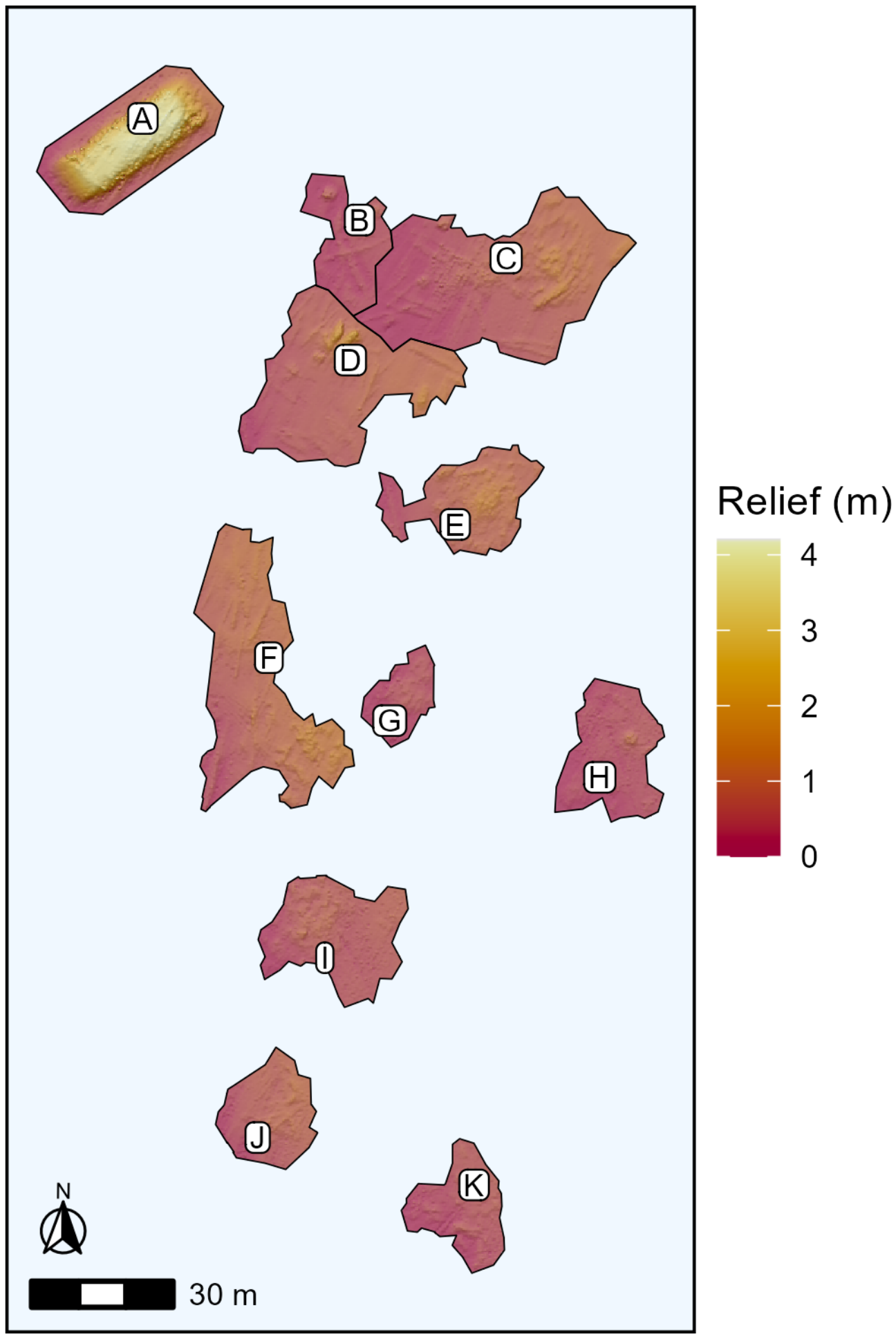

Figure A15: Map of the 11 module(s) in Redondo Beach Artificial Reef Complex. Sonar derived bathymetry was used to generate estimates of vertical relief in m (color).

Table A7: Characteristics of the 11 module(s) in Redondo Beach Artificial Reef Complex. Module naming attempted to preserve historical module spatial designations and names (Lewis and McKee 2001). The reported primary construction material was based on historical descriptions in the literature and that we confirmed from the sonar data or (where possible) visually during SCUBA or ROV surveys. Latitude and Longitude are center points of each module. Sonar derived bathymetry was used to generate estimates of average depth (m) and maximum vertical relief (m), while the 2d module footprint area was determined through a habitat classification process described in the methods. Note that for modules built from quarry rock, their areas are largely representative of the extent of contiguous material, while for most modules built from concrete material (e.g., pier pilings) these area estimates are more subjective, often inclusive of patches of soft sediment among scattered reef material. Frequently adjcent modules were originally classified as contigous reef habitat before being divided to maintain historical spatial designations and names (Lewis and McKee 2001).

Module	Material	Year Constructed (Augmented)	Latitude	Longitude	Depth (m)	Relief (m)	Footprint (m^2)	Volume (m^3)
A	barge	1974	33.83831	-118.41059	23.1	3.8	685	1,188
B	quarry rock; concrete pilings/pipe	1962 (1976)	33.83812	-118.41009	23.7	0.5	333	67
C	quarry rock; concrete pilings/pipe	1962 (1976)	33.83805	-118.40976	23.1	1.1	1,306	641
D	quarry rock; concrete pilings/pipe	1962 (1976)	33.83785	-118.41011	23.7	1.3	967	509
E	quarry rock; concrete dock floats	1962 (1975)	33.83763	-118.40981	23.1	1.1	465	277
F	quarry rock; concrete pilings/pipe	1962 (1976)	33.83728	-118.41029	24.5	1.1	1,024	629
G	quarry rock	1962	33.83725	-118.40996	23.6	0.6	198	54
H	concrete dock floats	1962 (1978)	33.83714	-118.40947	22.9	0.7	472	126
I	quarry rock; concrete dock floats	1962 (1978)	33.83679	-118.41010	24.5	0.8	554	236
J	quarry rock	1962	33.83645	-118.41025	25.3	0.9	362	176
K	quarry rock	1962	33.83627	-118.40980	24.5	0.6	342	115

9 Palos Verdes Reef

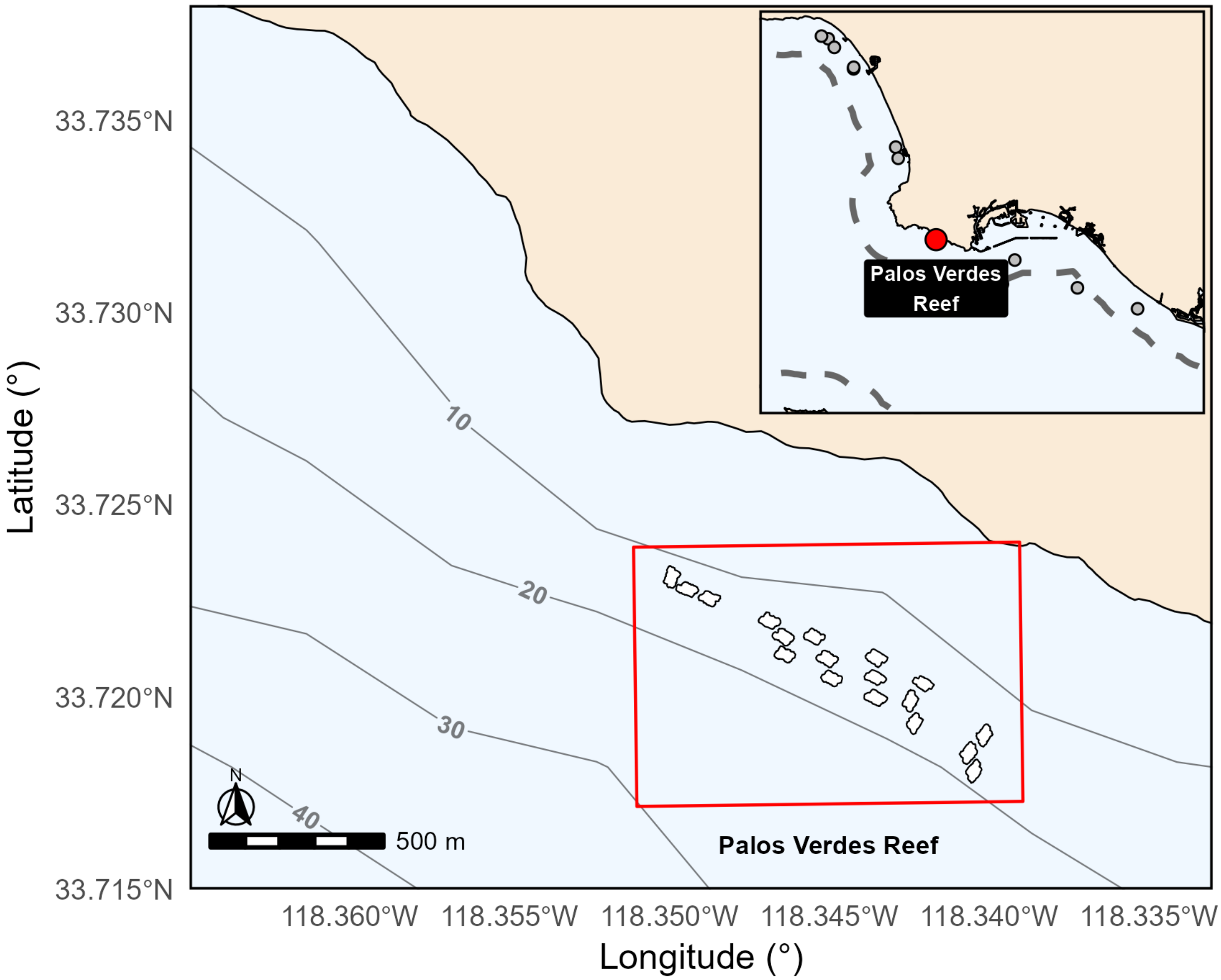

Figure A16: Location of Palos Verdes Reef Complex. The red bounding box indicates the area included in the AR module detail map (Figure A17). The boundary between state and federal waters (dashed line, not visible in main plot for some AR complexes) is generally located 3 nautical miles offshore. Bathymetry contours are shown in meters and were derived from the NOAA ETOPO 2022 15 Arc-Second Global Relief Model using the marmap R package.

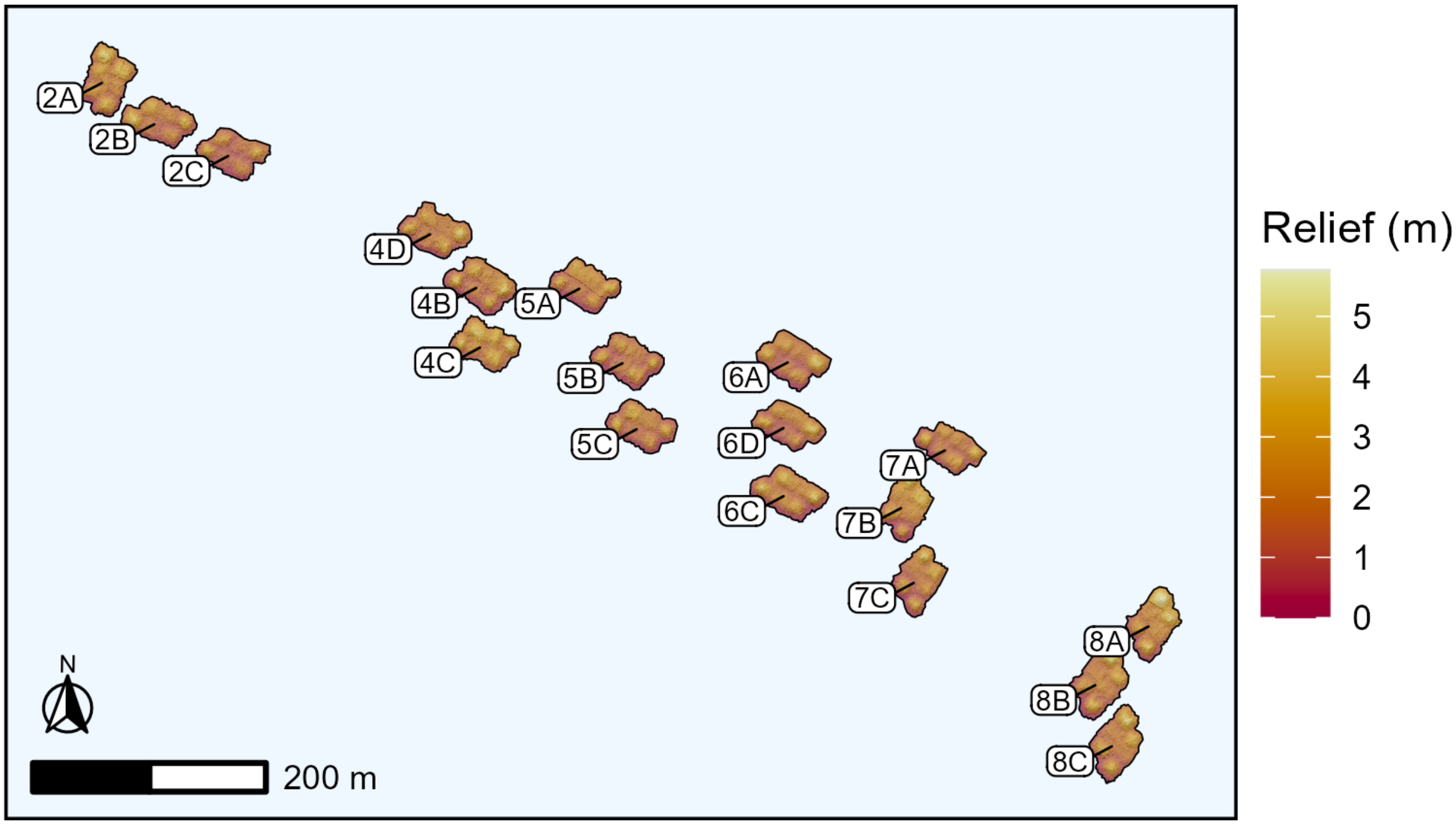

Figure A17: Map of the 18 module(s) in Palos Verdes Reef Complex. Sonar derived bathymetry was used to generate estimates of vertical relief in m (color).

Table A8: Characteristics of the 18 module(s) in Palos Verdes Reef Complex. Module naming attempted to preserve historical module spatial designations and names (Lewis and McKee 2001). The reported primary construction material was based on historical descriptions in the literature and that we confirmed from the sonar data or (where possible) visually during SCUBA or ROV surveys. Latitude and Longitude are center points of each module. Sonar derived bathymetry was used to generate estimates of average depth (m) and maximum vertical relief (m), while the 2d module footprint area was determined through a habitat classification process described in the methods. Note that for modules built from quarry rock, their areas are largely representative of the extent of contiguous material, while for most modules built from concrete material (e.g., pier pilings) these area estimates are more subjective, often inclusive of patches of soft sediment among scattered reef material. Frequently adjcent modules were originally classified as contigous reef habitat before being divided to maintain historical spatial designations and names (Lewis and McKee 2001).

Module	Material	Year Constructed (Augmented)	Latitude	Longitude	Depth (m)	Relief (m)	Footprint (m^2)	Volume (m^3)
2A	quarry rock	2020	33.72312	-118.34992	16.5	3.9	1,881	3,305
2B	quarry rock	2020	33.72281	-118.34943	17.3	3.5	1,773	2,473
2C	quarry rock	2020	33.72257	-118.34874	17.6	3.0	1,701	1,946
4B	quarry rock	2020	33.72157	-118.34642	17.8	3.7	1,884	2,877
4C	quarry rock	2020	33.72111	-118.34638	19.2	3.5	1,742	2,795
4D	quarry rock	2020	33.72198	-118.34686	17.1	3.7	1,824	2,831
5A	quarry rock	2020	33.72157	-118.34546	16.4	3.0	1,726	2,664
5B	quarry rock	2020	33.72100	-118.34505	17.9	2.8	1,824	2,310
5C	quarry rock	2020	33.72049	-118.34492	19.0	3.1	1,749	2,485
6A	quarry rock	2020	33.72102	-118.34352	15.8	3.6	1,902	2,789
6C	quarry rock	2020	33.71999	-118.34354	18.9	3.8	1,922	3,127
6D	quarry rock	2020	33.72051	-118.34355	17.7	3.6	1,677	2,529
7A	quarry rock	2020	33.72036	-118.34205	15.5	3.3	1,596	2,327
7B	quarry rock	2020	33.71990	-118.34245	17.8	4.1	1,743	3,382
7C	quarry rock	2020	33.71933	-118.34232	19.6	3.5	1,677	2,515
8A	quarry rock	2020	33.71901	-118.34014	16.5	4.7	1,772	3,520
8B	quarry rock	2020	33.71855	-118.34063	19.0	4.0	1,929	3,315
8C	quarry rock	2020	33.71808	-118.34047	19.7	3.9	1,838	3,052

10 Georgia Straits Shipwreck

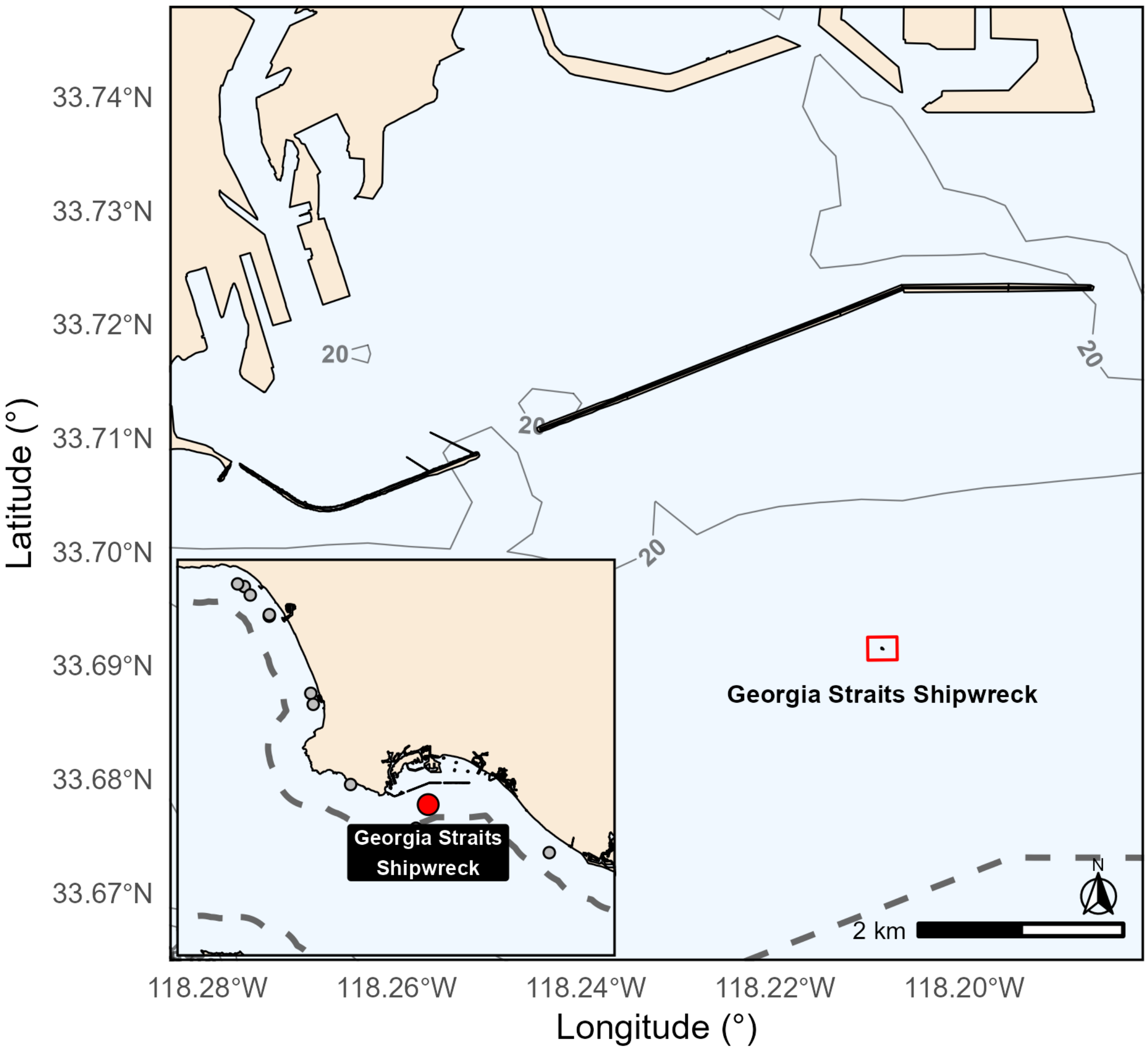

Figure A18: Location of Georgia Straits Shipwreck. The red bounding box indicates the area included in the AR module detail map (Figure A19). The boundary between state and federal waters (dashed line, not visible in main plot for some AR complexes) is generally located 3 nautical miles offshore. Bathymetry contours are shown in meters and were derived from the NOAA ETOPO 2022 15 Arc-Second Global Relief Model using the marmap R package.

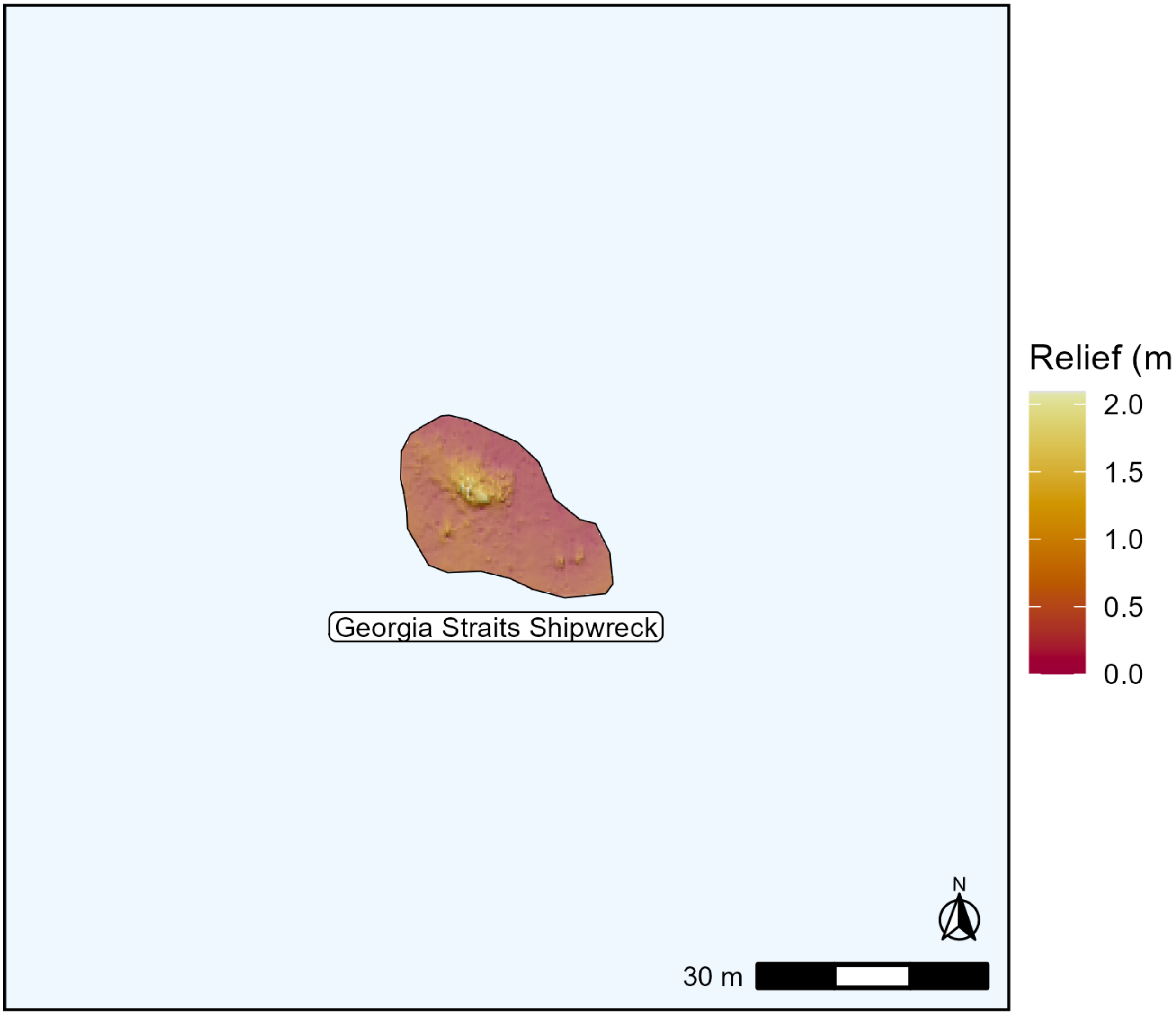

Figure A19: Map of the 1 module(s) in Georgia Straits Shipwreck. Sonar derived bathymetry was used to generate estimates of vertical relief in m (color).

Table A9: Characteristics of the 1 module(s) in Georgia Straits Shipwreck. Module naming attempted to preserve historical module spatial designations and names (Lewis and McKee 2001). The reported primary construction material was based on historical descriptions in the literature and that we confirmed from the sonar data or (where possible) visually during SCUBA or ROV surveys. Latitude and Longitude are center points of each module. Sonar derived bathymetry was used to generate estimates of average depth (m) and maximum vertical relief (m), while the 2d module footprint area was determined through a habitat classification process described in the methods. Note that for modules built from quarry rock, their areas are largely representative of the extent of contiguous material, while for most modules built from concrete material (e.g., pier pilings) these area estimates are more subjective, often inclusive of patches of soft sediment among scattered reef material. Frequently adjcent modules were originally classified as contigous reef habitat before being divided to maintain historical spatial designations and names (Lewis and McKee 2001).

Module	Material	Year Constructed (Augmented)	Latitude	Longitude	Depth (m)	Relief (m)	Footprint (m^2)	Volume (m^3)
Georgia Straits	shipwreck	1965	33.69148	-118.20866	21.7	1.2	426	116

11 Olympic Shipwreck

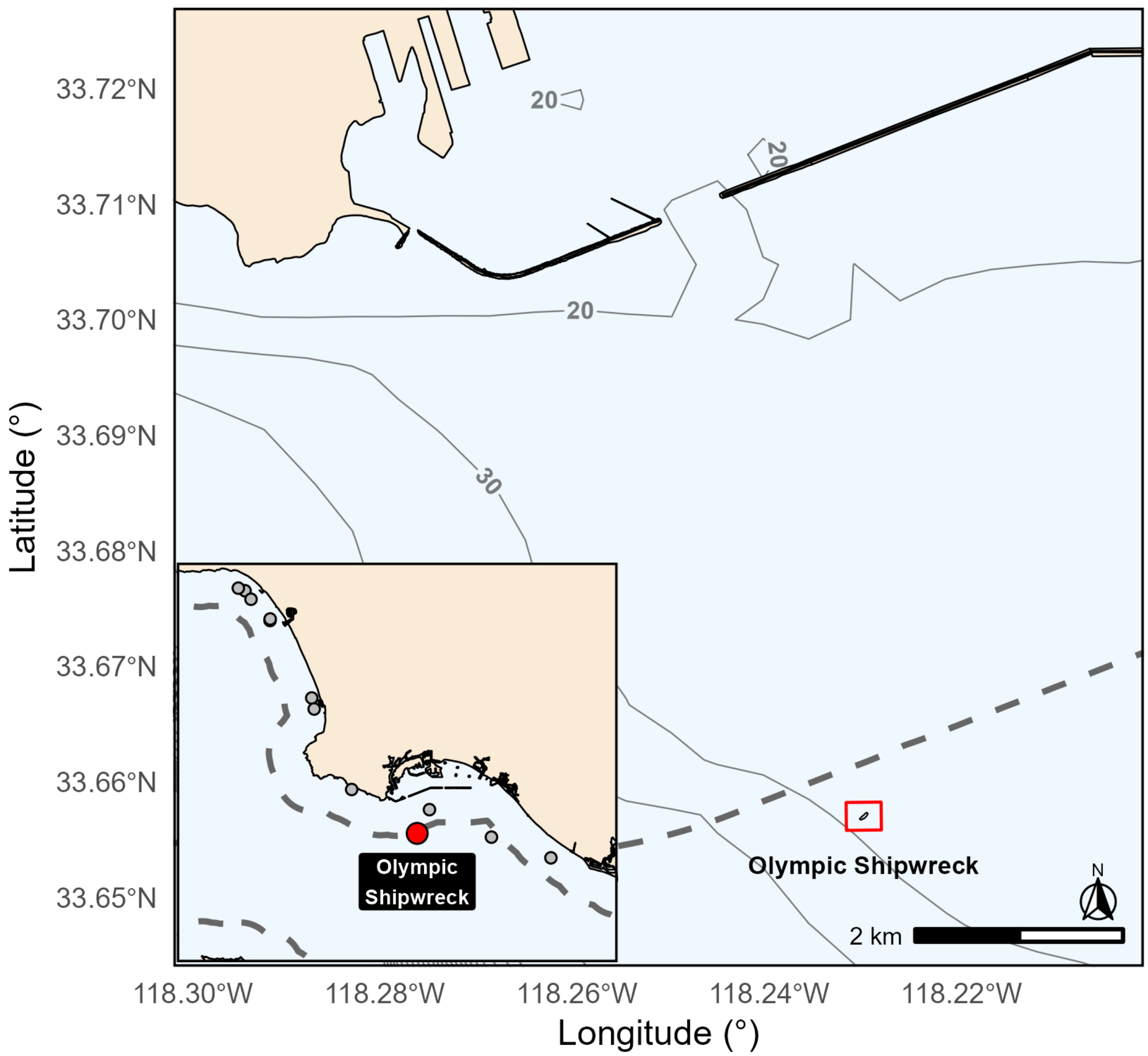

Figure A20: Location of Olympic Shipwreck. The red bounding box indicates the area included in the AR module detail map (Figure A21). The boundary between state and federal waters (dashed line, not visible in main plot for some AR complexes) is generally located 3 nautical miles offshore. Bathymetry contours are shown in meters and were derived from the NOAA ETOPO 2022 15 Arc-Second Global Relief Model using the marmap R package.

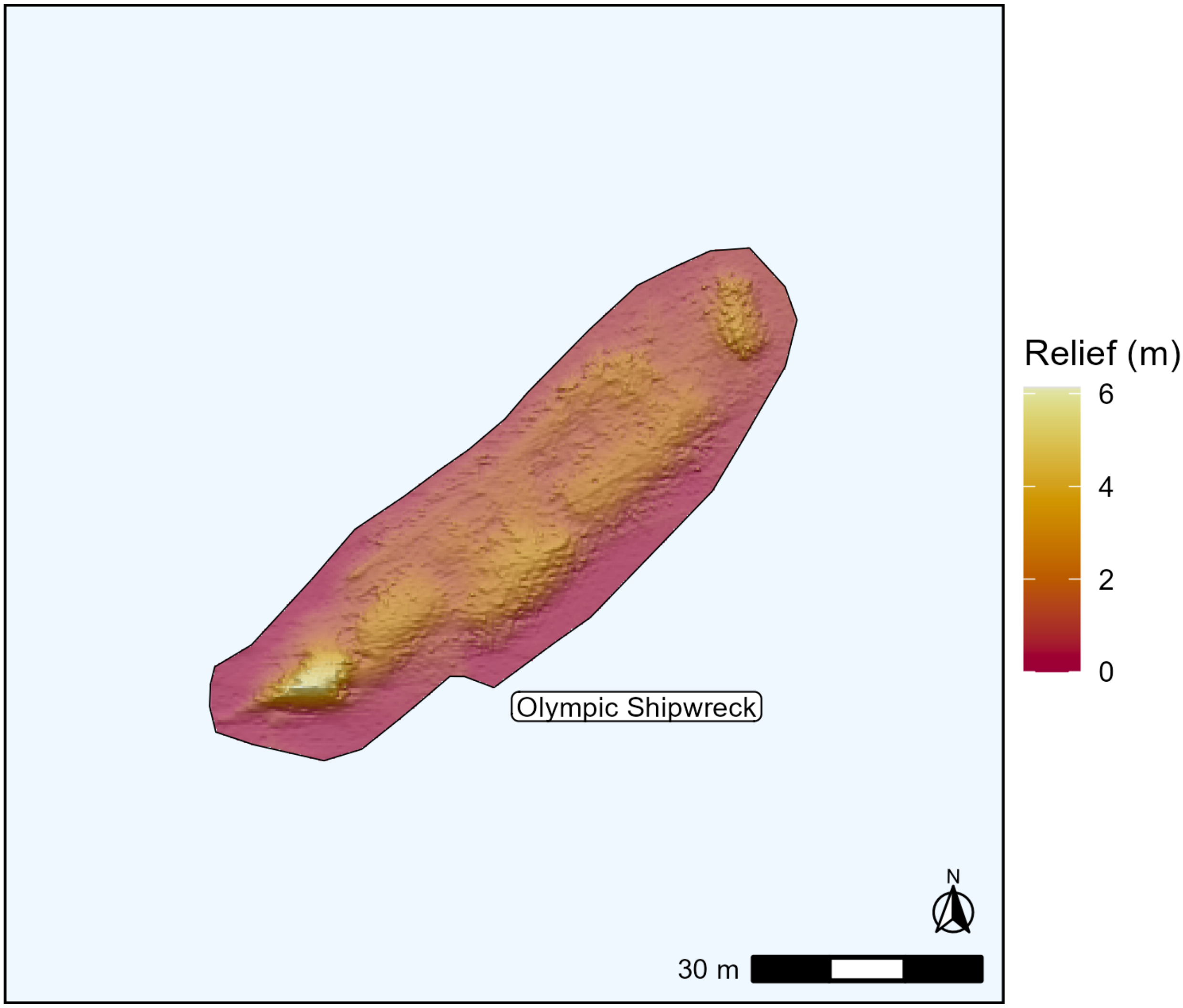

Figure A21: Map of the Olympic Shipwreck. Sonar derived bathymetry was used to generate estimates of vertical relief in m (color).

Table A10: Characteristics of the 1 module(s) in Olympic Shipwreck. Module naming attempted to preserve historical module spatial designations and names (Lewis and McKee 2001). The reported primary construction material was based on historical descriptions in the literature and that we confirmed from the sonar data or (where possible) visually during SCUBA or ROV surveys. Latitude and Longitude are center points of each module. Sonar derived bathymetry was used to generate estimates of average depth (m) and maximum vertical relief (m), while the 2d module footprint area was determined through a habitat classification process described in the methods. Note that for modules built from quarry rock, their areas are largely representative of the extent of contiguous material, while for most modules built from concrete material (e.g., pier pilings) these area estimates are more subjective, often inclusive of patches of soft sediment among scattered reef material. Frequently adjcent modules were originally classified as contigous reef habitat before being divided to maintain historical spatial designations and names (Lewis and McKee 2001).

Module	Material	Year Constructed (Augmented)	Latitude	Longitude	Depth (m)	Relief (m)	Foot-print (m^2)	Vol-ume (m^3)
Olympic	shipwreck	1940	33.65701	-118.23012	28.1	3.7	2,158	2,382

12 Bolsa Chica Artificial Reef

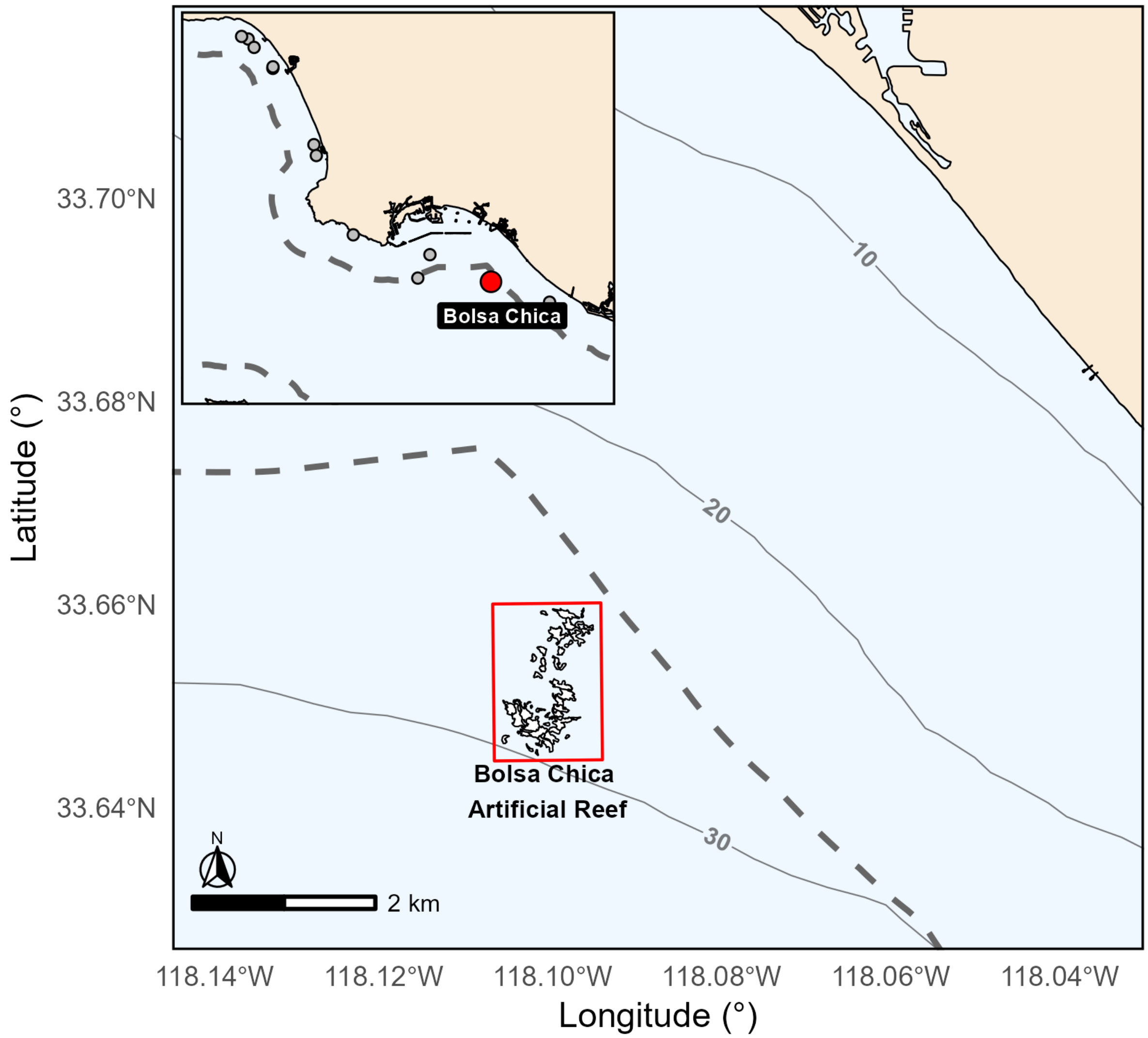

Figure A22: Location of Bolsa Chica Artificial Reef Complex. The red bounding box indicates the area included in the AR module detail map (Figure A23). The boundary between state and federal waters (dashed line, not visible in main plot for some AR complexes) is generally located 3 nautical miles offshore. Bathymetry contours are shown in meters and were derived from the NOAA ETOPO 2022 15 Arc-Second Global Relief Model using the marmap R package.

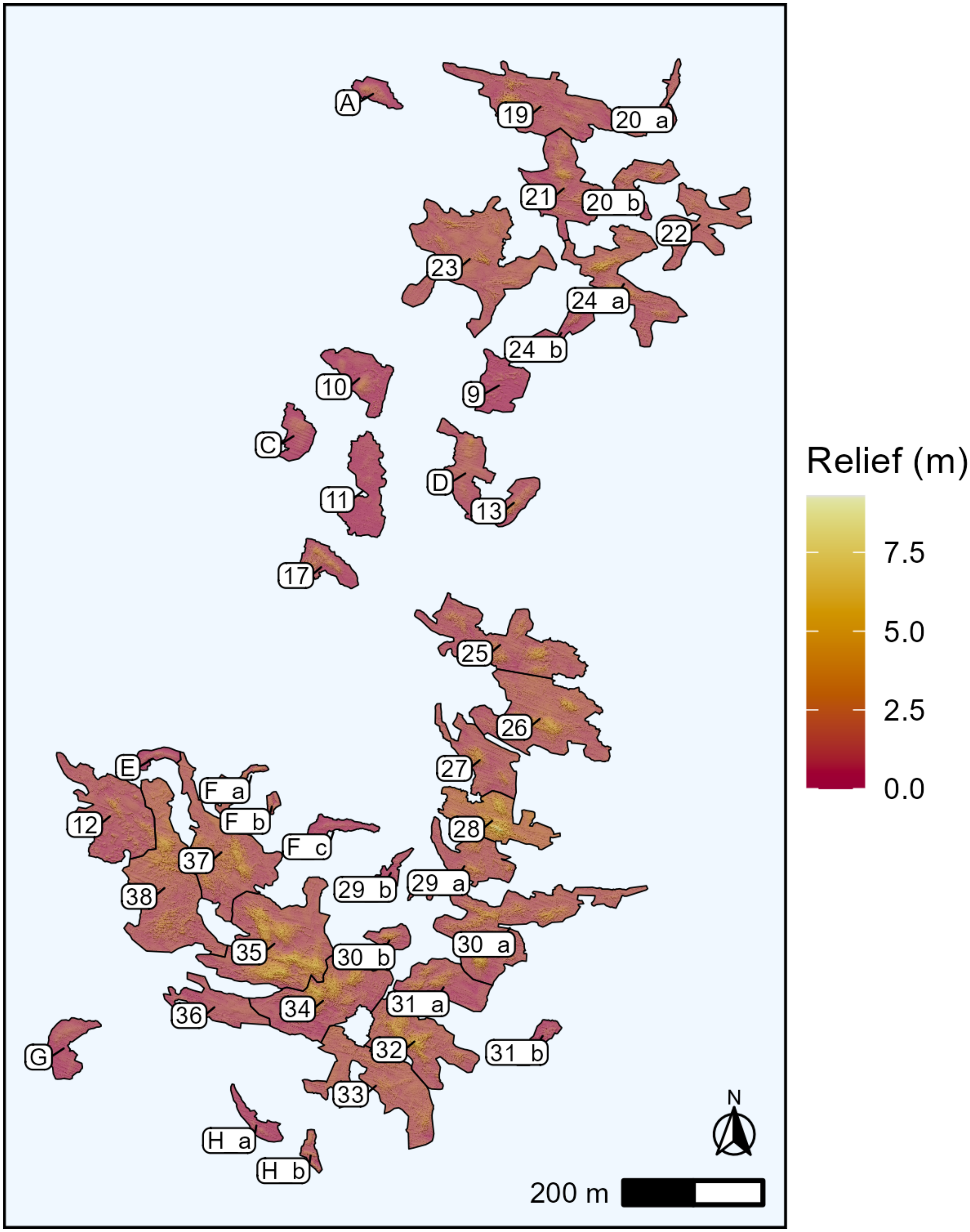

Figure A23: Map of the 41 module(s) in Bolsa Chica Artificial Reef Complex. Sonar derived bathymetry was used to generate estimates of vertical relief in m (color).

Table A11: Characteristics of the 41 module(s) in Bolsa Chica Artificial Reef Complex. Module naming attempted to preserve historical module spatial designations and names (Lewis and McKee 2001). The reported primary construction material was based on historical descriptions in the literature and that we confirmed from the sonar data or (where possible) visually during SCUBA or ROV surveys. Latitude and Longitude are center points of each module. Sonar derived bathymetry was used to generate estimates of average depth (m) and maximum vertical relief (m), while the 2d module footprint area was determined through a habitat classification process described in the methods. Note that for modules built from quarry rock, their areas are largely representative of the extent of contiguous material, while for most modules built from concrete material (e.g., pier pilings) these area estimates are more subjective, often inclusive of patches of soft sediment among scattered reef material. Frequently adjcent modules were originally classified as contigous reef habitat before being divided to maintain historical spatial designations and names (Lewis and McKee 2001).

Module	Material	Year Constructed (Augmented)	Latitude	Longitude	Depth (m)	Relief (m)	Foot-print (m^2)	Vol-ume (m^3)
10	concrete chimneys	1986 (1993)	33.65550	-118.10118	27.4	1.9	5,095	2,360
11	concrete light poles	1986 (1996)	33.65403	-118.10111	28.1	0.9	6,489	2,612
12	shipyard concrete ; concrete pilings	1986 (1995)	33.64978	-118.10498	29.4	2.2	11,644	7,551
13	concrete light poles	1986 (1997)	33.65391	-118.09876	27.4	2.3	2,317	1,602
17	concrete light poles	1986 (1997)	33.65304	-118.10174	27.9	2.5	3,389	2,782
19	concrete pilings; steel girder; concrete rubble	1986 (1999)	33.65905	-118.09841	25.9	3.1	13,784	9,448
20_a	concrete rubble	1986 (1999)	33.65912	-118.09645	25.6	1.1	1,373	695
20_b	concrete pilings	1986 (1999)	33.65805	-118.09688	25.8	2.4	3,725	3,355
21	concrete pilings	1986 (1999)	33.65799	-118.09804	26.1	2.6	9,617	7,745
22	concrete pilings	1986 (1999)	33.65754	-118.09593	26.2	1.6	7,426	4,326
23	concrete pilings	1986 (1999)	33.65706	-118.09949	26.7	2.3	18,049	12,428
24_a	concrete pilings	1986 (1999)	33.65677	-118.09710	26.4	3.1	10,194	8,472
24_b	concrete pilings	1986 (1999)	33.65613	-118.09806	26.5	1.7	2,637	1,330
25	concrete pilings	1986 (1999)	33.65206	-118.09895	28.0	2.7	12,866	9,911
26	concrete pilings	1986 (1999)	33.65111	-118.09833	28.2	3.3	14,881	12,995
27	concrete pilings	1986 (1999)	33.65056	-118.09925	28.6	2.7	6,710	5,317
28	concrete pilings	1986 (1999)	33.64978	-118.09905	28.6	4.1	7,674	7,509

29_a	concrete pilings	1986 (1999)	33.64920	-118.09948	29.0	2.2	6,697	4,655
29_b	concrete pilings	1986 (1999)	33.64910	-118.10062	29.1	1.0	1,047	462
30_a	concrete pilings	1986 (1999)	33.64840	-118.09877	29.1	3.1	15,641	14,587
30_b	concrete pilings	1986 (1999)	33.64823	-118.10063	29.2	3.0	1,533	1,243
31_a	concrete pilings	1986 (1999)	33.64763	-118.09978	29.4	2.6	5,865	4,702
31_b	concrete pilings	1986 (1999)	33.64701	-118.09824	29.7	1.0	1,045	367
32	concrete pilings	1986 (1999)	33.64690	-118.10022	29.5	3.8	10,001	10,606
33	concrete pilings	1986 (1999)	33.64632	-118.10081	30.2	1.7	11,857	9,314
34	concrete pilings; steel girder	1986 (1999)	33.64742	-118.10164	29.2	4.3	12,948	14,731
35	concrete pilings	1986 (1999)	33.64815	-118.10241	28.9	4.5	18,130	24,146
36	concrete pilings	1986 (1999)	33.64732	-118.10333	30.2	1.2	5,005	3,177
37	concrete pilings	1986 (1999)	33.64933	-118.10324	29.2	3.1	14,589	13,475
38	concrete pilings	1986 (1999)	33.64886	-118.10412	29.7	2.9	17,062	15,803
9	concrete chimneys	1986 (1992)	33.65542	-118.09901	27.3	1.0	4,403	1,574
A	concrete rubble	1986	33.65918	-118.10101	25.9	2.2	1,795	1,217
C	concrete rubble	1986	33.65473	-118.10219	27.6	1.5	2,725	1,360
D	concrete rubble	1986	33.65427	-118.09952	27.4	1.9	5,587	3,406
E	concrete rubble	1986	33.65053	-118.10432	29.0	1.5	1,187	563
F_a	barge	1986	33.65035	-118.10282	28.8	1.7	406	350
F_b	barge	1986	33.64996	-118.10249	28.7	1.8	438	449
F_c	concrete rubble	1986	33.64963	-118.10153	29.3	1.0	1,852	791
G	concrete rubble	1986	33.64676	-118.10566	30.5	1.3	3,606	1,865
H_a	concrete rubble	1986	33.64582	-118.10269	30.7	1.0	1,791	608
H_b	barge	1986	33.64544	-118.10184	30.8	1.9	955	865

13 Huntington Beach Artificial Reef

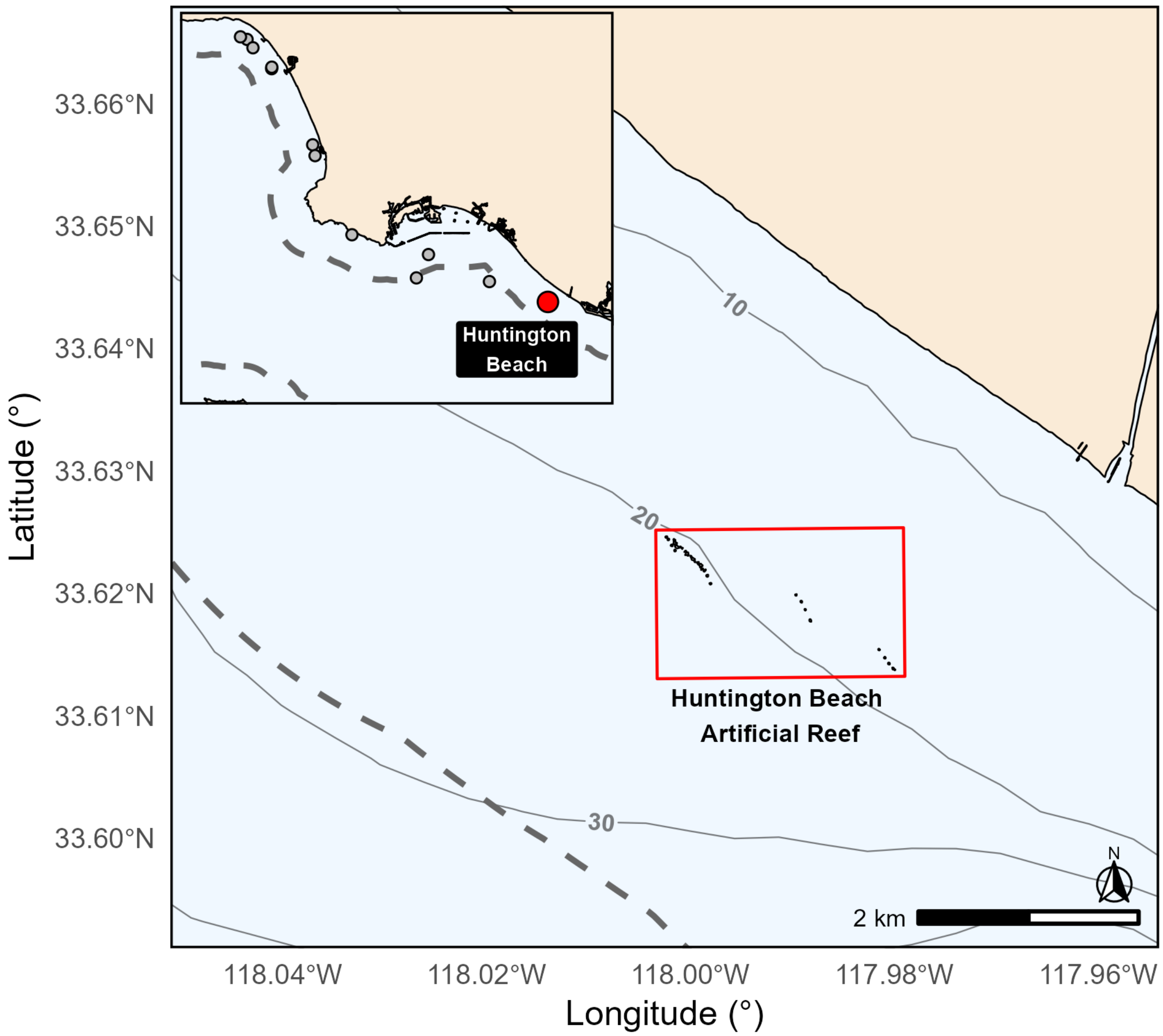

Figure A24: Location of Huntington Beach Artificial Reef Complex. The red bounding box indicates the area included in the AR module detail map (Figure A25). The boundary between state and federal waters (dashed line, not visible in main plot for some AR complexes) is generally located 3 nautical miles offshore. Bathymetry contours are shown in meters and were derived from the NOAA ETOPO 2022 15 Arc-Second Global Relief Model using the marmap R package.

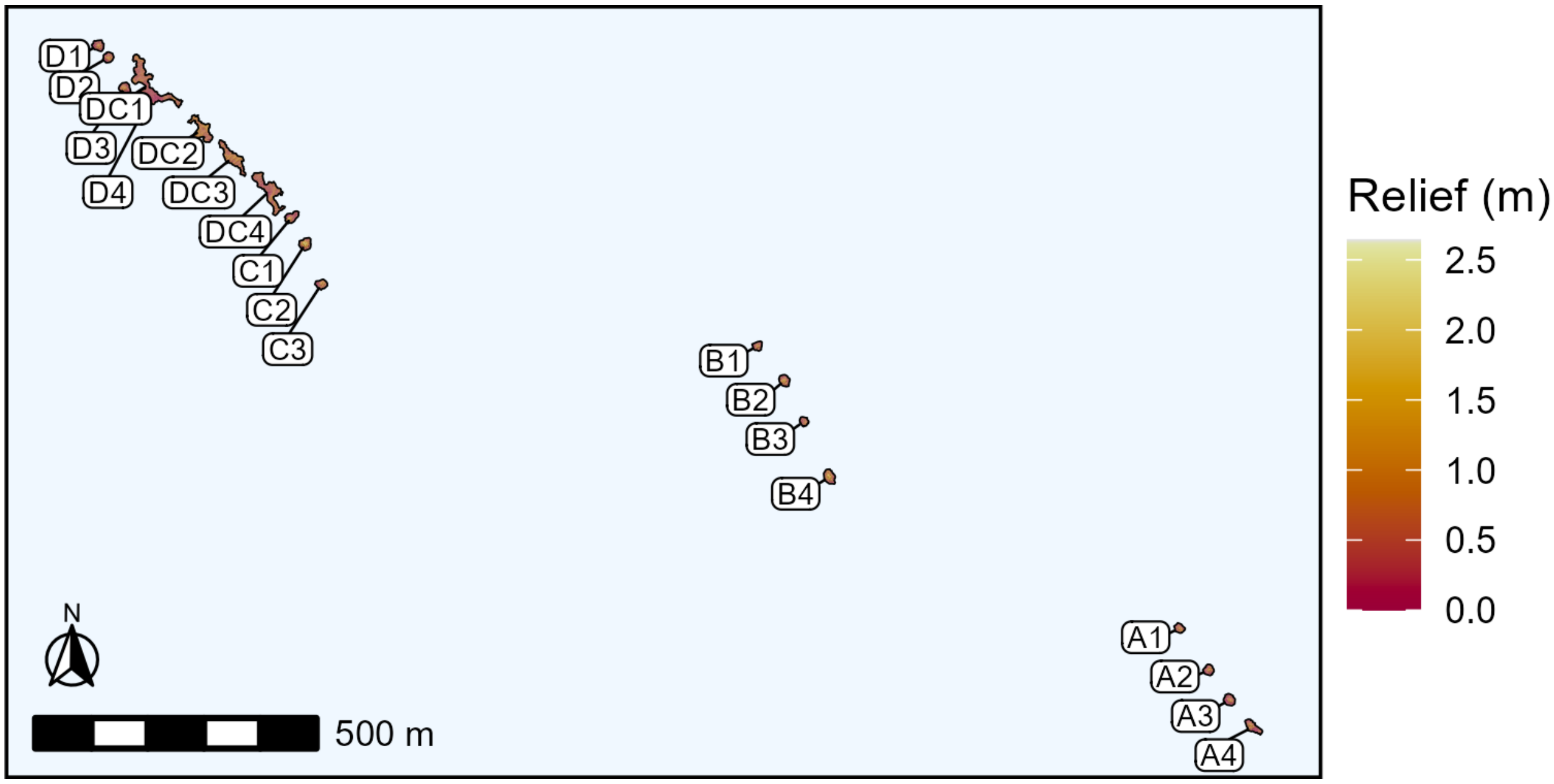

Figure A25: Map of the 19 module(s) in Huntington Beach Artificial Reef Complex. Sonar derived bathymetry was used to generate estimates of vertical relief in m (color).

Table A12: Characteristics of the 19 module(s) in Huntington Beach Artificial Reef Complex. Module naming attempted to preserve historical module spatial designations and names (Lewis and McKee 2001). The reported primary construction material was based on historical descriptions in the literature and that we confirmed from the sonar data or (where possible) visually during SCUBA or ROV surveys. Latitude and Longitude are center points of each module. Sonar derived bathymetry was used to generate estimates of average depth (m) and maximum vertical relief (m), while the 2d module footprint area was determined through a habitat classification process described in the methods. Note that for modules built from quarry rock, their areas are largely representative of the extent of contiguous material, while for most modules built from concrete material (e.g., pier pilings) these area estimates are more subjective, often inclusive of patches of soft sediment among scattered reef material. Frequently adjcent modules were originally classified as contigous reef habitat before being divided to maintain historical spatial designations and names (Lewis and McKee 2001).

Module	Material	Year Constructed (Augmented)	Latitude	Longitude	Depth (m)	Relief (m)	Footprint (m^2)	Volume (m^3)
A1	quarry rock	1963	33.61540	-117.98148	18.9	1.0	231	91
A2	quarry rock	1963	33.61473	-117.98091	19.2	1.0	270	97
A3	quarry rock	1963	33.61426	-117.98050	19.4	0.9	328	96
A4	quarry rock	1963	33.61382	-117.98005	19.5	1.0	420	161
B1	quarry rock	1963	33.61986	-117.98962	18.8	1.0	220	83
B2	quarry rock	1963	33.61931	-117.98910	18.9	1.1	306	116
B3	quarry rock	1963	33.61866	-117.98872	19.4	0.7	197	61
B4	quarry rock	1963	33.61777	-117.98822	19.4	1.5	403	219
C1	quarry rock	1963	33.62186	-117.99859	20.3	1.3	344	148
C2	quarry rock	1963	33.62143	-117.99832	19.9	1.7	362	205
C3	quarry rock	1963	33.62079	-117.99801	20.6	0.9	278	118
D1	quarry rock	1963	33.62458	-118.00233	19.7	1.0	312	94
D2	quarry rock	1963	33.62440	-118.00214	19.7	1.1	271	108
D3	quarry rock	1963	33.62391	-118.00181	19.9	1.1	293	122
D4	quarry rock	1963	33.62349	-118.00149	20.2	0.8	286	81
DC1	concrete pilings and cubes	1963	33.62397	-118.00135	20.3	0.9	2,132	854
DC2	concrete pilings and cubes	1963	33.62325	-118.00033	20.2	0.9	917	375
DC3	concrete pilings and cubes	1963	33.62281	-117.99974	20.3	0.9	960	370
DC4	concrete pilings and cubes	1963	33.62227	-117.99903	20.6	0.8	1,382	507

14 International Reef

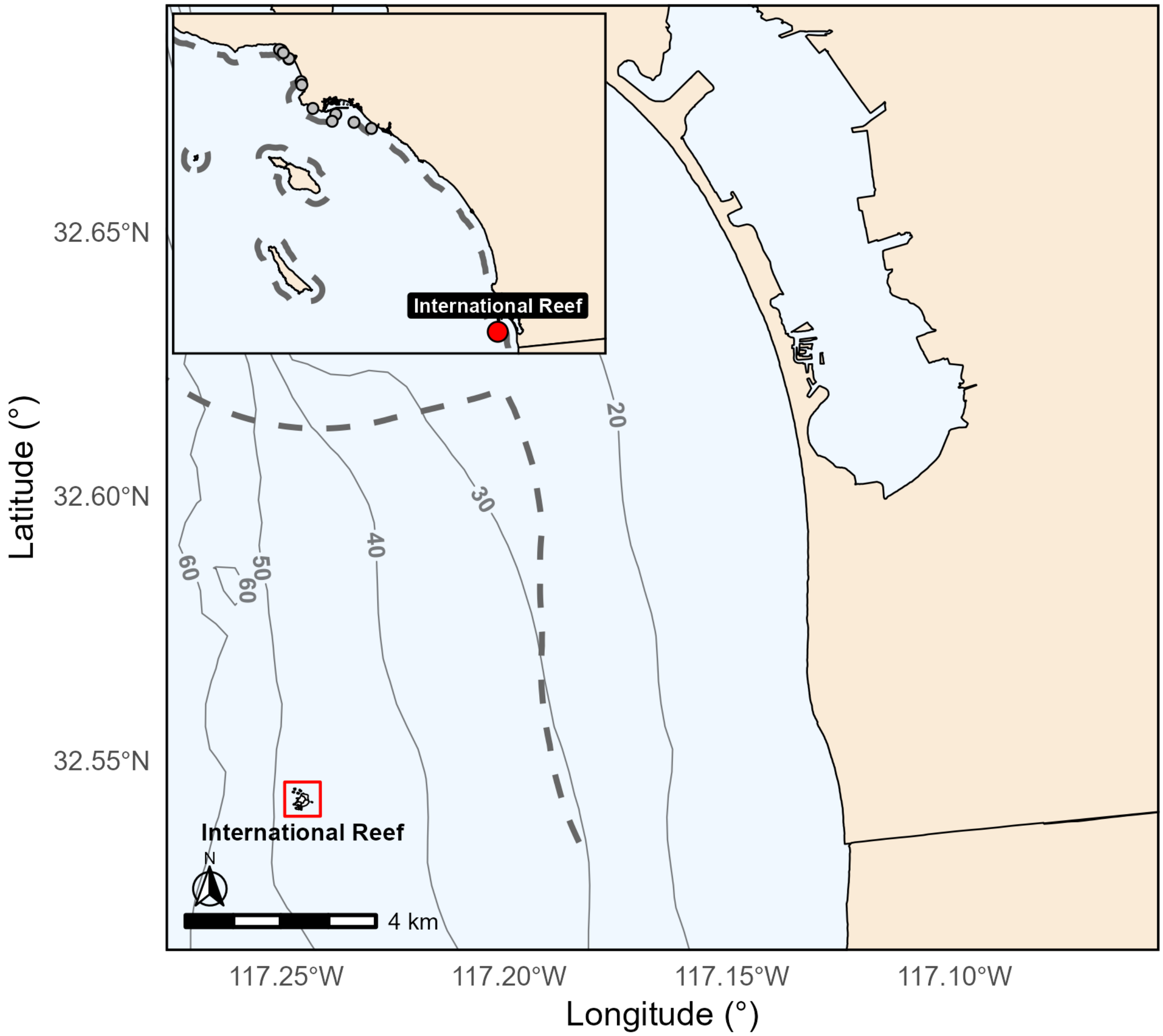

Figure A26: Location of International Reef Complex. The red bounding box indicates the area included in the AR module detail map (Figure A27). The boundary between state and federal waters (dashed line, not visible in main plot for some AR complexes) is generally located 3 nautical miles offshore. Bathymetry contours are shown in meters and were derived from the NOAA ETOPO 2022 15 Arc-Second Global Relief Model using the marmap R package.

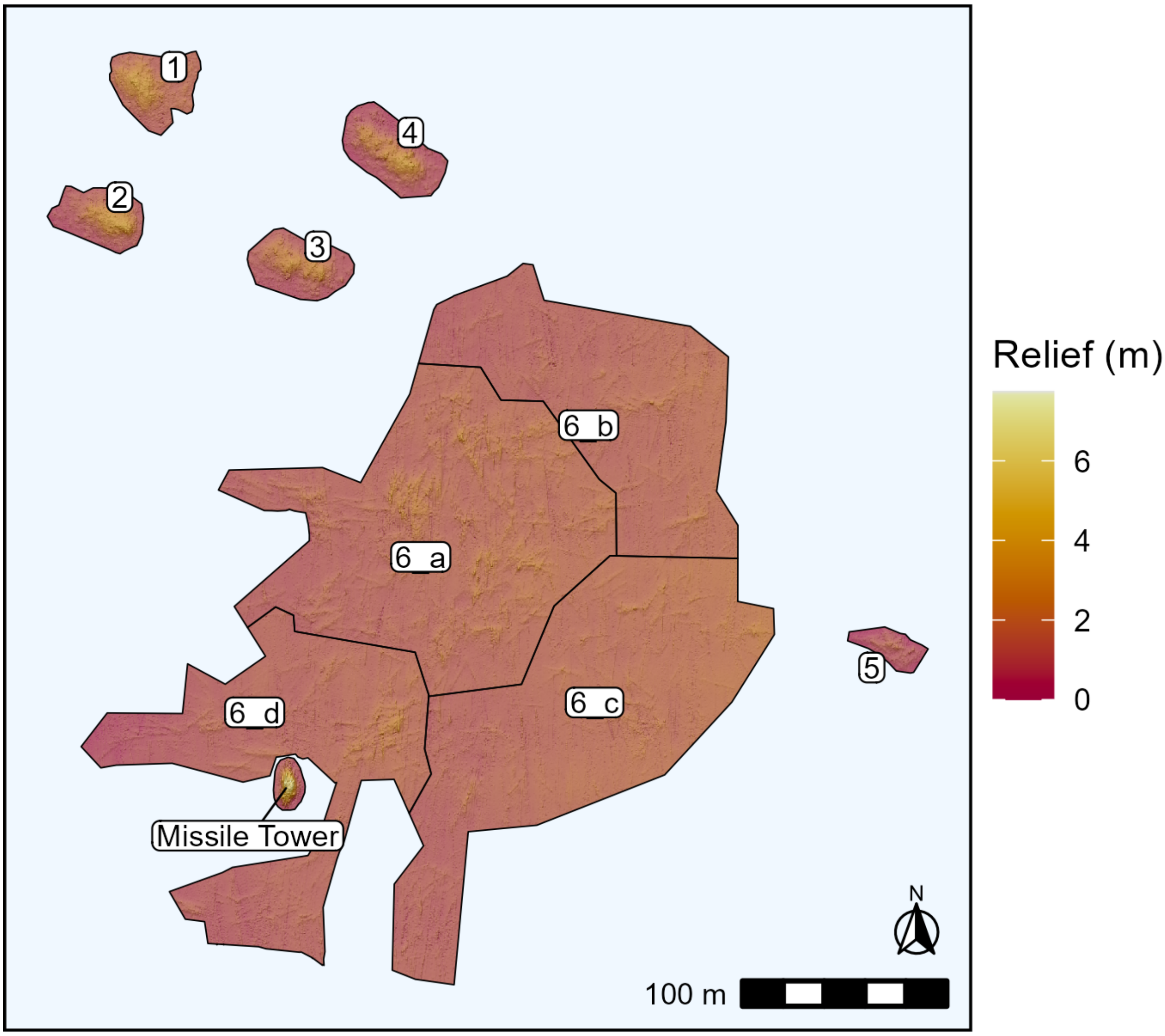

Figure A27: Map of the 10 module(s) in International Reef Complex. Sonar derived bathymetry was used to generate estimates of vertical relief in m (color).

Table A13: Characteristics of the 10 module(s) in International Reef Complex. Module naming attempted to preserve historical module spatial designations and names (Lewis and McKee 2001). The reported primary construction material was based on historical descriptions in the literature and that we confirmed from the sonar data or (where possible) visually during SCUBA or ROV surveys. Latitude and Longitude are center points of each module. Sonar derived bathymetry was used to generate estimates of average depth (m) and maximum vertical relief (m), while the 2d module footprint area was determined through a habitat classification process described in the methods. Note that for modules built from quarry rock, their areas are largely representative of the extent of contiguous material, while for most modules built from concrete material (e.g., pier pilings) these area estimates are more subjective, often inclusive of patches of soft sediment among scattered reef material. Frequently adjcent modules were originally classified as contigous reef habitat before being divided to maintain historical spatial designations and names (Lewis and McKee 2001).

Module	Material	Year Constructed (Augmented)	Latitude	Longitude	Depth (m)	Relief (m)	Foot-print (m^2)	Vol-ume (m^3)
1	quarry rock	1992	32.54462	-117.24804	48.3	2.6	1,240	1,004
2	quarry rock	1992	32.54404	-117.24832	48.4	3.3	1,077	1,159
3	quarry rock	1992	32.54382	-117.24729	48.0	2.4	1,268	1,028
4	quarry rock	1992	32.54433	-117.24680	47.7	3.1	1,448	1,299
5	quarry rock	2001	32.54215	-117.24419	46.7	1.5	474	249
6_a	concrete light poles	2001	32.54264	-117.24654	48.1	1.4	18,110	10,799
6_b	concrete light poles	2001	32.54322	-117.24567	47.8	1.1	11,349	6,107
6_c	concrete light poles	2001	32.54180	-117.24585	47.9	1.2	16,567	10,232
6_d	concrete light poles	2001	32.54163	-117.24735	48.6	1.6	11,866	8,771
Missile Tower	missile tower	1993	32.54153	-117.24733	47.2	5.9	317	527
